# PLSCR1 drives chemoresistance in TNBC via METTL3/IGF2BP3-mediated mRNA stabilization and EGFR-MAPK pathway activation

**DOI:** 10.1038/s41419-026-08845-4

**Published:** 2026-05-15

**Authors:** Yao Lu, Xueliang Zeng, Shixiong Peng, Yangyang Zhan, Wenyu Liu, Rui Zhao, Jing Li, Qiang Huang, Tingting Ye, Zixuan Yuan, Panpan Huang

**Affiliations:** 1 School of Basic Medicine, Gannan Medical University, Ganzhou, 341000 China; 2 Department of Pharmacy, The First Affiliated Hospital of Gannan Medical University, Gannan Medical University, Ganzhou, Jiangxi 341000 China; 3 School of Life Sciences, China Jiliang University, Hangzhou, 310018 China; 4 Department of Pharmacy, Shanghai Eastern Hepatobiliary Surgery Hospital, Navy Military Medical University, 225 Changhai Road, Yangpu District, Shanghai, China

**Keywords:** Prognostic markers, Breast cancer

## Abstract

Triple-negative breast cancer (TNBC) frequently acquires chemoresistance, leading to poor clinical outcomes. Phospholipid scramblase 1 (PLSCR1) has been implicated in breast cancer progression, yet its precise role and underlying mechanisms in TNBC chemoresistance remain elusive. Here, we demonstrate that PLSCR1 is significantly upregulated in chemoresistant TNBC cell lines and patient samples. Mechanistically, PLSCR1 interacts with EGFR, promoting its phosphorylation and subsequent activation of the MAPK signaling pathway, which in turn upregulates the efflux pumps P-gp and MRP1. Concurrently, PLSCR1 mRNA undergoes METTL3-mediated m6A modification, which is recognized by the m6A reader IGF2BP3, leading to enhanced mRNA stability and translational efficiency. Functional studies revealed that PLSCR1 knockdown resensitizes resistant cells to epirubicin, whereas its overexpression exacerbates resistance both in vitro and in vivo. Clinically, elevated PLSCR1 expression correlates with reduced sensitivity to neoadjuvant chemotherapy and poorer prognosis in TNBC patients. Notably, Mogroside IV-A, a specific PLSCR1 inhibitor, effectively overcomes chemoresistance by disrupting PLSCR1-mediated EGFR activation. Collectively, our findings establish PLSCR1 as a critical node integrating the METTL3/IGF2BP3 epigenetic axis with EGFR-MAPK signaling to drive TNBC chemoresistance, and highlight PLSCR1 as a promising therapeutic target for combating drug resistance in TNBC.

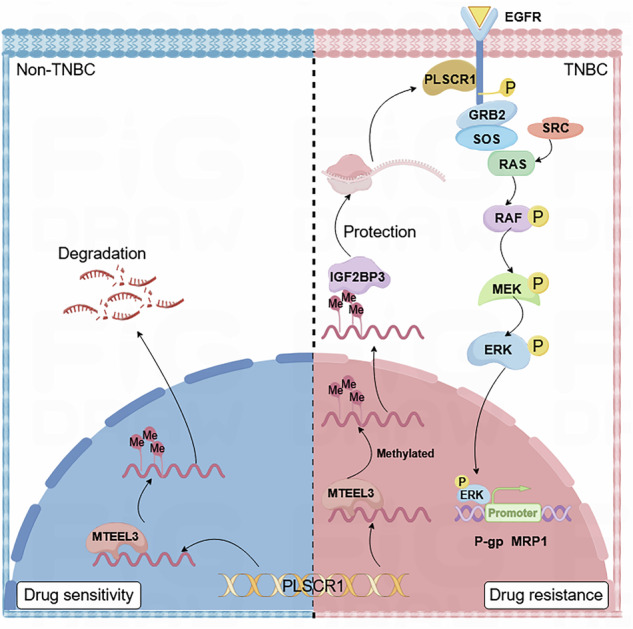

## Introduction

Triple-negative breast cancer (TNBC) represents the most aggressive molecular subtype of breast cancer, characterized by a lack of expression of estrogen receptor (ER), progesterone receptor (PR), and human epidermal growth factor receptor 2 (HER2). Due to the absence of effective targeted therapies, TNBC patients typically face a poor prognosis, with a five-year survival rate considerably lower than that of other subtypes [1, 2]. Current clinical treatment options for TNBC are neoadjuvant chemotherapy (NAC), followed by surgery and radiation therapy [3]. Standard NAC for TNBC typically includes anthracyclines (such as epirubicin) and taxanes, often combined with cyclophosphamide; however, the development of chemotherapy resistance remains a major challenge [4]. However, the frequent emergence of chemoresistance remains a major clinical obstacle, underscoring an urgent need to elucidate the underlying molecular mechanisms that could inform novel therapeutic strategies to improve patient outcomes.

Phospholipid scramblase 1 (PLSCR1) is a multifunctional protein involved in diverse cellular processes, including signal transduction, cell proliferation, apoptosis, autophagy, and transcriptional regulation [5–7]. Emerging evidence has linked PLSCR1 to cancer progression. For instance, Liao et al. reported that KPNA2 nuclear accumulation modulates radioresistance by positively regulating the PLSCR1-STAT1 feedback loop in lung adenocarcinoma [8]. In glioma, PLSCR1 promotes malignant progression via activation of the JAK-STAT3 signaling pathway [9]. Our previous work demonstrated that PLSCR1 is highly expressed in basal-like breast cancer (BLBC), where it drives tumor cell proliferation and metastasis. Notably, we also found that PLSCR1 enhances cancer stem cell properties, and TCGA data analysis revealed a significant correlation between PLSCR1 expression and drug resistance [10]. Given the substantial overlap between BLBC and TNBC, investigating the functional role of PLSCR1 in TNBC chemoresistance is of particular clinical relevance. Despite these insights, research on PLSCR1 in cancer biology remains limited, with few recent studies addressing its specific contributions to therapeutic resistance in breast cancer.

The epidermal growth factor receptor (EGFR) and its downstream mitogen-activated protein kinase (MAPK) signaling cascade are well-established drivers of TNBC progression and therapeutic resistance [11–13]. EGFR, a classical receptor tyrosine kinase, is aberrantly overexpressed or hyperactivated in 40–70% of TNBC cases, and its upregulation is closely associated with chemoresistance and poor prognosis [14]. Mechanistically, ligand-induced EGFR dimerization triggers autophosphorylation and subsequent activation of the RAS-RAF-MEK-ERK (MAPK) pathway, leading to the transcriptional upregulation of multidrug resistance proteins and enhanced DNA damage repair capacity [15, 16]. Furthermore, cancer-associated fibroblasts (CAFs) within the tumor microenvironment secrete cytokines such as IL-6, which activate the TGF-β/EGFR/MAPK axis in neighboring tumor cells via paracrine signaling, thereby propagating a signaling cascade of therapeutic resistance [17]. These intricate signaling networks partly account for the limited efficacy of single-agent EGFR inhibitors and highlight the necessity for combinatorial strategies that account for the spatiotemporal heterogeneity and pathway crosstalk underlying therapy resistance.

N6-methyladenosine (m6A) modification has emerged as a critical layer of epitranscriptomic regulation in cancer, dynamically modulating mRNA stability, splicing, export, and translation. The role of m6A in TNBC chemoresistance is an active area of investigation. Recent studies have demonstrated that in TNBC, an m6A-centered positive feedback loop involving LINC00115, SETDB1, and HIF1α promotes cancer stem cell phenotypes, driving chemoresistance and metastasis [18]. ALKBH5-driven m6A demethylation of FOXO1 mRNA promotes doxorubicin resistance in TNBC by enhancing FOXO1 expression and CSC maintenance, highlighting the ALKBH5/FOXO1 axis as a therapeutic target [19]. METTL3, as a key RNA methyltransferase, has long been a major focus of research. METTL3-mediated m6A modification promotes acquired cisplatin resistance in TNBC by upregulating DNA damage repair genes [20]. It is noteworthy that to overcome doxorubicin resistance, a targeted nano-system delivers METTL3 siRNA to degrade m6A modifications, thereby chemosensitizing tumors and enhancing therapeutic efficacy in resistant breast cancer [21]. Insulin-like growth factor 2 mRNA-binding protein 3 (IGF2BP3), a prominent m6A reader, has also been implicated in TNBC progression and therapy resistance. IGF2BP3 drives tumor progression and metastasis in an m6A-dependent manner [22, 23]. Cai et al. demonstrated that IGF2BP3 promotes TNBC stemness and carboplatin resistance by stabilizing FZD1/7 transcripts in an m6A-dependent manner, thereby activating oncogenic β-catenin signaling [24]. Additionally, IGF2BP3 enhances resistance to doxorubicin and mitoxantrone by regulating ABCG2 expression in TNBC cells [25]. These findings underscore the complexity of m6A-mediated regulatory networks and their critical contributions to chemoresistance, suggesting that further exploration may yield novel biomarkers and therapeutic strategies.

In this study, we demonstrate that high expression of PLSCR1 leads to enhanced resistance of TNBC, thereby significantly shortening the patients’ prognosis and survival. Mechanistically, PLSCR1 maintains its elevated expression in TNBC through a methylation-dependent mechanism of its mRNA (5’UTR 143 site) and promotes chemoresistance by activating the MAPK signaling pathway via interaction with EGFR (SER183). Furthermore, we also identified a small-molecule inhibitor of PLSCR1, Mogroside IV-A, which effectively reverses the drug tolerance of TNBC to epirubicin.

## Material and methods

### Informed consent and patient specimen collection

Tissues and pathological slides of breast cancer were obtained from the First Affiliated Hospital of Gannan Medical University. This study was approved by the Ethics Committee of the First Affiliated Hospital of Gannan Medical University and conducted in accordance with the Declaration of Helsinki. Written informed consent was obtained from each patient.

### IHC analysis using tissue microarray analysis (TMA)

TMA samples were collected from paraffin-embedded tissues obtained from 40 patients with MP: 1–2 and MP: 5 grades who received neoadjuvant chemotherapy. FFPE blocks were cored with a 0.6 mm needle at tumor-rich regions of interest (ROI) for TMA construction. IHC was performed for PLSCR1. Tissue sections were deparaffinized, retrieved antigens with citric acid solution, microwaved, and washed with PBS after cooling to room temperature. After blocking with 3% BSA, overnight incubation of the sections at 4 °C was done using an anti-PLSCR1 antibody.

### Reagents, shRNA, plasmids and antibodies

Dulbecco’s Modified Eagle’s Medium (DMEM) and L-15 media and Fetal bovine serum (FBS) were obtained from Gibco (NY, USA). Total RNA Extraction Reagent and Reverse Transcription Kit were supplied by NOVIZAN Biotechnology Company (Nanjing, China). The Annexin V-APC/7-AAD Apoptosis Detection Kit and goat anti-rabbit HRP-conjugated and goat anti-mouse HRP-conjugated secondary antibodies were provided by Liankebio (Hangzhou, China).

shRNA sequences were obtained from the MISSION shRNA library (Sigma-Aldrich) and synthesized by Youbao biological company (Hunan, China). Human PLSCR1 and EGFR genes were amplified from total cDNA of MDA-MB-231 cells and cloned into pLVX-Puro and pCMV-MCS vectors, respectively. shRNA and primer sequences were showed in Table S1.

The following antibodies were purchased from Proteintech (Chicago, IL, USA): anti-PLSCR1 (11582-1-AP), anti-c-SRC (60315-1-Ig), anti-phospho-c-SRC (Tyr530) (80706-2-RR), anti-IGF2BP3 (14642-1-AP), anti-MEK1/2 (11049-1-AP), anti-phospho-MEK1 (Thr292) (28930-1-AP), anti-ERK1/2 (11257-1-AP), anti-phospho-ERK1/2 (Thr202/Tyr204) (80031-1-RR), anti-EGFR (18986-1-AP), anti-phospho-EGFR (Tyr1069) (30277-1-AP), anti-METTL3 (15073-1-AP), anti-MRP1 (67228-1-Ig), anti-Ki-67 (27309-1-AP), and anti-P glycoprotein (67258-2-Ig). Anti-PLSCR1 (sc-518068) was obtained from Santa Cruz Biotechnology (Santa Cruz, CA, USA). The β-actin antibody (D191047) was purchased from Sangon Biotech (Shanghai, China).

Antioxidant peptide A (HY-P1512A), Mitoxantrone (HY-13502), AMXT-1501 (HY-124617), Dihydromyricetin (HY-N0112), CALP3 (HY-P1075) were obtained from MedChemExpress Co., Ltd (MCE, NJ, USA), Mogroside IV-A (HY-N6942) was obtained from Yuanye Bio-Technology Co., Ltd (Shanghai, China).

### Cell lines and culture

Human TNBC cell lines were obtained from the Chinese Academy of Sciences (Shanghai, China). Cell lines authentication was performed by STR profiling, and all lines tested negative for mycoplasma contamination prior to experiments. MDA-MB-231 and MDA-MB-436 cells were cultured in DMEM supplemented with 10% FBS. Stable cell lines were selected with 2 μg/mL puromycin for two weeks.

Epirubicin-resistant cells were established from parental lines by treatment with gradually increasing concentrations of epirubicin. Briefly, TNBC cells were exposed to epirubicin at an initial concentration of 1/40 th of the parental cells’ IC50 for 48 h. Once cells recovered, this cycle was repeated four times. After stable growth was achieved at this concentration, the epirubicin concentration was incrementally increased. This process continued until a resistance index (RI) >5 was reached. Resistant cells were maintained in complete medium containing 7 μM epirubicin. Prior to all functional assays, the epirubicin-resistant cells were cultured in drug-free complete medium for at least 10 days (approximately 3 passages) to eliminate residual drug effects. Following the washout period, the half-maximal inhibitory concentration (IC50) of epirubicin is re-determined for both the resistant and parental cell lines. The IC50 of the resistant cells should remain significantly higher than that of the parental cells. The dosage of small molecule compounds in cells is as follows: Antioxidant peptide A (10 μM), Mitoxantrone (10 μM), AMXT-1501 (20 μM), Dihydromyricetin (10 μM), CALP3 (10 μM), Mogroside IV-A (20 μM) and all worked for 24 h.

### 10×genomics single-cell RNA-sequencing

Two formalin-fixed breast carcinoma samples—LC_2314854_A10 (chemotherapy-insensitive, MP grade 5) and LC_2418716_A20 (chemotherapy-sensitive, MP grade 1)—were from the tissue bank of the First Affiliated Hospital of Gannan Medical University with ethical approval (IRB No. 2022209). Five-micrometer sections were stained with hematoxylin and eosin (H&E) for histopathological validation by a pathologist. Ten adjacent 10 μm sections per sample were processed for single-nucleus RNA sequencing (snRNA-seq) to mitigate FFPE-induced RNA fragmentation. Samples underwent nuclei isolation, single-nucleus RNA library preparation, sequencing, and bioinformatic analysis. Reagents included Liberase™ TH Research Grade (Roche Diagnostics Deutschland GmbH, 05401151001) and the Chromium Fixed RNA Kit (10x Genomics, 1000474). Sequencing was performed by Hangzhou Lianchuan Biotechnology (Hangzhou, China), and data were analyzed using Cell Ranger (https://support.10xgenomics.com/).

### m6A sequencing and RNA sequencing

Four experimental groups of MDA-MB-231-Edr cell lines (shNC, shMETTL3-1, shMETTL3-2, and shMETTL3-3) were harvested. Total RNA was isolated using TRIzol® Reagent (Thermo Fisher), followed by DNase I treatment. For each group, RNA was divided for m6A immunoprecipitation sequencing (m6A-seq) and whole-transcriptome sequencing (RNA-seq). The RNA samples underwent m6A-seq and RNA-seq library construction, sequencing, and bioinformatic analysis. These experiments were performed by Hangzhou Lianchuan Biotechnology (Hangzhou, China).

### RNA stability assay

Cells were seeded in 6-well plates. At 70–80% confluency, cells were treated with actinomycin D (ActD; 5 μg/ml). RT-qPCR assays were performed at 0, 2, 4, and 8 h after treatment [26].

### Methylated RNA immunoprecipitation assay

Total RNA of MDA-MB-231-Edr cells was extracted with TRIzol® Reagent (Thermo Fisher Scientific), followed by DNase I treatment (Turbo DNase, Ambion). RNA was purified using an RNA purification kit (61,006, Invitrogen, USA). Purified mRNA was then sonicated to obtain fragmented RNA, which was divided into immunoprecipitation (IP) and input control aliquots. For IP, A total of 2 μg of RNA was combined with 5 μg of anti-m6A monoclonal antibody (Clone RM318, RevMAb) for incubation in 500 μL IP buffer (50 mM Tris-HCl pH 7.5, 150 mM NaCl, 0.1% NP-40, 40 U/μL RNase inhibitor) for 2 h at 4 °C with rotation. Antibody-RNA complexes were captured by incubation with pre-washed Protein A/G magnetic beads (Pierce) for 1 hour. Beads were washed four times with IP buffer, and bound RNA was eluted using 6.7 mM N⁶-methyladenosine (New England Biolabs) in Tris-EDTA buffer. The eluted RNA was reverse transcribed and the resulting cDNA was analyzed by qPCR as previously described [27].

### Real time-qPCR (RT-qPCR)

Total RNA was isolated from TNBC cells or tissues using the FastPure Complex Tissue/Cell Total RNA Isolation Kit (Vazyme, China). RNA quality was confirmed using a NanoDrop spectrophotometer (Thermo Fisher Scientific, USA). Quantitative PCR was performed on a Real-Time PCR system (Dongsheng, China). Relative gene expression was calculated using the 2^−ΔΔCt^ method [28].

### Western blotting

Cells or tissues were lysed in Western/Ip or RIPA buffer added with protease and phosphatase inhibitors (Beyotime, China). Protein samples (30 μg per lane) were resolved on SDS-PAGE gels and then transferred onto PVDF membranes (0.45 μm, Millipore) using a VE186 transfer system (Tanon, China). Membranes were blocked in 5% non-fat dried milk for 40 min, then incubated overnight at 4 °C with primary antibodies. Signals were developed using BeyoECL Plus Substrate (Beyotime, China) and imaged using a Tanon imaging system (Tanon, China). Experiments were performed with three independent biological replicates.

### Co-immunoprecipitation (Co-IP)

Cells were transfected with 6 μg of either Flag-tagged PLSCR1 (PLSCR1-Flag) or His-tagged EGFR (EGFR-His) plasmids using ExFect Transfection Reagent (Vazyme, China). Seventy-two hours post-transfection, cells were collected in an ice-cold lysis buffer (Beyotime, China). For each immunoprecipitation reaction, 500 μg of cell lysate was pre-cleared with Protein A/G magnetic beads (Sigma, USA) for 1 h at 4 °C. The pre-cleared lysates were then incubated with 20 μL anti-Flag magnetic beads (Sigma, USA) overnight at 4 °C with rotation. Beads were washed four times with lysis buffer, and bound protein complexes were eluted. Input lysates and immunoprecipitation (IP) eluates were detected and analyzed by IB with anti-His or anti-Flag antibodies. All experiments were performed with three independent biological replicates.

### GST pull-down assay

TNBC cells were co-transfected with plasmids encoding GST-tagged PLSCR1 and His-tagged EGFR. After 48 h, cells were lysed in NP-40 lysis buffer. The clarified lysate was incubated with pre-washed Glutathione Sepharose 4B beads at 4 °C for 2 h. Beads were extensively washed with lysis buffer to remove non-specific binding. Bound proteins were eluted by boiling in 2× SDS loading buffer. Input lysates and eluted samples were analyzed by SDS-PAGE and immunoblotting using anti-GST and anti-His antibodies.

### Immunofluorescence (IF)

Cells and organoids underwent fixation using 4% paraformaldehyde for 30 min at RT, permeabilization with 1% Triton X-100 for 15 min, and blocked in 5% bovine serum albumin (BSA). Samples were incubated using primary antibodies against PLSCR1, pEGFR, METTL3, or IGF2BP3. After washed with PBS (5 min each, 3 times), species-matched secondary antibodies were applied: Alexa Fluor 488 goat anti-mouse (1:1000, Invitrogen, A11001) or Alexa Fluor 555 goat anti-rabbit (1:1000, Invitrogen, A21428) for 1 h at RT. Nuclei were counterstained with DAPI (1 μg/mL, 15 min). Images were acquired using a Zeiss LSM 900 confocal laser-scanning microscope.

### CCK8 assay

Cell supernatants were discarded, and the CCK-8 working solution (GLPBIO, USA), diluted tenfold in DMEM, was added. The plates underwent incubation at 37 °C for 1 hour, followed by absorbance measurement at 450 nm with a microplate reader. (Flash, China). The experiment was performed as previously described [29]. Dose-response curves were generated, and half-maximal inhibitory concentration (IC50) values were calculated by fitting the data to a four-parameter logistic (4PL) model (log(inhibitor) vs. response—Variable slope) using GraphPad Prism (version 8.0.1). The IC50 values are reported with their 95% confidence intervals (95% CI) in Figure legends.

### Apoptosis assays

Cells were treated with 15 μM epirubicin (Selleckchem, S1223) for 48 h. Both adherent and floating cells were collected, washed, and resuspended in 100 μL of 1× binding buffer. The Annexin V-APC/7-AAD Apoptosis Detection Kit was used to perform staining (MULTI SCIENCES, China). Samples were analyzed within 30 min using a BD FACSCanto II flow cytometer (BD, USA).

### Organoid model

1. Collection and Processing of Chemotherapy-Resistant TNBC Tissue Samples
  Fresh chemotherapy-resistant TNBC tissue samples were collected from The First Affiliated Hospital of Gannan Medical University. The detailed clinical annotation for the patient-derived organoid samples is provided in Supplementary Table 2.
2. Tissue Digestion and Organoid Culture Initiation
  (1) Digestion: Samples were incubated at 37 °C for 10–30 min after adding tissue digestion buffer to the pellet, until cells dissociated from tissue fragments. (2) Reaction Termination: Digestion was stopped by adding organoid buffer. (3) Filtration: The cell strainer was used. (4) Centrifugation: The supernatant was discarded. (5) Embedding: The pellets were resuspended by Matrigel and thoroughly mixed. (6) Seeding: The mixture was added into 24-well plate. (7) Culture: The mixture was maintained at 37 °C with 5% CO₂.
3. Lentiviral Transduction for shPLSCR1, shMETTL3, and shIGF2BP3
  (1) Viral Transduction: Organoid cell suspensions were mixed with lentiviral particles and Polybrene, then incubated at 37 °C for 6 h. (2) Medium Replacement: Fresh medium was added, and cultures were continued for 48 h. (3) Re-embedding: Transduced cells were mixed with Matrigel and seeded into pre-warmed plates. (4) Growth Factor Supplementation: Culture medium was supplemented with growth factors to restore 3D growth conditions. (5) Selection: Puromycin (4 μg/mL) was added for 5 days to select successfully transduced organoids. Drug-resistant organoids were expanded from two independent patient-derived organoid (PDO) lines (*n* = 2), each of which was regarded as an independent biological replicate. All experiments were performed between passages P3 and P8.

### Zebrafish model

Zebrafish were purchased from Hunter Biotech (China). The experiments were conducted in accordance with our previously published protocol [30]. Each zebrafish embryo received a microinjection of 400 cells that had been stained with 1 mg/mL CellTracker™ CM-DiI dye (Invitrogen, Waltham, MA, USA). Post-injection, the zebrafish were treated with epirubicin and maintained at a temperature of 34 °C. Zebrafish were immobilized in 0.2% low-melting-point agarose and then underwent microscopic imaging after four days. Zebrafish were randomly allocated into experimental groups. Each experimental group consisted of *n* = 7 zebrafish. Embryos exhibiting developmental defects or failed microinjection were excluded from analysis. All measurements were performed in a single-blinded manner, with the experimenter unaware of the group assignments. The survival rate was 100% across all groups at the analysis endpoint. Images were analyzed using ImageJ software.

### Nude mouse xenograft model

All animal experiments were approved by the Institutional Animal Care and Use Committee of China Jiliang University. To model tumor growth, 5 million cells were introduced into the mammary fat pad of female nude mice. Tumor measurements were taken every three days over a 24-day period, followed by recording their size and weight. Tumor tissues were analyzed by hematoxylin and eosin (HE) and immunohistochemistry (IHC) staining. In the metastasis model, female nude mice received an injection of 1 × 10⁶ cells into their tail vein. After 28 days, in vivo imaging was performed using the IVIS Lumina III system (PerkinElmer, Waltham, MA, USA). All mice received intravenous injections of either PBS or epirubicin (1.5 mg/kg/day) on days 8, 11, 14, and 17. The drug dosage was converted based on body surface area from the human equivalent dose. During the treatment period, mice were weighed twice weekly and monitored for general condition (activity, feeding, fur appearance). No significant treatment-related adverse effects or body weight loss were observed. All nude mice were randomly allocated into experimental groups. For the subcutaneous xenograft model, *n* = 6 nude mice per group were used. For in vivo imaging experiments, *n* = 3 mice per group were subjected to analysis. Lung tissues were sectioned and subjected to H&E staining. Images were captured under a microscope at ×200 magnification. The metastatic burden was quantified using ImageJ software by calculating the percentage of the total metastatic area relative to the total lung area in the analyzed fields. All measurements were performed in a single-blinded manner, with the experimenter unaware of the group assignments. Animals were excluded from the final analysis based on pre-established criteria: failed tumor engraftment, technical injection failures, early death unrelated to treatment, or other technical failures. All reported group sizes reflect only the animals that completed the entire study protocol.

### Immunohistochemistry (IHC)

IHC was performed on formalin-fixed, paraffin-embedded (FFPE) tissue sections. Briefly, 5 μm sections were deparaffinized, rehydrated, and subjected to antigen retrieval in citrate buffer (pH 6.0) using a microwave heating method. Endogenous peroxidase activity was blocked with 3% hydrogen peroxide. After blocking with 5% normal goat serum, sections were incubated overnight at 4 °C with primary antibodies. Subsequently, sections were incubated with a horseradish peroxidase (HRP)-conjugated secondary antibody for 1 hour at room temperature, followed by color development with 3,3’-diaminobenzidine (DAB) and counterstaining with hematoxylin. The percentage of positively stained cells and the staining intensity were analyzed using ImageJ software with the IHC Profiler plugin.

### Quantification and statistical analysis

Data are expressed as the mean ± standard deviation (SD). A two-tailed Student’s *t* test was used for two groups comparisons. One-way ANOVA was used for multiple groups with a single variable. Statistical analysis was performed using one-way analysis of variance (ANOVA) followed by Dunnett’s post-hoc test for multiple comparisons against the control group. Pearson’s correlation analysis assessed the relationship between PLSCR1 and stem cell markers, as well as between anti-tumor drugs and breast cancer cells. A *p* value < 0.05 was considered statistically significant.

### Bioinformatics analysis

**Fig. 1:**
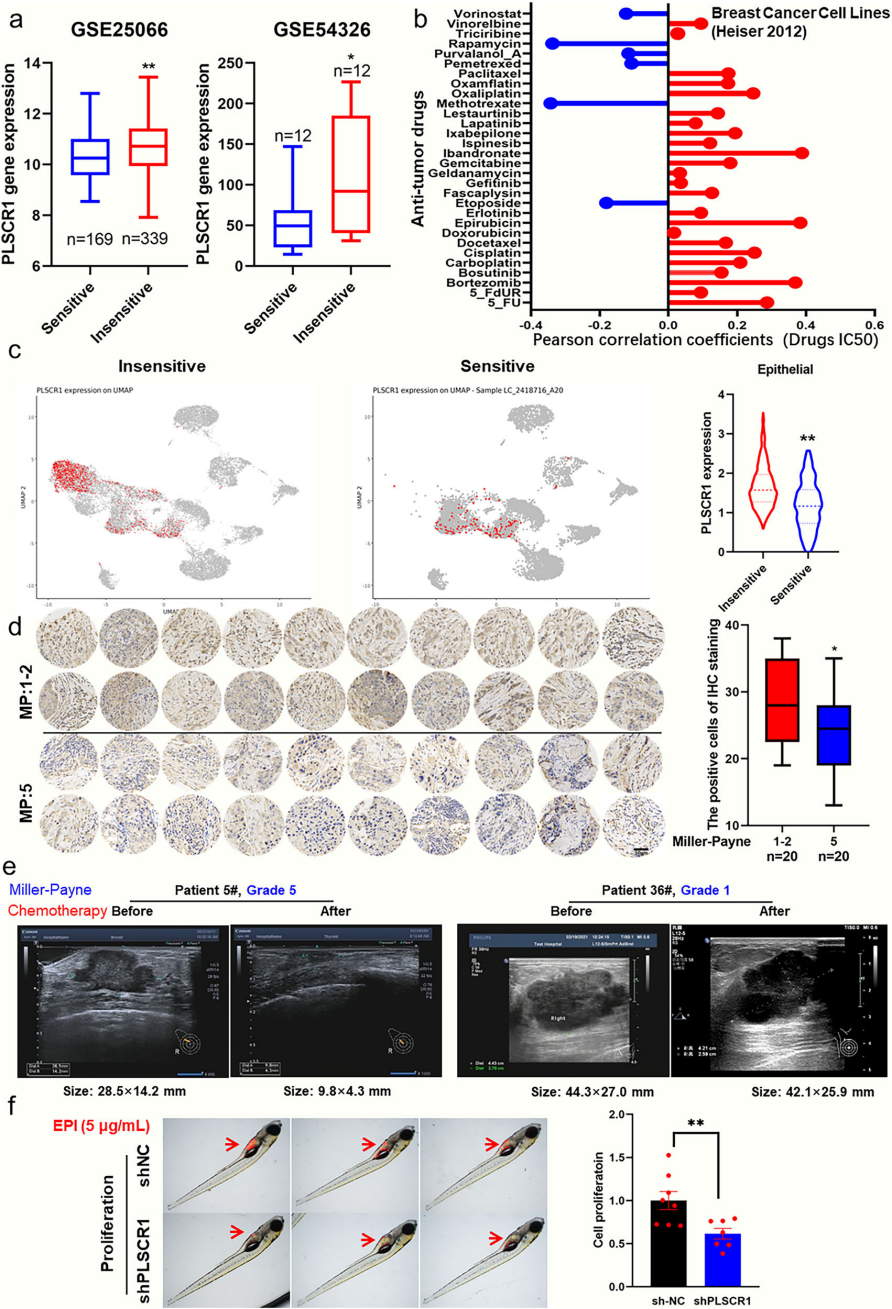
PLSCR1 is high expression in drug-resistant TNBC. **a** Analysis of PLSCR1 RNA level in TNBC tissue samples (GSE25066) and cell lines (GSE54326) on the basis of the GEO database. n: Sample size; **b** The correlation between the IC50 of various therapeutic drugs in breast cancer cells and the expression of PLSCR1 using UCSC Xena browser; **c** The differences of PLSCR1 RNA expression in neoadjuvant chemotherapy resistance and non-resistance through single-cell sequencing; **d** Immunohistochemical staining in tumor microarray tissues (Miller-Payne grading 1–2, *n* = 20 vs. 5, *n* = 20); **e** The tumor sizes (MP grading 1 vs. MP grading 5) were assessed by comparing the size before chemotherapy with the size after four cycles of chemotherapy using color Doppler ultrasound; **f** The proliferation of cells in zebra fish was observed under fluorescence microscope (Values are mean ± SD, *n* = 7/group). *, *P* < 0.05; **, *P* < 0.01.

Figure 1a data and Supplementary Fig. 1a were analysed using the GEO DataSets (GSE12791, GSE25066, GSE54326) via GEO2. (https://www.ncbi.nlm.nih.gov/). In supplementary Fig. 1d, For the AUC analysis, we stratified the GSE25066 cohort into resistant and sensitive groups, matched the expression of relevant genes, and performed the analysis using the diagnostic module of the www.home-for-researchers.com platform (https://www.home-for-researchers.com/#/). For Fig. 1b data, drug response data (IC50) for various breast cancer cell lines were obtained from the Breast Cancer Cell Lines (Heiser 2012) dataset within the XENA database. Associations were assessed using Pearson correlation coefficients. (http://xena.ucsc.edu/). Figure 4a data was generated using the SRAMP (http://www.cuilab.cn/sramp) online tool. Figure 5a data was generated using the TIMER2.0 (http://timer.cistrome.org/) online tool. In supplementary Fig. 1b, c, survival curves were generated using the Kaplan-Meier Plotter (https://kmplot.com/analysis/) online tool. Supplementary Fig. 4i data were obtained from DAVID (https://davidbioinformatics.nih.gov/). Supplementary Fig. 7a data was generated using the RM2 target (http://rm2target.canceromics.org/#/home) online tool. Supplementary Fig. 7h, i data were generated using the UALCAN (https://ualcan.path.uab.edu/cgi-bin/ualcan-res.pl) online tool.

## Results

### PLSCR1 is high expression in drug-resistant TNBC and correlates with poor prognosis

Our previous study showed that PLSCR1 induced stem cell properties and malignant progression in BLBC through STAT1 activation [10]. Given that cancer stem-like cells are linked to chemoresistance and poor patient outcomes, we analyzed gene expression datasets (GSE25066, GSE54326, and GSE12791) to explore the association between PLSCR1 and chemotherapy resistance in TNBC. We found that PLSCR1 expression was notably elevated in chemotherapy-resistant TNBC compared to those sensitive to chemotherapy (Fig. 1a and Fig. S1a). Similarly, using the Xena database, we examined the correlation between the IC50 values of various therapeutic drugs in breast cancer cell lines and PLSCR1 expression. For most drugs, higher PLSCR1 expression was associated with decreased sensitivity (higher IC50) in breast cancer cells (Fig. 1b). Additionally, TNBC patients after neoadjuvant chemotherapy with high PLSCR1 expression exhibited worse overall survival (OS) (Fig. S1b). PLSCR1 expression was also negatively correlated with disease-free survival (DFS) and relapse-free survival (RFS) in breast cancer patients (Fig. S1c). Based on GSE25066, we confirmed that PLSCR1 had higher diagnostic efficacy compared to those well-reported drug resistance genes (e.g., EGFR, MYC, RASAL2, and S100P) for the resistant cases by ROC analysis [13, 31–33] (Fig. S1d). Consistently, the ROC analysis of single-cell data demonstrated that PLSCR1 had diagnostic efficacy (Fig. S1e). To further explore this observation, we performed an exploratory single-nucleus RNA sequencing comparison using a limited sample set comprising one chemotherapy-resistant and one chemotherapy-sensitive TNBC patient sample. Within this preliminary analysis, we noted that cancer cells from the resistant sample exhibited higher PLSCR1 expression compared to the sensitive sample (Fig. 1c). Furthermore, in the resistant sample, PLSCR1 expression showed a positive correlation with the stemness-associated markers ALDH1A1 and CD44 (Fig. S1f). Further flow cytometry analysis revealed that high PLSCR1 expression in TNBC resistant cells significantly enhanced the fluorescent intensity of both CD44 and ALDH1A1 (Fig. S2a, b).

To further define the association between PLSCR1 expression and chemoresistance, we collected 40 tumor biopsy samples prior to the initiation of chemotherapy, classified by Miller-Payne (MP) grade (MP 1–2, *n* = 20; MP 5, *n* = 20), and assessed PLSCR1 protein levels by immunohistochemistry (IHC) on tissue microarrays. PLSCR1 levels were higher in tumors with MP grades 1–2 compared to those with grade 5 (Fig. 1d). From these TNBC samples, we selected representative cases with high PLSCR1 expression (Patient 36#, MP: grade 1) and low PLSCR1 expression (Patient 5#, MP: grade 5), and compared color ultrasound images before and after neoadjuvant chemotherapy. Imaging confirmed that patients with high PLSCR1 expression derived less benefit from neoadjuvant chemotherapy after (Fig. 1e). In a zebrafish model, PLSCR1 knockdown enhanced the sensitivity of MDA-MB-231 cells to epirubicin, resulting in greater suppression of proliferation and metastasis in vivo (Fig. 1f and Fig. S2c). Together, these results imply that PLSCR1 is highly present in chemotherapy-resistant TNBC and is associated with a negative prognosis.

### PLSCR1 inhibition enhances the sensitivity of TNBC cells to chemotherapy treatment

**Fig. 2:**
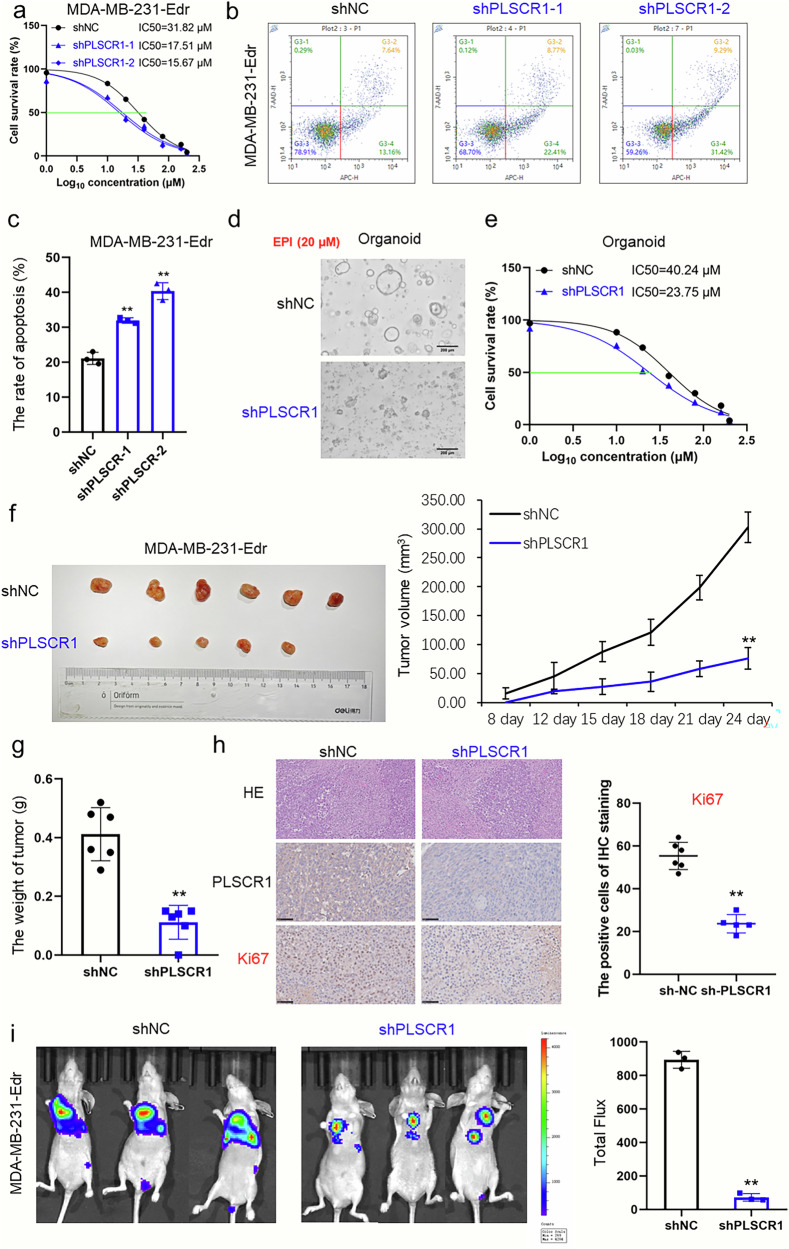
PLSCR1 knockdown increases the sensitivity of TNBC cells to epirubicin. **a** The IC50 value of epirubicin in MDA-MB-231-Edr cell line was tested by CCK8 (*n* = 8/group, shNC 95% CI: 28.27 to 35.73 μM, shPLSCR1-1 95% CI:11.63 to 24.06 μM, shPLSCR1-2 95% CI: 9.89–21.98 μM); **b** Apoptosis of MDA-MB-231-Edr was detected by flow cytometry; **c** Statistics of flow cytometry results (*n* = 3/group); **d** Pictures of organoid after epirubicin treatment; **e** The IC50 value of epirubicin in organoid (*n* = 8/group, shNC 95% CI: 33.28–48.47 μM, shPLSCR1 95% CI: 19.03–29.07 μM); **f** The proliferation curve of subcutaneous tumors in mice treated with epirubicin (*n* = 6/group); **g** the weight of subcutaneous tumors in mice treated with epirubicin; **h** The expressions of PLSCR1 and Ki67 in the subcutaneous tumors of mice were detected by IHC (*n* = 6/group); **i** PLSCR1 knockdown enhances epirubicin to inhibit tumor metastasis in vivo evaluation by live animal imaging technology. Values are mean ± SD. *, *P* < 0.05; **, *P* < 0.01.

To further investigate the effects of PLSCR1 on chemotherapy resistance, two epirubicin-resistant TNBC cell sublines (MDA-MB-231-Edr and MDA-MB-436-Edr) were established. As predicted, the epirubicin-resistant cells showed a marked increase in both mRNA and protein levels of PLSCR1. (Fig. S2d, e). Subsequently, we generated PLSCR1-knockdown (shPLSCR1) and overexpression (OE-PLSCR1) variants of these cell lines (Fig. S3a–c). The IC50 for epirubicin in MDA-MB-231-Edr and MDA-MB-436-Edr cells was assessed using the CCK8 assay. PLSCR1 knockdown significantly decreased the IC50, indicating enhanced sensitivity to epirubicin, while exogenous PLSCR1 overexpression resulted in a significant increase in IC50 (Fig. 2a and Fig. S3d).

Flow cytometry analysis indicated that apoptosis rates rose significantly in shPLSCR1 cell lines treated with epirubicin and fell in OE-PLSCR1 cell lines (Fig. 2b, c and Fig. S3e, f). Additionally, we constructed an organoid model from a primary drug-resistant TNBC sample and transduced it with lentiviral short hairpin RNAs targeting PLSCR1 (shPLSCR1) or an empty vector (shNC). CCK8 assays showed that knockdown of PLSCR1 significantly increased the organoid’s sensitivity to epirubicin (Fig. 2d, e and Fig. S3g).

We utilized a mouse xenograft tumor model to further investigate the role of PLSCR1 in vivo. The tumor’s growth was observed for a period of up to four weeks, until tumors reached the maximum allowable size. Notably, xenografts from MDA-MB-231-Edr cells with stable PLSCR1 knockdown exhibited reduced tumor growth following epirubicin treatment (Fig. 2f, g and Fig. S4a–c). Immunohistochemical analysis showed that expression of the proliferation marker Ki67 was markedly lower in PLSCR1-knockdown xenograft tumors (Fig. 2h and Fig. S4d, e), suggesting that reduced PLSCR1 expression enhances the suppressive effects of epirubicin on tumor growth in vivo. Finally, to evaluate the impact of PLSCR1 on metastasis, we used a xenograft metastasis model with MDA-MB-231-Edr cells, either with a stable empty vector or with PLSCR1 knockdown, to produce lung metastases. Consistently, PLSCR1 knockdown enhanced the inhibitory effect of epirubicin on lung metastasis in this mouse model (Fig. 2i and Fig. S4f, g). Collectively, these results indicate that decreased PLSCR1 expression reverses the resistance of epirubicin-resistant TNBC cells to epirubicin both in vitro and in vivo.

### PLSCR1 binding to EGFR activates MAPK pathway

PLSCR1, a membrane-associated protein, is involved in cell signaling, migration, differentiation, and proliferation [8, 34]. Given that PLSCR1 promotes epirubicin resistance in TNBC cells, we sought to investigate the underlying mechanisms. We first performed immunoprecipitation-mass spectrometry (IP-MS) experiments to identify membrane-associated signaling proteins that interact with PLSCR1 in TNBC cells. The results revealed a direct interaction between PLSCR1 and EGFR (Fig. S4h, i).

**Fig. 3:**
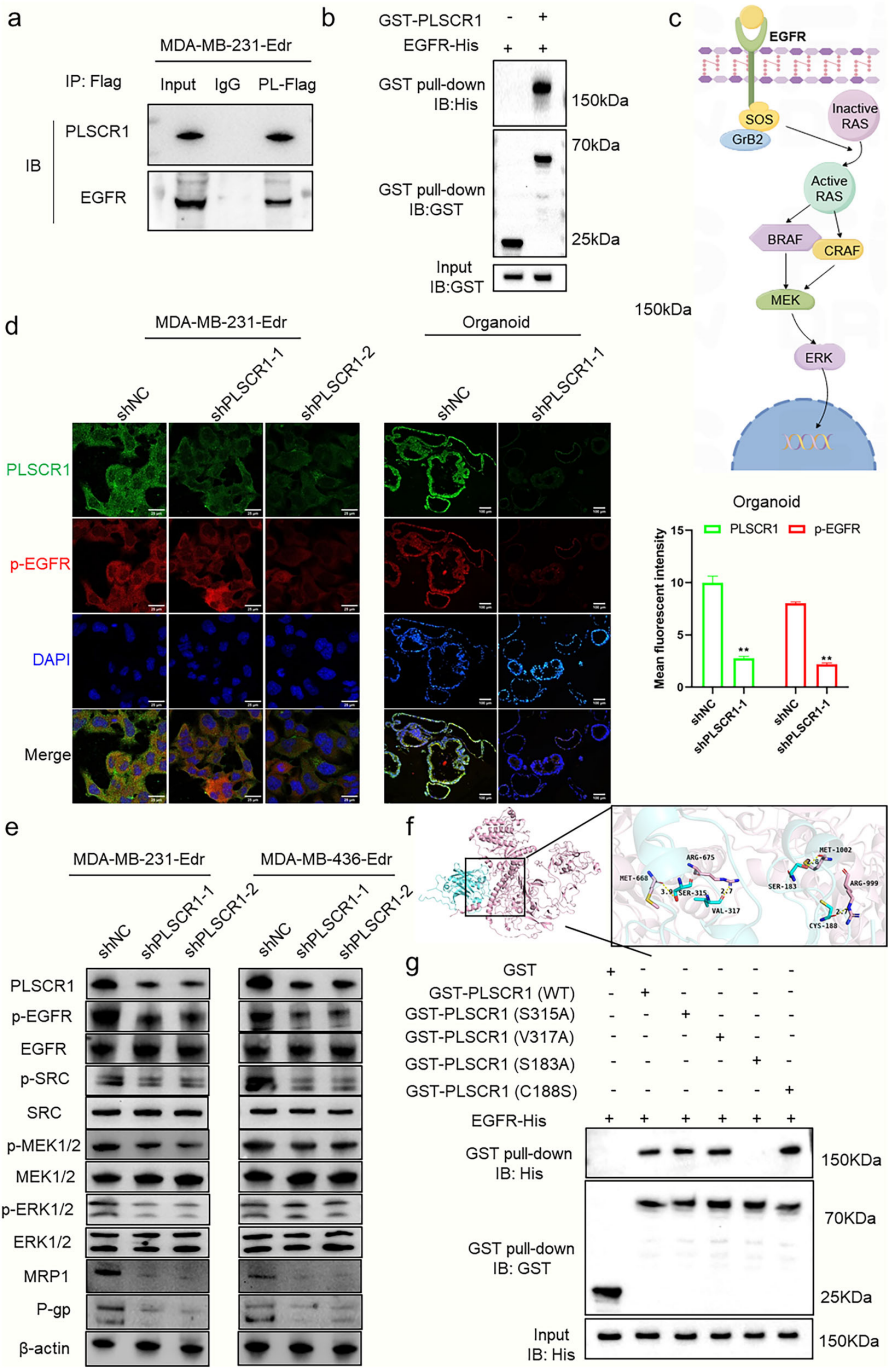
PLSCR1 binding to EGFR activates MAPK pathway. **a** The interaction relationship between PLSCR1 and EGFR detected by CoIP; **b** The interaction relationship between PLSCR1 and EGFR detected by GST-pull down; **c** Schematic diagram of EGFR-MAPK pathway; **d** The expression and co-localization relationship between PLSCR1 and pEGFR were tested in MDA-MB-231-Edr and organoid by confocal assay; **e** MAPK signaling pathway proteins and drug-resistant proteins were detected by Western Blot. Experiments were performed with three independent biological replicates. **f** Diagram of PLSCR1-EGFR molecular docking; **g** Identification of the key PLSCR1-EGFR binding sites by GST pull-down assay. Values are mean ± SD. *, *P* < 0.05; **, *P* < 0.01.

To further confirm this interaction, we co-expressed PLSCR1-3Flag and EGFR-6His in MDA-MB-231-Edr and MDA-MB-436-Edr cells, and conducted co-immunoprecipitation (Co-IP) and reverse Co-IP experiments in TNBC cells. Immunoprecipitation of PLSCR1 resulted in detection of EGFR, and reciprocal immunoprecipitation of EGFR also pulled down PLSCR1 (Fig. 3a and Fig. S5a, b), validating the interaction between the two proteins. Next, we constructed plasmids and obtained GST-PLSCR1 protein and EGFR-His protein by eukaryotic expression system, afterward, GST-pull down assays were performed. The results showed that there was a direct interaction between them (Fig. 3b and Fig. S5c).

EGFR, a transmembrane receptor, is activated by its endogenous ligand EGF, which leads to EGFR phosphorylation, subsequently activating the MAPK pathway and contributing to chemoresistance in breast cancer [35–37] (Fig. 3c). Notably, PLSCR1 knockdown resulted in a pronounced decrease in EGFR phosphorylation, as shown by confocal laser-scanning microscopy in resistant cell lines and organoids (Fig. 3d and Fig. S5d). The degree of interaction between PLSCR1 and phosphorylated EGFR (pEGFR) was quantified by densitometry using ImageJ software (Fig. S5e).

Consistently, western blot analysis demonstrated that PLSCR1 knockdown significantly decreased the levels of phosphorylated EGFR, MEK, and ERK (pEGFR, pMEK, and pERK) in epirubicin-resistant TNBC cells (Fig. 3e). Conversely, exogenous PLSCR1 expression restored phosphorylation of these signaling components (Fig. S5f). These findings suggest that PLSCR1 binds to EGFR and affects its phosphorylation, thereby activating the downstream MAPK pathway. Importantly, downregulation of PLSCR1 also suppressed the expression of P-gp and MRP1, two key resistance proteins regulated by the MAPK pathway [38, 39] (Fig. 3e). To further bolster this conclusion, we first knocked down four classical ERK effectors—c-Myc, c-Fos, c-Jun, and CREB—using siRNA and then measured the transcriptional levels of *P-gp* and *MRP1* (Fig. S6a). The results revealed that knockdown of either c-Fos or c-Jun significantly inhibited the transcription of *MRP1* (Fig. S6b). Conversely, the mRNA level of *P-gp* remained largely unchanged following the knockdown of any individual transcription factor (Data not shown). Collectively, these data suggest that c-Fos and c-Jun likely function by forming a transcriptional complex (e.g., AP-1), thereby specifically enhancing the transcriptional efficiency of the *MRP1* gene. Dual-luciferase reporter assays further validated our conclusion: the luciferase activity was significantly higher in MDA-MB-231 cells with concurrent overexpression of c-Fos and c-Jun than in those with individual overexpression of either gene (Fig. S6c). We subsequently predicted their potential binding sites using the JASPAR database and performed dual-luciferase reporter assays with different point mutations. These experiments confirmed that c-Fos/c-Jun binds to the *MRP1* promoter region (-65bp *TGACTCA* -59bp) (Fig. S6d). In order to further define the amino acid binding site of the PLSCR1 protein with the EGFR protein, the HDCOK program was utilized to predict the molecular docking between the PLSCR1 and EGFR proteins (Fig. 3f). The results showed that four hydrogen bond interactions were identified between the PLSCR1 and EGFR proteins, with residues SER315, VAL317, SER183, and CYS188 of PLSCR1 contributing to these bonds (Fig. 3f). As a next step, we individually mutated each of these sites (S315A, V317A, S183A, C188S), then, we detected the binding relation between PLSCR1 and EGFR by Co-IP and GST-pull down assays. The results showed that the PLSCR1-EGFR binding was significantly impaired when SER183 was mutated into ALA (Fig. 3g and S6e). As expected, compared to mutations at other sites, overexpression of the S183 mutant of PLSCR1 did not increase the phosphorylation level of EGFR (Fig. S6f). Stemming from these findings, we demonstrate that PLSCR1 binds to EGFR through the critical site S183, thereby promoting its phosphorylation.

### METTL3 stabilizes PLSCR1 expression via m6A methylation modification in epirubicin-resistant TNBC cells

Our previous studies demonstrated that PLSCR1 is highly expressed in TNBC cell lines (MDA-MB-231, MDA-MB-468, HCC1937) and promotes the proliferation, migration, and invasion of these cells both in vitro and in vivo [10]. However, the mechanism responsible for the high expression of PLSCR1 in TNBC remains to be elucidated. Abnormal RNA methylation is known to occur during the development of various tumors, including TNBC, and can affect tumor proliferation, metastasis, and drug resistance [19, 40].

**Fig. 4:**
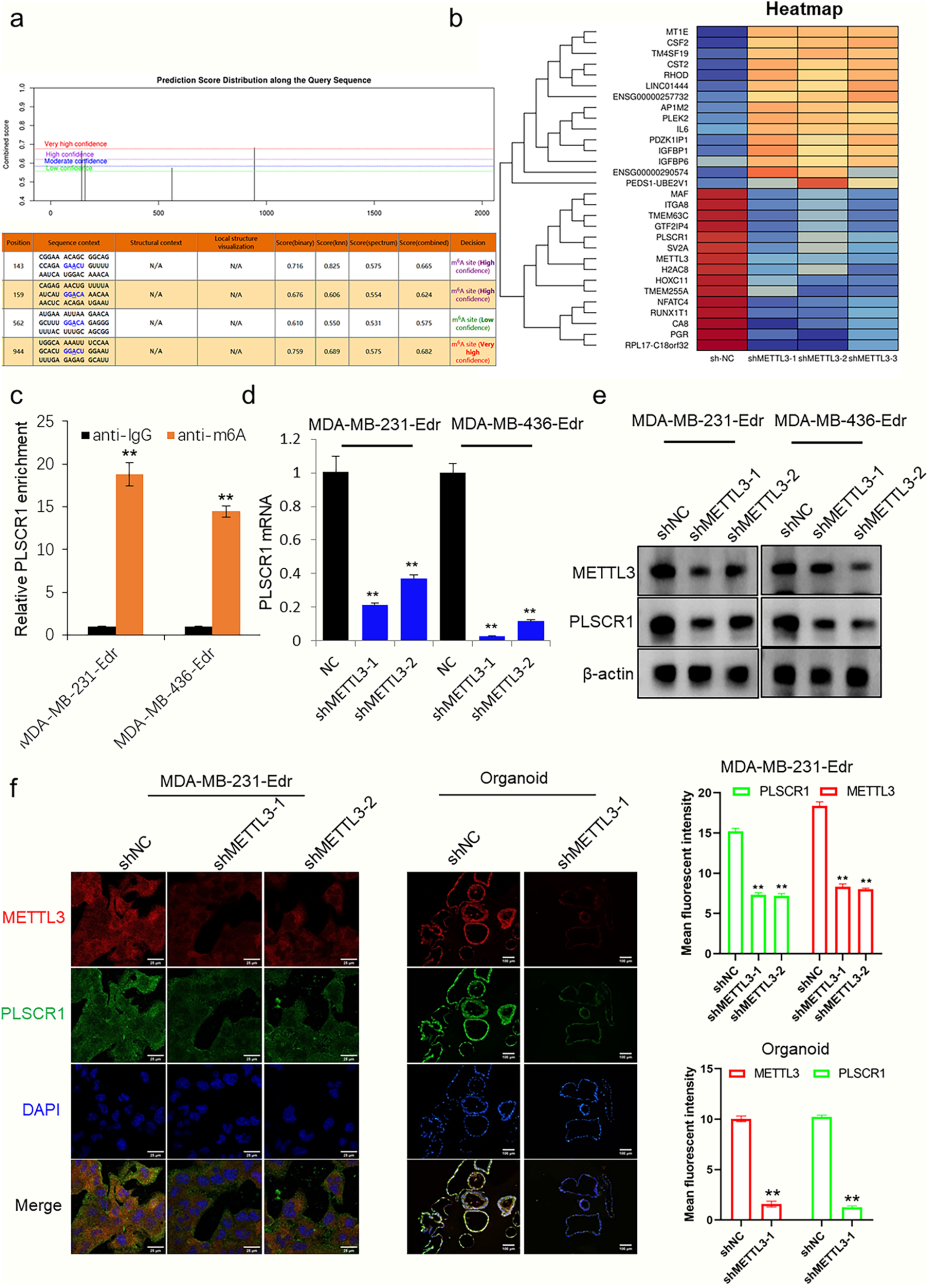
METTL3 stabilizes PLSCR1 expression via m6A methylation modification. **a** SRAMP (http://www.cuilab.cn/sramp) analysis the m6A methylation modifications of PLSCR1; **b** Heat maps of differential genes by RNA-seq; **c** The methylated RNA Immunoprecipitation assay using m6A-specific antibody; **d**, **e** The mRNA and protein expressions of PLSCR1 were detected after METTL3 knockdown; **f** The expression relationship between PLSCR1 and METTL3 were tested in MDA-MB-231-Edr and organoid by confocal assay (Values are mean ± SD, *n* = 3/group). *, *P* < 0.05; **, *P* < 0.01.

To explore this, we first utilized the sequence-based m6A modification site predictor SRAMP and found PLSCR1 mRNA contains multiple predicted m6A methylation sites (Fig. 4a). Next, analysis of MeRIP-seq datasets of MDA-MB-231 cells downloaded from the GEO database revealed that PLSCR1 mRNA is enriched for m6A methylation (Fig. S7a).

To investigate further, we performed m6A RNA methylation sequencing and RNA-sequencing in MDA-MB-231-Edr with METTL3 knockdown and control. The results showed that both m6A methylation levels and expression of PLSCR1 were reduced upon METTL3 knockdown (Fig. 4b and Fig. S7b). Additionally, the methylated RNA immunoprecipitation test with an m6A-specific antibody verified that PLSCR1 mRNA was highly enriched in the immunoprecipitate from MDA-MB-231-Edr cells, whereas control IgG did not enrich for PLSCR1 (Fig. 4c).

We examined PLSCR1 expression in epirubicin-resistant TNBC cells following METTL3 knockdown. As expected, both mRNA and protein levels of PLSCR1 were significantly downregulated after METTL3 knockdown (Fig. 4d, e and Fig. S7c). This effect was also observed in resistant cell lines and organoids, as shown by confocal microscopy (Fig. 4f and Fig. S7d).

These results collectively confirm that METTL3 acts as an essential methyltransferase that promotes m6A methylation of PLSCR1 mRNA, thereby maintaining PLSCR1 expression. Interestingly, we found that METTL3 mRNA and protein levels did not differ between parental and epirubicin-resistant strains (Fig. S7e). Beside, MeRIP-qPCR assays demonstrated that the m6A methylation enrichment level of *PLSCR1* in drug-resistant cell lines was unaltered compared with that in parental cell lines; however, knockdown of *METTL3* in drug-resistant cell lines led to a significant reduction in this enrichment level (Fig. S7f). This indicates that the methylation level of PLSCR1 exhibits no significant difference between drug-resistant cell lines and parental cell lines; however, METTL3—as the methyltransferase of PLSCR1—exerts a decisive regulatory effect on its expression.

### IGF2BP3 promoted PLSCR1 expression by maintaining its mRNA stability

Our results as shown before demonstrated that METTL3 was an important regulator of PLSCR1 expression in TNBC cells. However, we found the TCGA database and molecular assays (data not shown) revealed no significant differences in METTL3 expression across different molecular subtypes of breast cancer and cell lines. This suggests that variations in METTL3 levels do not account for differences in PLSCR1 expression among breast cancer subtypes.

**Fig. 5:**
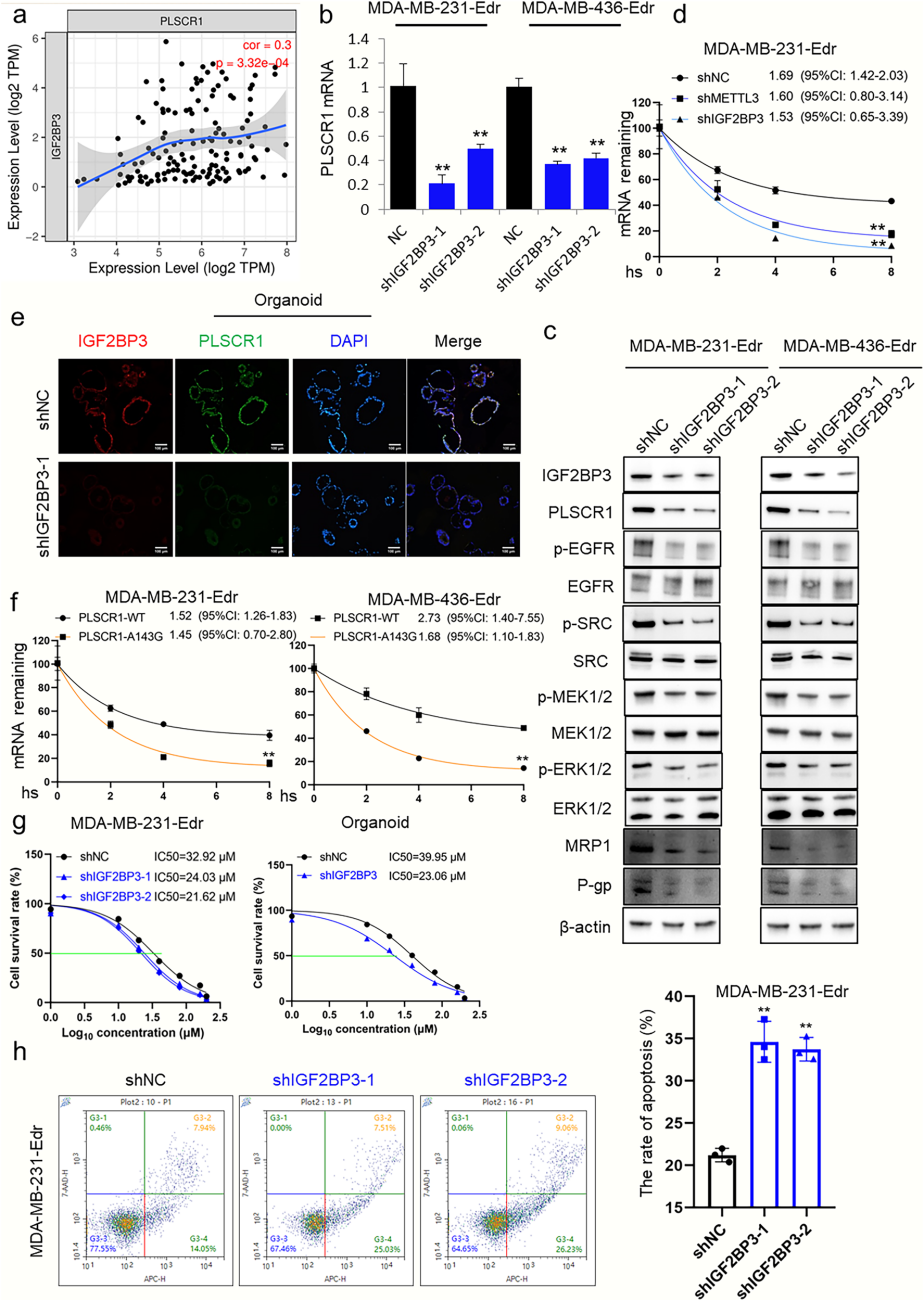
IGF2BP3 promoted PLSCR1 expression by maintaining its mRNA stability. **a** The co-expression relationship between PLSCR1 and IGF2BP3 was analyzed by TIME software; **b** The mRNA of PLSCR1 was detected after IGF2BP3 knockdown; **c** Western Blot assays tested the protein levels of PLSCR1 and MAPK signaling; **d** mRNA stability experiment; **e** The expression and co-localization relationship between PLSCR1 and IGF2BP3 were tested in organoid by confocal assay (*n* = 3/group); **f** mRNA stability assay of the A143 site mutant; **g** The IC50 value of epirubicin in MDA-MB-231-Edr and organoid were tested by CCK8 (*n* = 8/group, 231-shNC 95% CI: 27.15–39.66 μM, 231-shIGF2BP3-1 95% CI:19.51–29.14 μM, 231-shIGF2BP3-2 95% CI:17.84–25.78 μM, O-shNC 95% CI:32.12–49.24 μM, O-shIGF2BP3 95% CI:17.24 to 29.62 μM); **h** Apoptosis of MDA-MB-231-Edr was detected by flow cytometry (*n* = 3/group). Values are mean ± SD *, *P* < 0.05; **, *P* < 0.01.

Interestingly, MeRIP-seq data from the GEO database indicated strong enrichment of the m6A reader protein IGF2BP3 at the PLSCR1 mRNA (Fig. S7a), and PLSCR1 was significantly co-expressed with IGF2BP3 in basal-like breast cancer (Fig. 5a). This led us to hypothesize that IGF2BP3 binds to the m6A site of PLSCR1 mRNA, thereby promoting its stability.

To test this hypothesis, we performed RNA pulldown assays followed by mass spectrometry, which confirmed robust binding between PLSCR1 mRNA and IGF2BP3 (Fig. S7g). Additionally, analysis of TCGA data showed that the levels of both IGF2BP3 protein and mRNA were significantly elevated in TNBC compared to other types of breast cancer (Fig. S7h, i). Expression of IGF2BP3 mRNA and protein were also significantly higher in epirubicin-resistant TNBC cell lines than in parental cell lines (Fig. S7e).

To gain more insight into the regulatory connection between IGF2BP3 and PLSCR1, we generated epirubicin-resistant TNBC cells with stable IGF2BP3 knockdown. Knockdown of IGF2BP3 resulted in a significant decrease in both the mRNA and protein levels of PLSCR1, but did not affect the alterations in its m6A methylation level (Fig. 5b, c and Fig. S7f, j). Moreover, actinomycin D assays showed that knockdown of either METTL3 or IGF2BP3 reduced PLSCR1 mRNA stability (Fig. 5d and Fig. S7k). Confocal microscopy further confirmed that IGF2BP3 knockdown diminished PLSCR1 expression in both resistant cell lines and organoids (Fig. 5e and Fig. S8a–c). To further define the RNA methylation sites of PLSCR1 mRNA, we refered to SRAMP website (Fig. 4a) and analyzed the Sop of MeRIP-seq with IGV. The result showed the 143 site (in 5’UTR) has a very high confidence. So, we designed 143 site-mutated (A → G) PLSCR1 overexpressed and non-mutated PLSCR1 overexpressed plasmids (PLSCR1-WT, PLSCR1-A143G) and avoided shRNA effect through synonymous mutation. Next, we constructed two PLSCR1-overexpressing cell lines on the basis of shPLSCR1 cell lines (PLSCR1-WT, PLSCR1-A143G), respectively. mRNA stability assays demonstrated that the mutation of 143 site accelerated mRNA degradation (Fig. 5f). Furthermore, to definitively establish METTL3 as the writer enzyme for PLSCR1 mRNA and to validate the functional significance of the A143 site, we conducted a key MeRIP-qPCR experiment (PLSCR1-WT+Vec, PLSCR1-WT + OE-METTL3, PLSCR1-A143G + OE-METTL3). The results showed that overexpression of METTL3 in PLSCR1-WT cells led to a significant increase in m6A enrichment on PLSCR1 mRNA compared to the control, confirming METTL3’s direct role in its methylation. Crucially, this METTL3-driven hypermethylation was substantially abolished in cells expressing the PLSCR1-A143G transcript. The m6A levels in the mutant group remained near basal levels, despite the presence of excess methyltransferase. This experiment solidifies the upstream event in the proposed regulatory axis, where METTL3-dependent methylation at A143 creates the molecular landmark for subsequent IGF2BP3 binding and mRNA stabilization (Fig. S8d). Moreover, the mRNA of PLSCR1 did not bind the IGF2BP3 protein in PLSCR1-A143G cell lines through RNA pulldown-Western Blot assay (Fig. S8e). The two experiments presented above provide clear evidence that 143 site is the RNA methylation sites of PLSCR1 mRNA.

Collectively, these findings indicate that the basal m6A modification level of PLSCR1 mRNA in resistant cells remains unchanged (mediated by constitutively expressed METTL3), while its upregulated expression is primarily driven by the overexpressed reader protein IGF2BP3, which binds to and stabilizes the methylated transcript.

### IGF2BP3 regulated PLSCR1 expression activates MAPK pathway, promoting the proliferation of TNBC epirubicin-resistant cells in vitro and in vivo

Since the association between PLSCR1 and EGFR is implicated in the activation of the MAPK pathway, we assessed both total and phosphorylated levels of key downstream MAPK signaling proteins in IGF2BP3-knockdown MDA-MB-231-Edr cells. The results showed that IGF2BP3 knockdown reduced MAPK pathway activation and decreased the expression of P-gp and MRP1 (Fig. 5c). Consistently, CCK8 assay data confirmed that downregulation of IGF2BP3 significantly lowered the IC50 for epirubicin in both epirubicin-resistant TNBC cells and organoids (Fig. 5g and Fig. S8f). In agreement, flow cytometry indicated that IGF2BP3 knockdown significantly increased apoptosis in epirubicin-treated resistant cells (Fig. 5h and Fig. S8g).

**Fig. 6:**
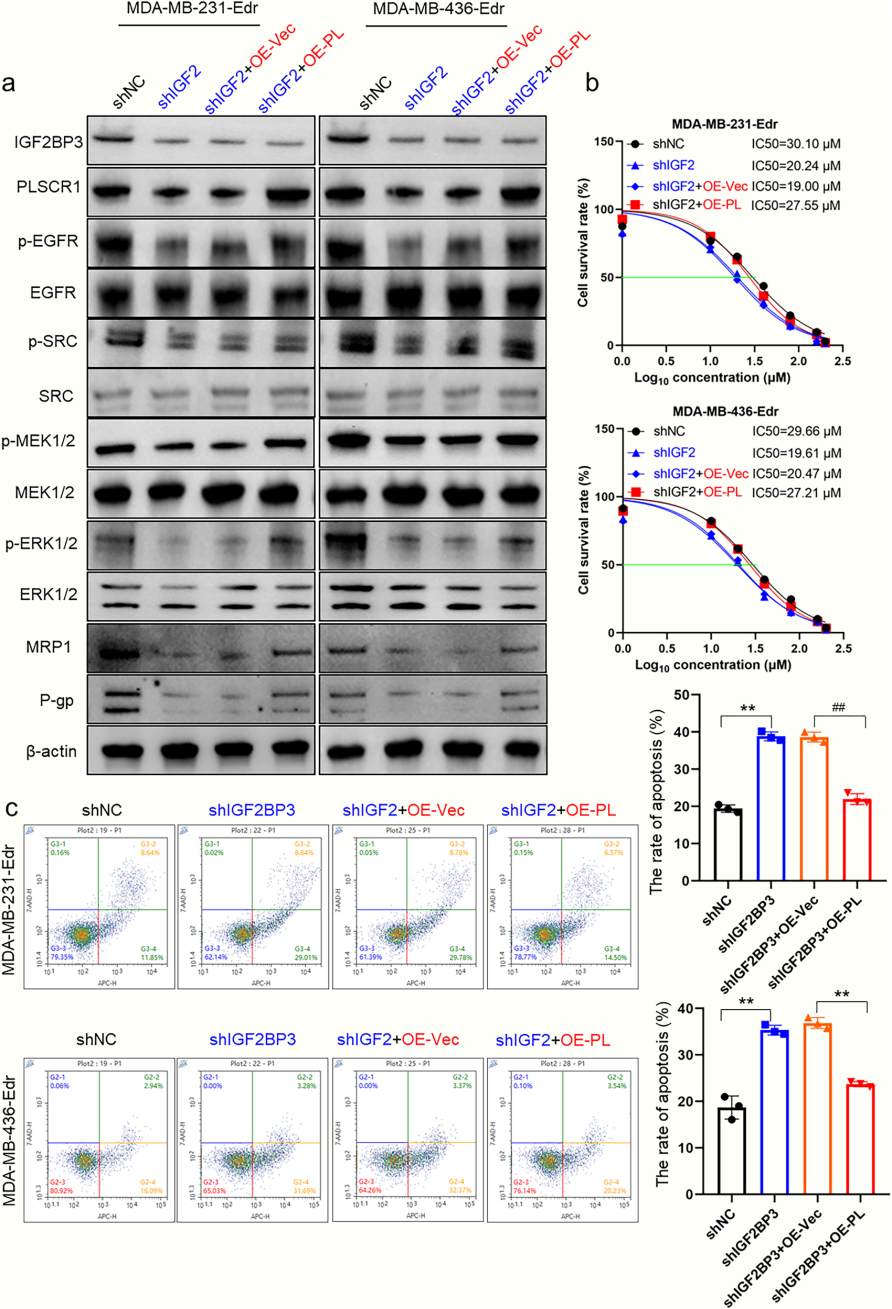
IGF2BP3 activates the MAPK pathway by enhancing PLSCR1 expression. **a** The effect of IGF2BP3 on the protein expression of PLSCR1 and the MAPK pathway detected by Western Blot; **b** The influence of IGF2BP3 on the IC50 of epirubicin in MDA-MB-231/436-Edr was tested by CCK8 (*n* = 8/group, MDA-MB-231-Edr -95% CI: 21.94–39.89, 12.45–28.73, 10.87–27.60, 23.54–32.05 μM, MDA-MB-436-Edr -95% CI: 24.26–35.93, 12.43–27.33, 12.23–29.36, 21.97–33.28 μM); **c** Flow cytometry was used to detect the effect of IGF2BP3 on MDA-MB-231/436-Edr apoptosis after epirubicin treatment (*n* = 3/group). Values are mean ± SD *, *P* < 0.05; **, *P* < 0.01.

Moreover, IGF2BP3 knockdown inhibited, while exogenous PLSCR1 expression restored the expression of key phosphorylated MAPK pathway proteins in MDA-MB-231-Edr cells (Fig. 6a). IGF2BP3 knockdown resulted in a marked decrease in IC50 for epirubicin and elevated apoptosis in both MDA-MB-231-Edr and MDA-MB-436-Edr cells (Fig. 6b). Importantly, exogenous PLSCR1 expression reversed these effects, restoring both IC50 and apoptosis rates to levels similar to control, even in cells with stable IGF2BP3 knockdown (Fig. 6c). Similar findings were observed in organoid models (Fig. S8h).

**Fig. 7:**
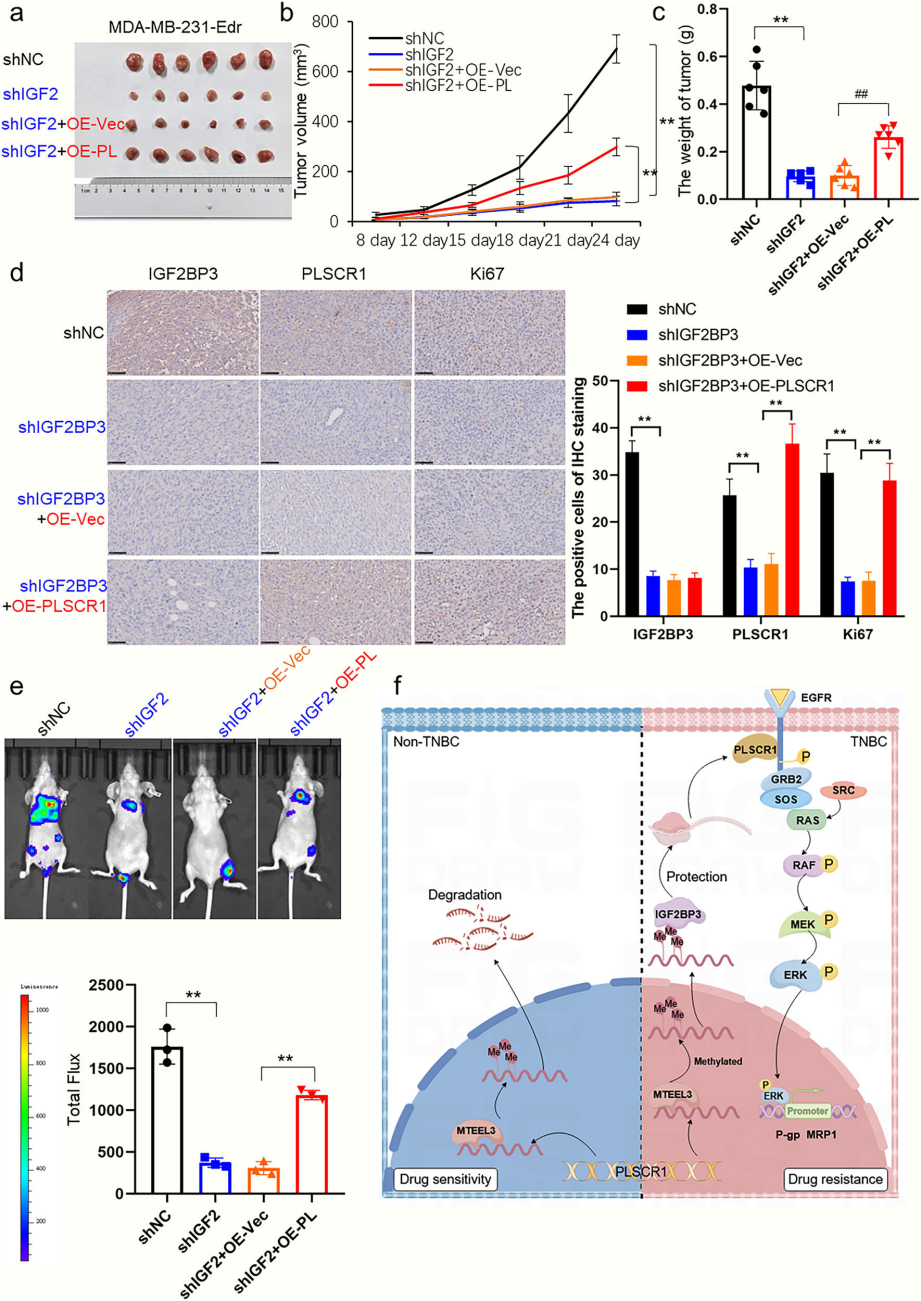
IGF2BP3 impairs the sensitivity of TNBC-resistant strains to epirubicin. **a**, **b** The proliferation curve of subcutaneous tumors in mice treated with epirubicin (*n* = 6/group); **c** The weight of subcutaneous tumors in mice treated with epirubicin (*n* = 6/group); **d** The expressions of IGF2BP3 and Ki67 in the subcutaneous tumors of mice were detected by IHC (*n* = 6/group); **e** PLSCR1 knockdown enhances epirubicin to inhibit tumor metastasis in vivo evaluation by live animal imaging technology (*n* = 3/group). **f** Mechanism diagram. Values are mean ± SD *, *P* < 0.05; **, *P* < 0.01.

To further explore the effect of IGF2BP3-regulated PLSCR1 expression on tumorigenicity in vivo, we overexpressed PLSCR1 in shIGF2BP3-expressing MDA-MB-231-Edr and MDA-MB-468-Edr cells. These and corresponding control cells were implanted into nude mice to create a xenograft tumor model (Fig. 7a and Fig. S9a). Tumor growth was significantly reduced in mice implanted with IGF2BP3-knockdown cells, whereas PLSCR1 overexpression markedly rescued the reduced tumor growth (Fig. 7b, c and Fig. S9b, c). Immunohistochemical analysis showed dramatic decreases in IGF2BP3, PLSCR1, and the proliferation marker Ki67 in IGF2BP3-knockdown tumors, while PLSCR1 overexpression significantly increased Ki67 expression (Fig. 7d and Fig. S9d).

In xenograft pulmonary metastasis models, knockdown of IGF2BP3 in MDA-MB-231-Edr and MDA-MB-468-Edr cells reduced lung metastasis, whereas PLSCR1 overexpression reinstated metastatic capacity (Fig. 7e and Fig. S9e). Collectively, these results indicate that IGF2BP3 positively regulates PLSCR1 expression, thereby promoting proliferation and metastasis in epirubicin-resistant TNBC cells in vivo.

### Targeting PLSCR1 with mogroside IV-A reversed epirubicin resistance in TNBC resistant strains

**Fig. 8:**
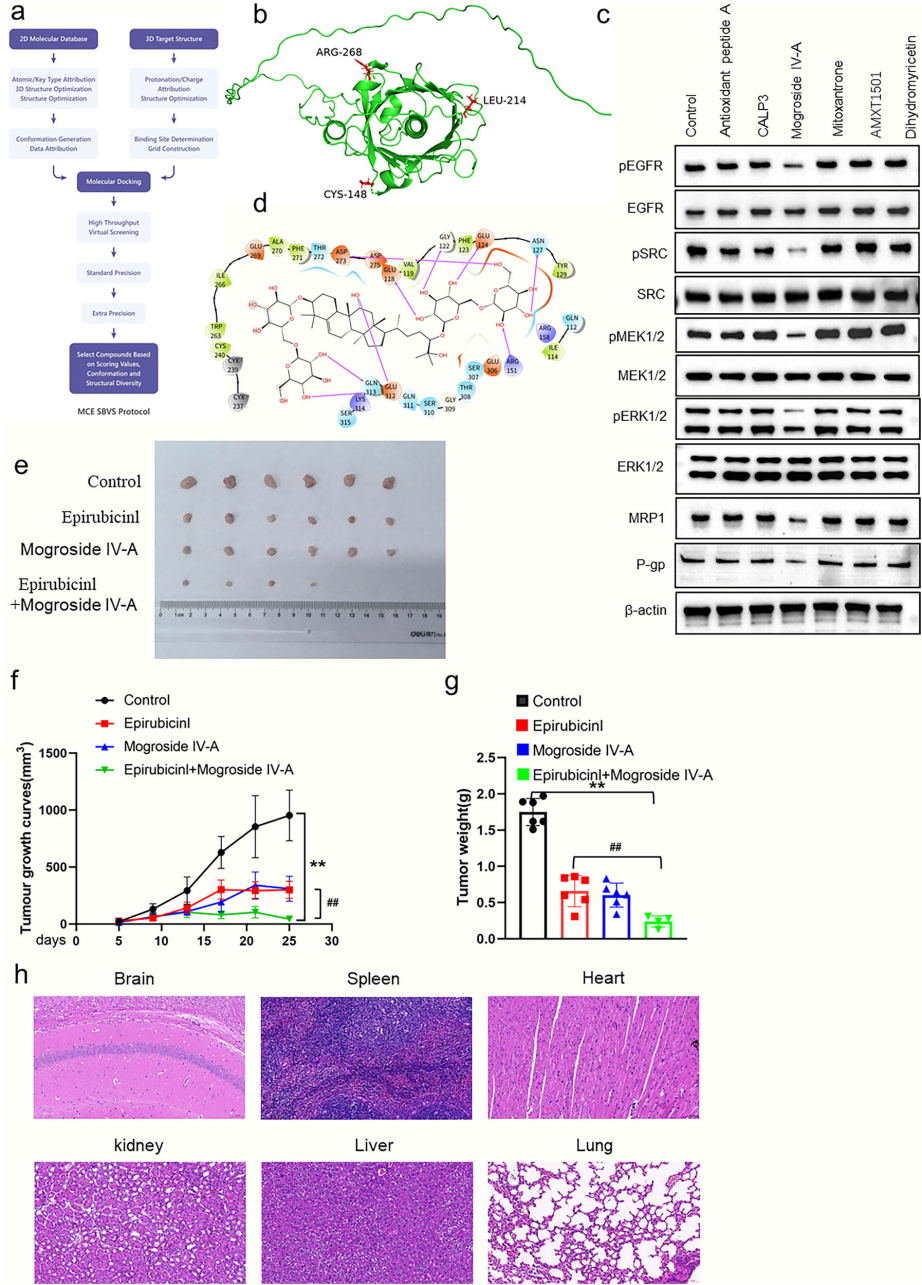
Targeting PLSCR1 with mogroside IV-A reversed epirubicin resistance in TNBC resistant strains. **a** Schematic workflow of small-molecule compound screening; **b** Predicted three-dimensional structure of PLSCR1 showing the putative ligand-binding pocket; **c** Identification of small-molecule inhibitors of PLSCR1 by Western blot analysis; **d** Schematic diagram of Mogroside IV-A binding to PLSCR1; **e**, **f** The proliferation curve of subcutaneous tumors in mice treated with epirubicin (*n* = 6/group); **g** The weight of subcutaneous tumors in mice treated with epirubicin (*n* = 6/group); **h** H&E staining of major organs (heart, liver, spleen, lung, kidney, brain) from mice treated with Mogroside IV-A, assessing histopathological changes. Values are mean ± SD *, *P* < 0.05; **, *P* < 0.01.

To address the absence of reported PLSCR1 small molecule inhibitors, we virtually screened the MCE compound library. Molecular docking was subsequently employed to validate the inhibitory potential of the top six candidate compounds identified (Fig. 8a, b and S10a). Through Western blot experiments with six small molecules, we observed that Mogroside IV-A can significantly inhibit PLSCR1-promoted EGFR phosphorylation and its downstream signaling pathways (Fig. 8c). Further investigation via dose-dependent assay revealed that pEGFR showed a decrease in phosphorylation with the increase in Mogroside IV-A dose (Fig. S10b). Beside, The mutation of three key PLSCR1 residues involved in strong hydrogen bonding with Mogroside IV-A (E124D, R151K, Q313N) resulted in the loss of the compound’s ability to rescue EGFR phosphorylation, as demonstrated by Western blot (Fig. 8d and S10c, d). Intriguingly, we found that the interaction between PLSCR1 and EGFR was not affected through GST-pull down assay (Fig. S10e). Based on these results, we considered that Mogroside IV-A may serve as a potential natural inhibitor for targeted PLSCR1, thereby reversing the tolerance of TNBC resistant strains to epirubicin treatment. To further prove this hypothesis, we create a xenograft tumor model by implanting MDA-MB-231-Edr into nude mice. After 1 week of orthotopic injection, mice were randomly divided into three groups: treated with Saline, Epirubicin + Saline or Epirubicin + 20 mg/kg Mogroside IV-A. The results from the tumor growth curves and weights showed that Mogroside IV-A was able to reverse the acquired resistance of TNBC resistant cells to epirubicin in vivo (Fig. 8e–g). To assess the toxicity of Mogroside IV-A, we treated normal mice with the compound at 20 mg/kg and conducted comprehensive pathological and hematological tests It is noteworthy that we did not observe apparent drug toxicity in the hearts, livers, lungs, or kidneys of mice in the Mogroside IV-A treatment group (Fig. 8h). Simultaneously, biochemical indicatorsin blood of mice in the Mogroside IV-A treatment group also showed no abnormalities (Fig. S10f). Together, our date demonstrated that Mogroside IV-A can reverse the tolerance of TNBC resistant strains to epirubicin treatment in vivo at a safety dose.

## Discussion

Epirubicin, a representative anthracycline chemotherapeutic agent, induces DNA double-strand breaks by interacting with topoisomerase II and remains a cornerstone of neoadjuvant chemotherapy for TNBC [41]. However, some TNBC patients develop acquired resistance to epirubicin-based regimens, resulting in markedly reduced 5-year survival rates. In this study, we identify the PLSCR1–EGFR–MAPK regulatory axis as a central driver of epirubicin resistance, systematically dissecting its molecular interactions, signaling pathways, and m6A epigenetic modulation (Fig. 7f).

Beyond the established roles of drug efflux pumps such as P-gp/ABCB1 and MRP1/ABCC1, emerging evidence highlights the interplay between membrane protein dynamics, epigenetic reprogramming, the tumor microenvironment, and cancer stemness in mediating resistance [19, 42–45]. Previously, we found that PLSCR1 enhances the stemness of TNBC cells [10]. Here, we further demonstrate that PLSCR1, as a membrane protein, binds to EGFR and activates the MAPK pathway. The EGFR–MAPK signaling axis is well-known for its role in therapeutic resistance across cancer types, including the regulation of drug efflux pump expression and maintenance of stemness in TNBC [46–48]. Meanwhile, based on our findings (Fig. 1b) that PLSCR1 not only enhances the tolerance of breast cancer cells to chemotherapy drugs, but also its expression positively correlates with resistance to targeted therapies in breast cancer. Regarding this point, we propose a possible mechanistic link hypothesis. Firstly, PLSCR1 can bind to EGFR and enhance its phosphorylation level, which may interfere with the efficacy of many drugs targeting EGFR (eg: Lapatinib). Secondly, by amplifying MAPK signaling, PLSCR1 may directly undermine the efficacy of mTOR inhibitors (eg: Rapamycin). The MAPK and mTOR pathways are intricately linked; ERK can directly phosphorylate and inhibit TSC2, a key negative regulator of mTORC1, thereby hyperactivating mTOR signaling [49]. Consequently, PLSCR1-driven MAPK activation could sustain mTORC1 activity even in the presence of rapamycin analogs, leading to drug tolerance. Our findings thus position PLSCR1 as a master regulator of this multidimensional network.

However, several critical questions remain. The snRNA-seq analysis in this study, derived from a limited two-sample comparison, is inherently exploratory. Given the small cohort size, we deliberately refrained from making extensive claims regarding broader tumor microenvironment remodeling. Instead, these data served primarily as corroborative evidence at the single-cell level, reinforcing the central finding of elevated PLSCR1 expression in a chemotherapy-resistant context and its preliminary association with stemness markers. All major mechanistic conclusions are firmly grounded in subsequent orthogonal functional validations performed in adequate biological replicates. Consequently, understanding how elevated PLSCR1 in tumor cells influences the broader tumor microenvironment, and the potential feedback effects of this crosstalk on TNBC chemoresistance, remains a critical focus and the logical next step in our ongoing research series. Secondly, the structural basis for the PLSCR1–EGFR interaction, and its functional crosstalk with stemness pathways, is not fully understood—key information for the development of targeted therapies. While we have demonstrated that PLSCR1 regulates the expression of the drug efflux protein MRP1 through the MAPK downstream c-Fos/c-Jun complex, the regulatory mechanism for P-gp remains unclear. We propose two plausible explanations. First, and most directly, this discrepancy may stem from fundamental differences in the responsive elements and regulatory logic of the gene promoters. The expression of ABCB1 (encoding P-gp) is likely governed primarily by nuclear receptors (such as the pregnane X receptor, PXR, or the constitutive androstane receptor, CAR) or other transcription factors. Consequently, it may exhibit low dependency on the MAPK-c-Fos/c-Jun signaling axis and thus show no significant response to the individual knockdown of c-Fos or c-Jun. Second, functional redundancy and compensatory mechanisms within the transcription factor network could be at play. When either c-Fos or c-Jun is knocked down individually, remaining Jun family members (e.g., JunB, JunD) may still form homodimers or partner with other factors, thereby sustaining basal regulatory input to the ABCB1 promoter and masking observable phenotypic changes. In the future, we will conduct in-depth research on these possible causes. Furthermore, HER3, as a member of the EGFR family of receptor tyrosine kinases, shares some of the same protein domains with EGFR. It has been previously demonstrated that HER3 does form heterodimers with EGFR which activates PI3K-AKT pathway in TNBC [50]. So, our next step will be to further investigate the regulatory relationship between PLSCR1 and HER3, which will be of great significance for studying the cross-talk of MAPK and PI3K-AKT pathways in the drug resistance of TNBC. In addition, our single-cell sequencing data indicated a co-expression relationship between PLSCR1 and several breast cancer stemness markers (e.g., ALDH1 and CD44) (Fig. S1f). Further flow cytometry analysis revealed that high PLSCR1 expression in TNBC resistant cells significantly enhanced the fluorescent intensity of both CD44 and ALDH1A1. However, upon treatment with a MAPK pathway inhibitor, the fluorescent intensity of CD44 was markedly reduced, while, unexpectedly, that of ALDH1A1 remained unchanged (Fig. S2a, b). These results indicate that CD44 expression is regulated by PLSCR1 through the MAPK signaling pathway. Conversely, the sustained ALDH1A1 level suggests that PLSCR1 likely employs an alternative, MAPK-independent mechanism to regulate this stemness marker.

While our study identifies PLSCR1 as a novel upstream modulator of the EGFR–MAPK pathway, additional challenges limit comprehensive mechanistic and clinical interpretation. Notably, mass spectrometry also revealed that Filamin A (FLNA) interacts with PLSCR1 (Fig. S4i). Given that FLNA modulates chemosensitivity to docetaxel via MAPK/ERK signaling in TNBC [51], it is possible another pathway may support PLSCR1-driven MAPK activation. If such an alternative pathway exists, EGFR-targeted drug effects might be partially counteracted. Combination therapyusing EGFR-targeted agents (e.g., gefitinib, erlotinib, cetuximab) and chemotherapy is already used clinically in various tumor types. Thus, future work will include evaluating whether inhibition of PLSCR1 can enhance the efficacy of EGFR-targeted drugs in TNBC models. Pathological tissue samples from patients who received neoadjuvant chemotherapy and EGFR inhibitors will be examined for PLSCR1 expression, and these data will be correlated with clinical outcomes.

N6-methyladenosine (m6A), a key component of epigenetic researches, has emerged as a key regulator of cancer chemoresistance by dynamically modulating mRNA stability, splicing, and translation [52]. Among the major players in this epitranscriptomic landscape, the methyltransferase METTL3 and the RNA-binding protein IGF2BP3 have been increasingly implicated in therapeutic resistance across a variety of malignancies [53, 54]. In this study, we demonstrate that METTL3 is essential for the m6A modification of PLSCR1 mRNA, while IGF2BP3, whose expression is notably upregulated in resistant TNBC cells, recognizes these modifications and stabilizes PLSCR1 mRNA. Interestingly, METTL3 levels did not change dramatically in resistant versus parental cells (Fig. S7e), while IGF2BP3 expression increased significantly in resistant strains (Fig. S7e). These findings suggest that elevated PLSCR1 expression in TNBC resistance is primarily driven by upregulation of IGF2BP3, although METTL3 activity remains necessary for this process. However, the reason for the upregulation of IGF2BP3 in drug-resistant TBNC has not yet been discovered; this is likely due to a coordinated, multi-tiered regulatory network. At the epigenetic level, DNA hypomethylation of its promoter may relieve basal repression [55], while the acquisition or activation of a super-enhancer at the IGF2BP3 locus, marked by dense H3K27ac and BRD4 enrichment, provides a powerful transcriptional drive [56]. Additionally, Ge et al. found that c-Myc directly binds the IGF2BP3 promoter to enhance its transcription. In turn, the elevated IGF2BP3 protein stabilizes c-Myc mRNA and interferes with c-Myc protein degradation, creating a self-reinforcing circuit that amplifies and sustains the expression of both oncogenes [57]. This finding aroused our interest. We treated the drug-resistant TNBC cell lines with MAPK pathway inhibitor PD98059 (Selleck, #S1177), followed by qPCR and Western blot to detect the expression of IGF2BP3. Regrettably, no significant reduction in its expression was observed, indicating that the high expression of IGF2BP3 in drug-resistant TNBC cell lines is not affected by the MAPK pathway (Data not shown). Furthermore, in neuroblastoma, the oncogenic transcription factor MYCN can directly bind to the promoter of IGF2BP3 and promote its transcription; in turn, IGF2BP3 can stabilize MYCN mRNA in an m6A-dependent manner, thereby forming a positive feedback loop that synergistically drives tumor proliferation [58]. In summary, we propose that the upregulation of IGF2BP3 in TNBC chemoresistance is not attributed to a single factor, but rather the combined effects of epigenetic activation, self-amplification of the core transcriptional loop, and potential fine-tuning of the immune microenvironment and signaling pathways. These mechanisms intertwine with one another, such that once triggered, the expression of IGF2BP3 is stably maintained; ultimately, it drives multidrug resistance by stabilizing downstream targets including PLSCR1. The mechanisms underlying the upregulation of IGF2BP3 in TNBC chemoresistance remain largely unexplored, which constitutes a key direction for our follow-up research.

In the context of drug discovery, small-molecule inhibitors generally present greater feasibility than siRNA-based approaches. A primary advantage lies in their superior drug-like properties, including oral bioavailability, efficient cell membrane penetration, and predictable pharmacokinetics, which are essential for systemic administration. In this study, we have identified Mogroside IV-A as a small molecule inhibitor of PLSCR1, which cuts down PLSCR1-EGFR-MAPK-MRP1/P-gp signaling axis. Animal experiments further proved that Mogroside IV-A can effectively and safely reverse the resistance of drug-resistant cells to epirubicin treatment. Overall, these results suggest that targeting PLSCR1 with Mogroside IV-A may be a promising therapeutic strategy to overcome TNBC resistance.

## Supplementary information

Supplementary information

Supplementary material legends

Supplementary Table

IF figures

IHC figures

WB unsliced figures

Protocol for Flow Cytometry Gating Strategy

## Data Availability

The raw sequencing data generated in this study have been deposited in the China National GeneBank Sequence Archive (CNSA) database under the accession number CNP0009102 and CNP0009108. All other relevant data supporting the findings of this study are available within the paper and its Supplementary Information files. Further inquiries can be directed to the corresponding author.
