# Supplementary figures and images for: PLSCR1 drives chemoresistance in TNBC via METTL3/IGF2BP3-mediated mRNA stabilization and EGFR-MAPK pathway activation
Source: Cell Death Dis. 2026 May 15;17(1):624. doi: 10.1038/s41419-026-08845-4 (PMC13347015; doi:10.1038/s41419-026-08845-4)

**Fig. S1.**


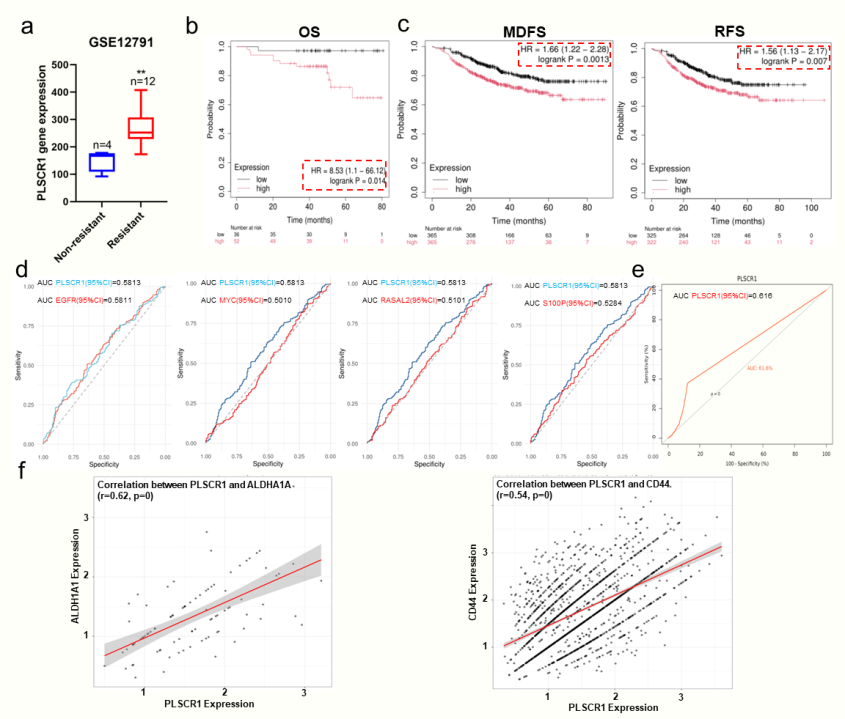


**Fig. S2.**

**
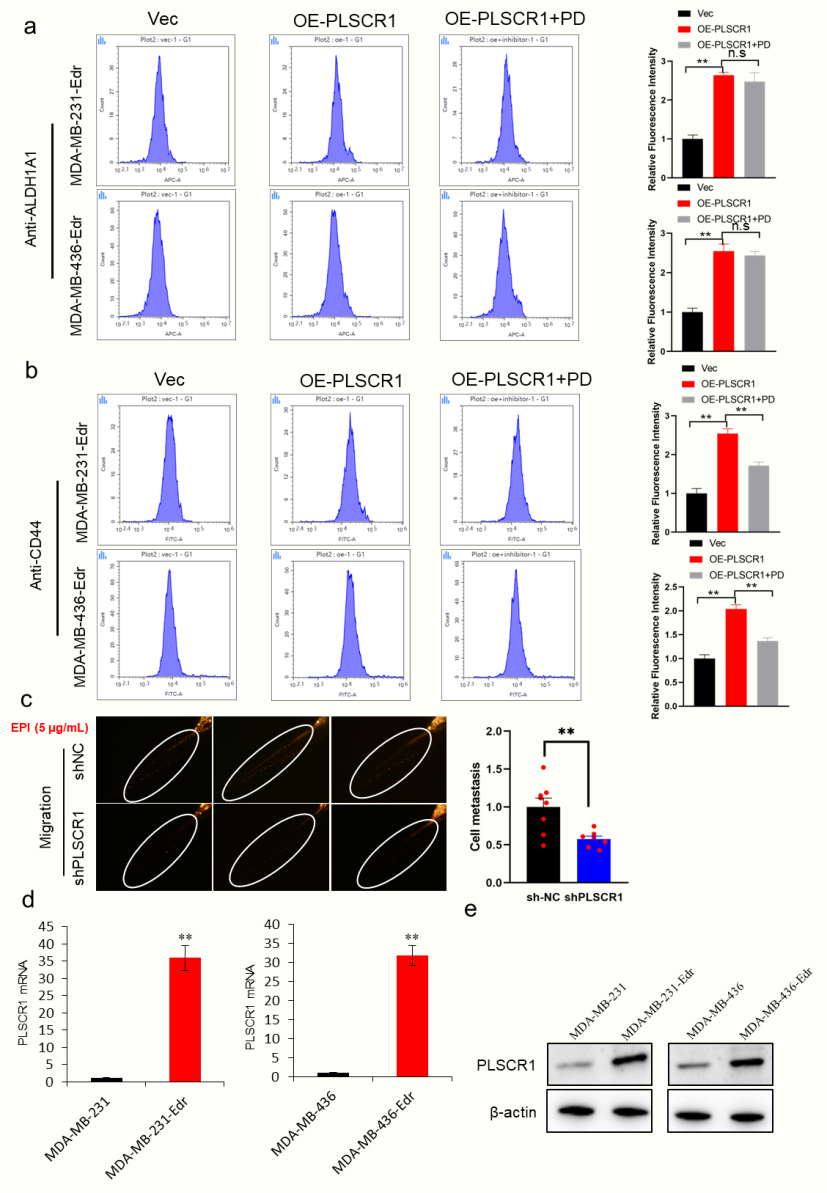
**

**
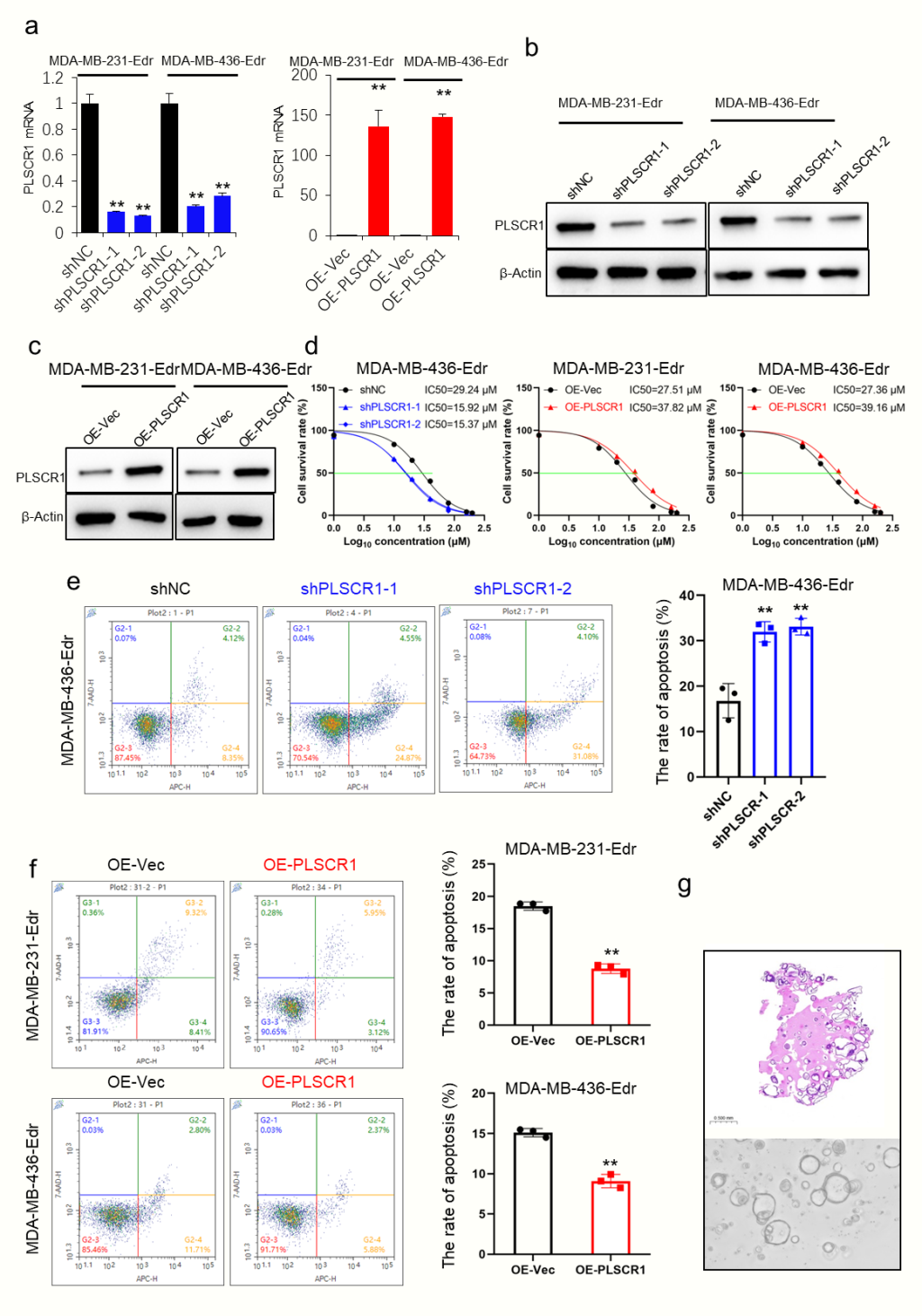
Fig. S3.**


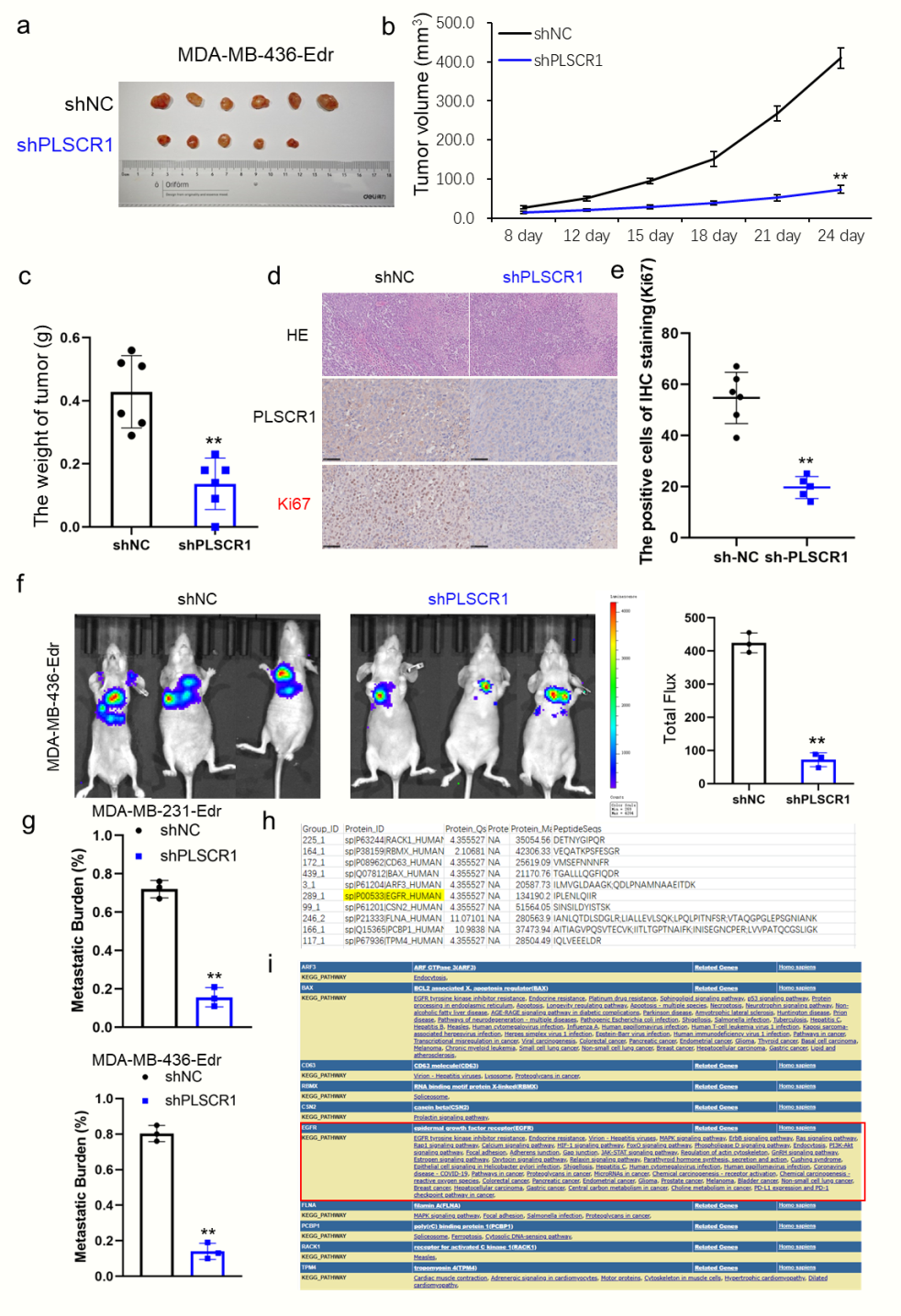
**Fig. S4.**

**Fig. S5.**


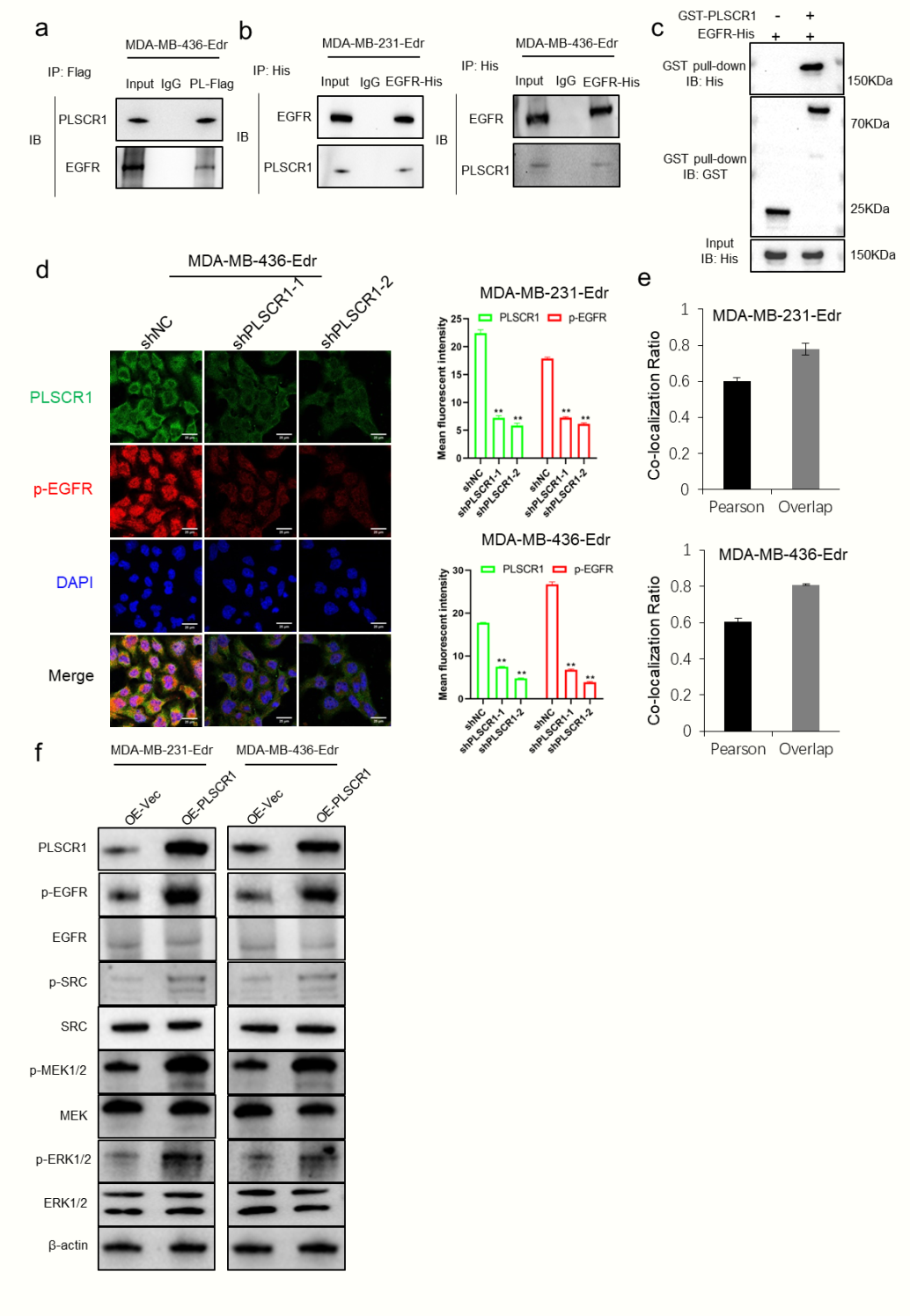


**Fig. S6.**

**
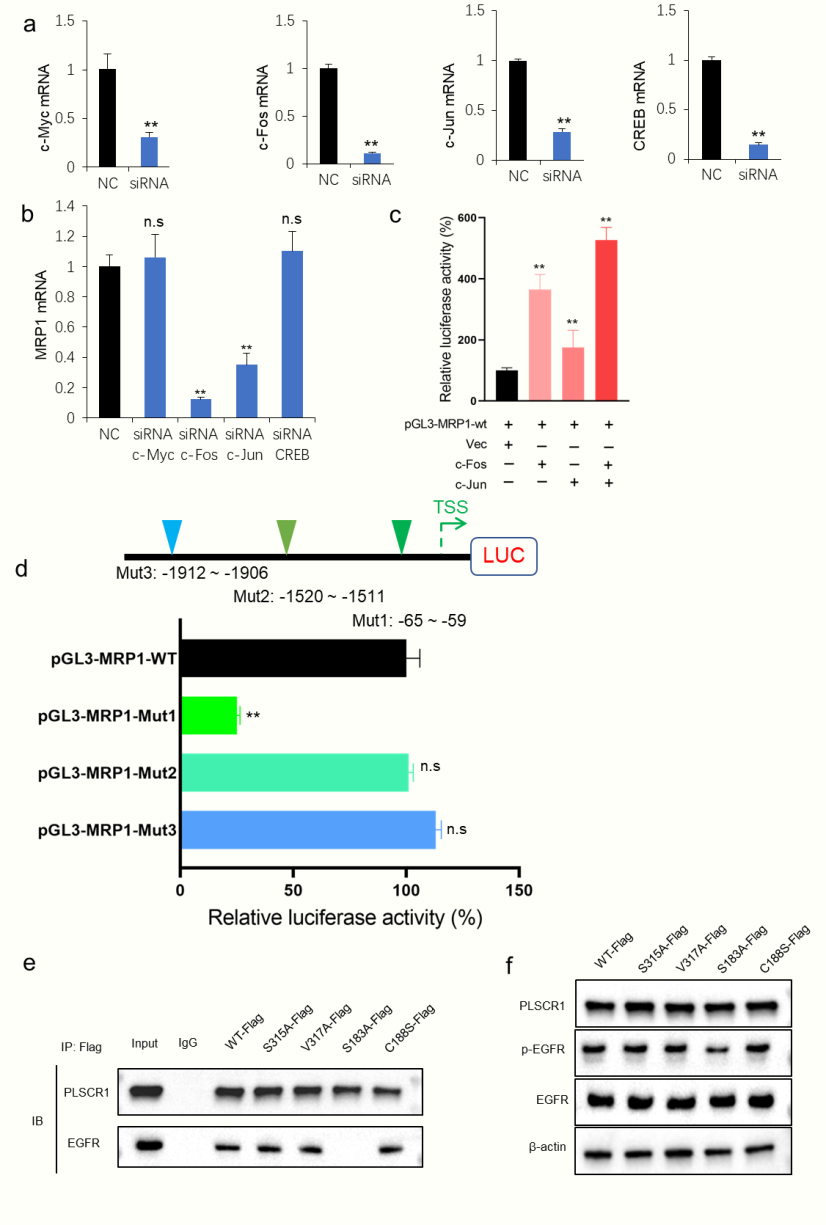
**

**Fig. S7.**


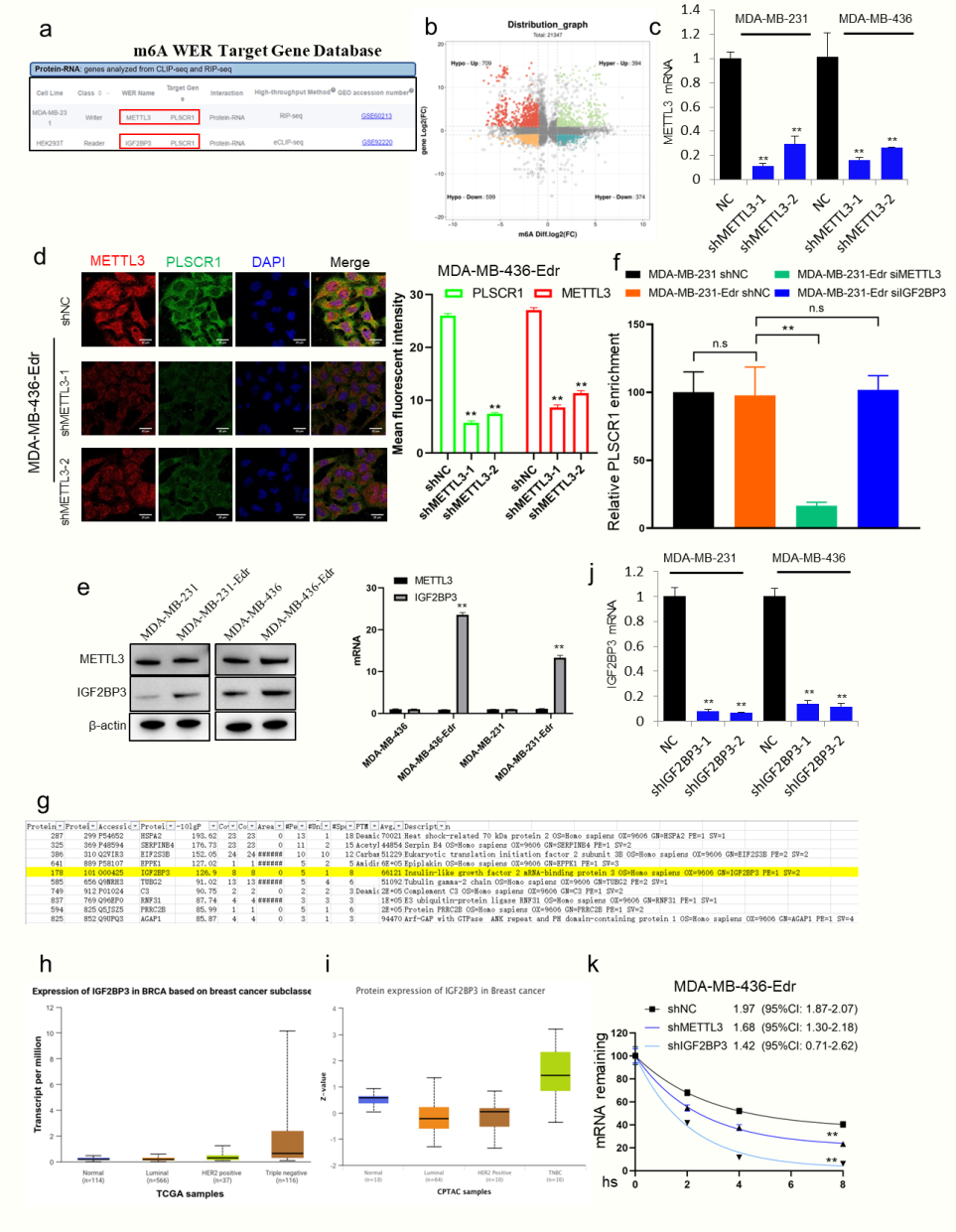


**Fig. S8.**

**
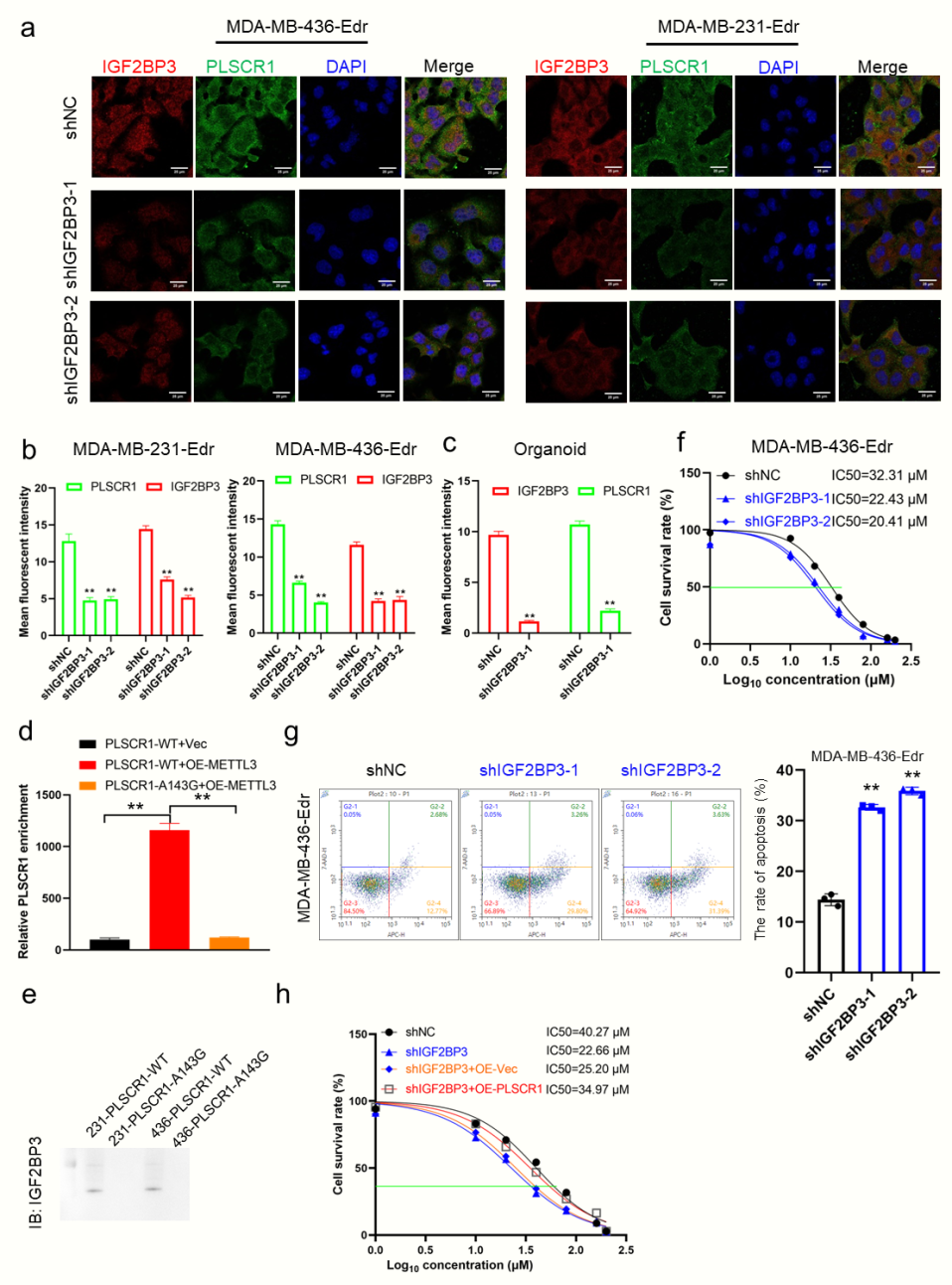
**

**Fig. S9.**


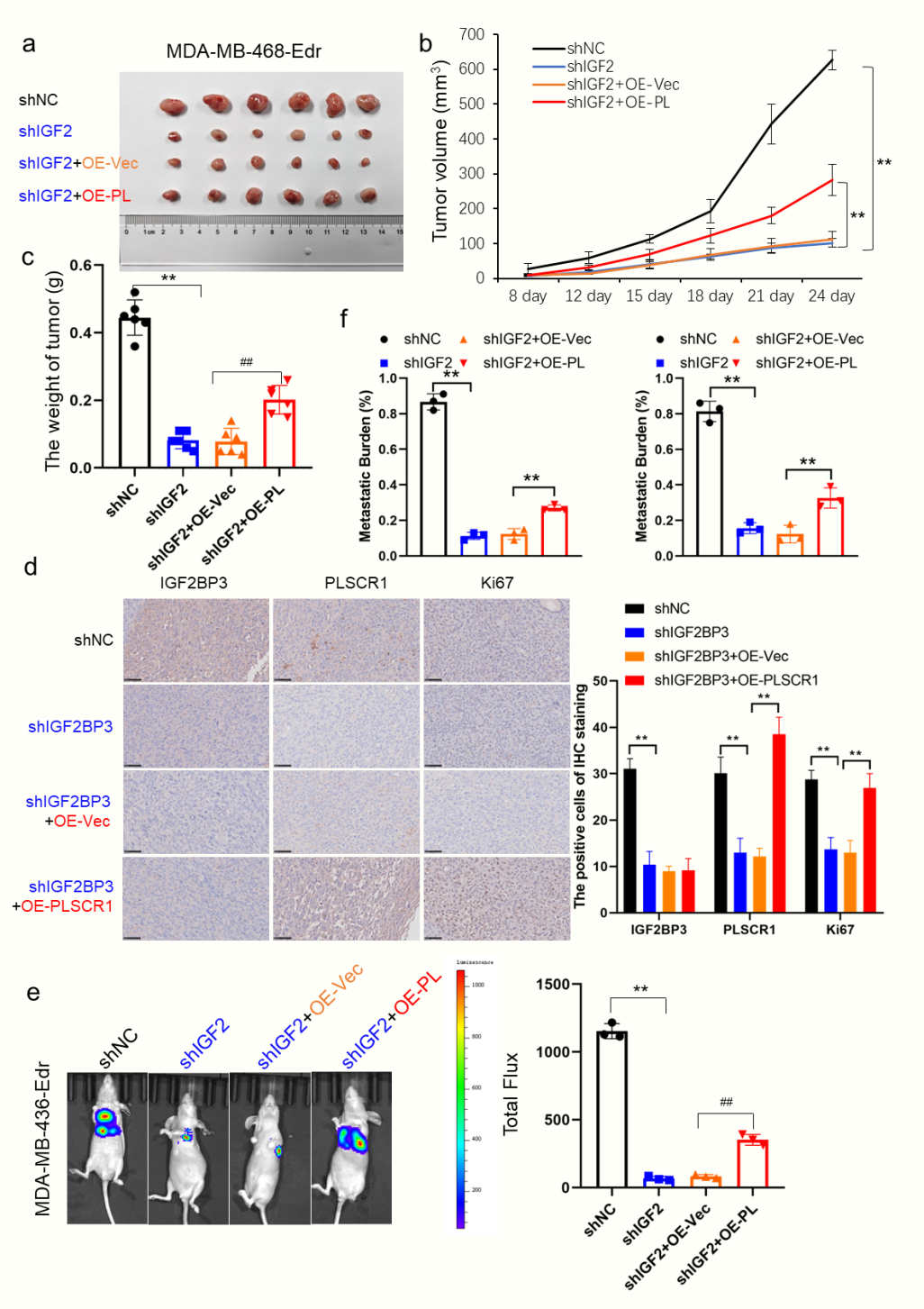


**Fig. S10.**

**
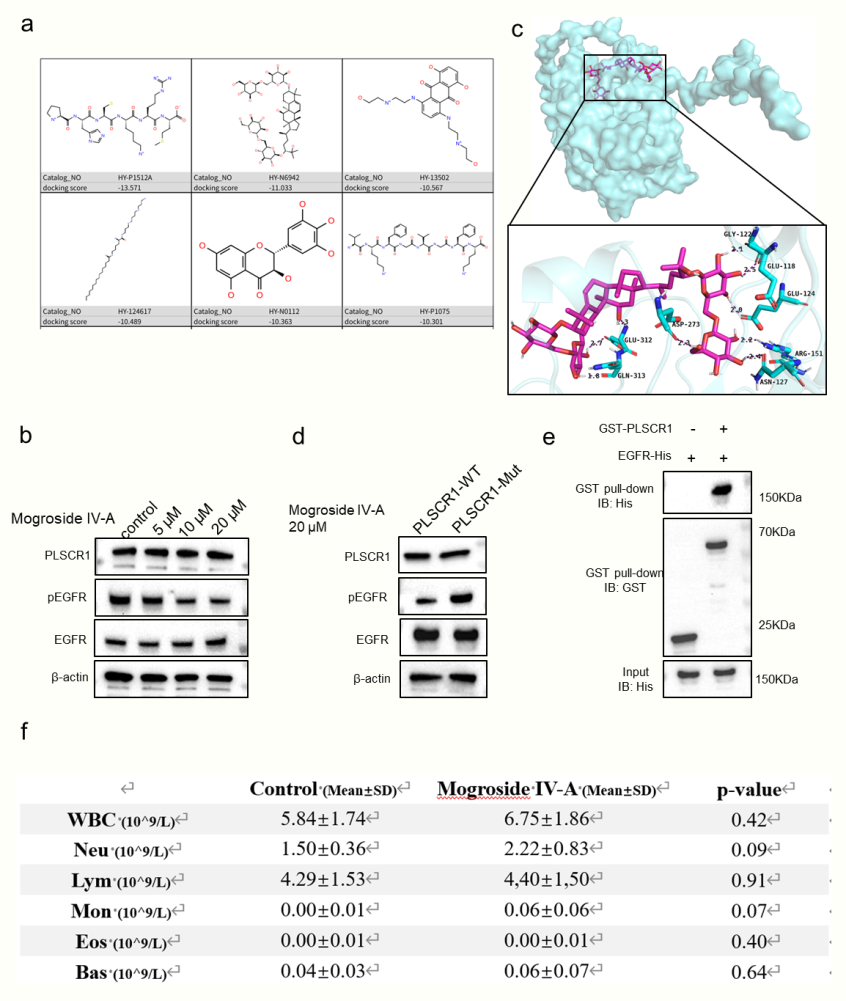
**

Supplement: Supplementary file 1 — Supplementary information [file 41419_2026_8845_MOESM1_ESM.docx]
