# Supplementary material for: PLSCR1 drives chemoresistance in TNBC via METTL3/IGF2BP3-mediated mRNA stabilization and EGFR-MAPK pathway activation
Source: Cell Death Dis. 2026 May 15;17(1):624. doi: 10.1038/s41419-026-08845-4 (PMC13347015; doi:10.1038/s41419-026-08845-4)
Supplement: Supplementary file 2 — Supplementary material legends [file 41419_2026_8845_MOESM2_ESM.docx]

**Fig. S1** **PLSCR1 is correlated with poor prognosis.** (a) Analysis of PLSCR1 RNA level in TNBC cell lines on the basis of the GEO database. n: Sample size; (b) The relation between PLSCR1 and OS after neoadjuvant chemotherapy in TNBC patients; (c) The correlation between PLSCR1 expression and DFS and RFS in BC patients; (d) The diagnostic efficacy of genes for drug-resistant analyses were performed using Rversion 3.5.1; (e) ROC analysis of single-cell data; (f) Correlation analysis of PLSCR1 expression and tumor stemness marker molecules based on single-cell sequencing. Values are mean ± SD *, *P*＜0.05; **, *P*＜0.01.

**Fig. S2 PLSCR1 promotes the stemness of TNBC cells.** (a) Flow cytometric analysis of PLSCR1 and ALDH1A1 in resistant TNBC cells; (b) Flow cytometric analysis of PLSCR1 and CD44 in resistant TNBC cells; (c) The metastasis of tumor cells to the tail of zebrafish was observed by fluorescence microscopy (n=7/group).; (d, e) The expression of PLSCR1 in drug-resistant cell lines was detected by qPCR (d) and Western Blot (e). Values are mean ± SD *, *P*＜0.05; **, *P*＜0.01.

**Fig. S3** **PLSCR1 influences the sensitivity of TNBC cells to epirubicin.** (a) qPCR assays checked the knockdown and overexpression effects of PLSCR1; (b, c) The protein levels of PLSCR1 tested by Western Blot. (d) The IC50 value of epirubicin in MDA-MB-231-Edr or MDA-MB-436-Edr were tested by CCK8 (n=8/group, 436-shNC 95% CI: 25.74 to 33.12 μM, 436-shPLSCR1-1 95% CI:13.92 to 17.98 μM, 436-shPLSCR1-2 95% CI:12.86 to 17.90 μM, 231-OE-Vec 95% CI:22.23 to 33.63 μM, 231-OE-PLSCR1 95% CI:30.92 to 45.84 μM, 436-OE-Vec 95% CI:24.08 to 30.96 μM, 436-OE-PLSCR1 95% CI:34.05 to 44.89 μM); (e, f) Apoptosis of MDA-MB-231-Edr or MDA-MB-436-Edr were detected by flow cytometry (n=3/group); (g) HE staining image (up) and microscopic image (down) of organoids. Values are mean ± SD *, *P*＜0.05; **, *P*＜0.01.

**Fig. S4** **PLSCR1 knockdown increases the sensitivity of MDA-MB-436-Edr to epirubicin in vivo.** (a, b) The proliferation curve of subcutaneous tumors in mice treated with epirubicin (n=6/group); (c) The weight of subcutaneous tumors in mice treated with epirubicin (n=6/group); (d, e) The expressions of PLSCR1 and Ki67 in the subcutaneous tumors of mice were detected by IHC (n=6/group); (f) PLSCR1 knockdown enhances epirubicin to inhibit tumor metastasis in vivo evaluation by live animal imaging technology (n=3/group). (g) The metastatic burden was quantified using ImageJ software by calculating the percentage of the total metastatic area relative to the total lung area in the analyzed fields; (h) The proteins interacting with PLSCR1 were detected by IP-MS assay; (i) KEGG analysis of proteins with potential interactions with PLSCR1. Values are mean ± SD *, *P*＜0.05; **, *P*＜0.01.

**Fig. S5** **PLSCR1 interacts with EGFR to activate the MAPK pathway.** (a, b) The interaction relationship between PLSCR1 and EGFR detected by CoIP; (c) The interaction relationship between PLSCR1 and EGFR detected by GST-pull down; (d) The expression and co-localization relationship between PLSCR1 and pEGFR were tested in MDA-MB-436-Edr by confocal assay; (e) Image J analysis the co-localization relationship between PLSCR1 and pEGFR in MDA-MB-231/436-Edr;

(f) The role of high expression of PLSCR1 in MDA-MB-231/436-Edr on the MAPK pathway. Experiments were performed with three independent biological replicates. Values are mean ± SD. *, *P*＜0.05; **, *P*＜0.01.

**Fig. S6** **The c-Fos/c-Jun transcription factor complex enhances MRP1 transcription.** (a) qPCR validation of siRNA-mediated knockdown of c-Myc, c-Fos, c-Jun, and CREB; (b) qPCR analysis of MRP1 mRNA levels following siRNA-mediated knockdown of c-Fos and c-Jun; (c) Dual-luciferase reporter assay of MRP1 promoter activity; (d) Dual-luciferase reporter assay mapping the transcription factor binding sites within the MRP1 promoter; (e) Identification of the key PLSCR1-EGFR binding sites by CoIP assay; (f) Identification of the key PLSCR1-EGFR binding sites by Western blot. Values are mean ± SD. *, *P*＜0.05; **, *P*＜0.01.

**Fig. S7** **IGF2BP3 promotes the expression of PLSCR1 in** **epirubicin-resistant TNBC cell lines.** (a) The MeRIP-seq datasets from GEO database analysis enriched proteins on PLSCR1 mRNA; (b) Four-quadrant diagram of the differentially expressed genes and differentially m6A-modified genes by MeRIP-seq; (c) The knockdown effect of METTL3 was detected by qPCR; (d) The expression and co-localization relationship between PLSCR1 and METTL3 were tested in MDA-MB-436-Edr by confocal assay (n=3/group); (e) The expression of IGF2BP3 in Edr-resistant cell lines was detected by Western Blot and qPCR; (f) MeRIP-qPCR assays detected the m6A methylation enrichment level of *PLSCR1* in drug-resistant cell lines; (g) RNA pulldwon-MS assays identified m6A reader protein IGF2BP3; (h, i) UALCAN website detected the mRNA (h) and protein (i) expression levels of IGF2BP3 in different subtypes of breast cancer; (j) The knockdown effect of IGF2BP3 was detected by qPCR; (k) mRNA stability experiment. Values are mean ± SD *, *P*＜0.05; **, *P*＜0.01.

**Fig. S8 IGF2BP3 knockdown increases the sensitivity of Edr-resistant cell lines to epirubicin in vitro.** (a, b) The expression and co-localization relationship between PLSCR1 and IGF2BP3 were tested in MDA-MB-231/436-Edr by confocal assay (n=3/group); (c) Statistically analyze the immunofluorescence results of organoids; (d) RNA enrichment assay confirming METTL3 targeting of the A143 site on *PLSCR1* mRNA; (e) The mRNA of PLSCR1 did not bind the IGF2BP3 protein in PLSCR1-A143G cell lines through RNA pulldown-Western Blot assay; (f) The IC50 value of epirubicin in MDA-MB-436-Edr cell lines were tested by CCK8 (n=8/group, 95% CI: 29.76 to 35.03, 17.19 to 28.57, 15.48 to 25.98 μM); (g) Apoptosis of MDA-MB-436-Edr was detected by flow cytometry (n=3/group); (h) The influence of IGF2BP3 on the IC50 of epirubicin in MDA-MB-436-Edr was tested by CCK8 (n=8/group, 95% CI: 31.23 to 51.06, 18.40 to 27.38, 20.35 to 30.70, 28.79 to 42.17 μM). Values are mean ± SD *, *P*＜0.05; **, *P*＜0.01.

**Fig. S9** **IGF2BP3 enhances the sensitivity of TNBC-resistant strains to epirubicin in vivo.**

(a, b) The proliferation curve of subcutaneous tumors in mice treated with epirubicin (n=6/group); (c) The weight of subcutaneous tumors in mice treated with epirubicin (n=6/group); (d) The expressions of IGF2BP3 and Ki67 in the subcutaneous tumors of mice were detected by IHC (n=6/group); (e) IGF2BP3 knockdown enhances epirubicin to inhibit tumor metastasis in vivo evaluation by live animal imaging technology (n=3/group); (f) The metastatic burden was quantified using ImageJ software by calculating the percentage of the total metastatic area relative to the total lung area in the analyzed fields. Values are mean ± SD *, *P*＜0.05; **, *P*＜0.01.

**Fig. S10 Mogroside IV-A modulates the PLSCR1-EGFR-MAPK signaling axis.** (a) Chemical structures of candidate compounds; (b) Effect of Mogroside IV-A on PLSCR1-mediated EGFR phosphorylation analyzed by Western blot; (c) Molecular docking model illustrating the predicted binding mode of Mogroside IV-A within the PLSCR1 pocket; (d) Assessment of Mogroside IV-A targeting specificity for PLSCR1 by Western blot; (e) GST pull-down assay evaluating the disruption of PLSCR1-EGFR binding by Mogroside IV-A; (f) Serum biochemical parameters assessing the systemic toxicity of Mogroside IV-A in treated mice. Values are mean ± SD *, *P*＜0.05; **, *P*＜0.01.
