# Supplementary material for: PLSCR1 drives chemoresistance in TNBC via METTL3/IGF2BP3-mediated mRNA stabilization and EGFR-MAPK pathway activation
Source: Cell Death Dis. 2026 May 15;17(1):624. doi: 10.1038/s41419-026-08845-4 (PMC13347015; doi:10.1038/s41419-026-08845-4)
Supplement: Supplementary file 3 — Supplementary Table [file 41419_2026_8845_MOESM3_ESM.docx]

**Supplementary Table 1**

Primers

| Gene | Forward | Reverse |
| --- | --- | --- |
| PLSCR1 | CGCGGATCCATGGACAAACAAAACTCACAGAT | CCGCTCGAGCTACCACACTCCTGATTTTTGTT |
| METTL3 | ATTACCTGACGATCACAGGGC | AGTGGCCAACCACATCTGAG |
| IGF2BP3 | CCTTCCTGGTGAAGACTGGC | TCCCACTGTAAATGAGGCGG |

shRNA Sequences

| Gene | Sequences |
| --- | --- |
| shPLSCR1-1 | TACTGGCTGATTATACACTGG |
| shPLSCR1-2 | ATCAAGGTCTAAAGGGAACTG |
| shMETTL3-1 | ATCAGTGGGCAATGTTAAGGC |
| shMETTL3-2 | AACAATGGATTGTTCCTTGGC |
| shMETTL3-3 | GCCAAGGAACAATCCATTGTT |
| shIGF2BP3-1 | AATTCTGAAGTTCATTCACCG |
| shIGF2BP3-2 | TTATACAGCGTCAATTCCTGC |

**Supplementary Table 2**

| **Number** | **Gender** | **Age** | **Tissue subtypes** | **Nerve invasion** | **depth of invasion** | **Miller-Payne-** | **Tumor size(cm)** | **LNM** |
| --- | --- | --- | --- | --- | --- | --- | --- | --- |
| **1** | F | 36 | TNBC | N | T2 | 2 | 4.5 | 4/10 |
| **2** | F | 34 | TNBC | P | T3 | 1 | 6.5 | 10/18 |
