# Supplementary material for: PLSCR1 drives chemoresistance in TNBC via METTL3/IGF2BP3-mediated mRNA stabilization and EGFR-MAPK pathway activation
Source: Cell Death Dis. 2026 May 15;17(1):624. doi: 10.1038/s41419-026-08845-4 (PMC13347015; doi:10.1038/s41419-026-08845-4)

Figure 3d

MDA-MB-231-Edr

shNC

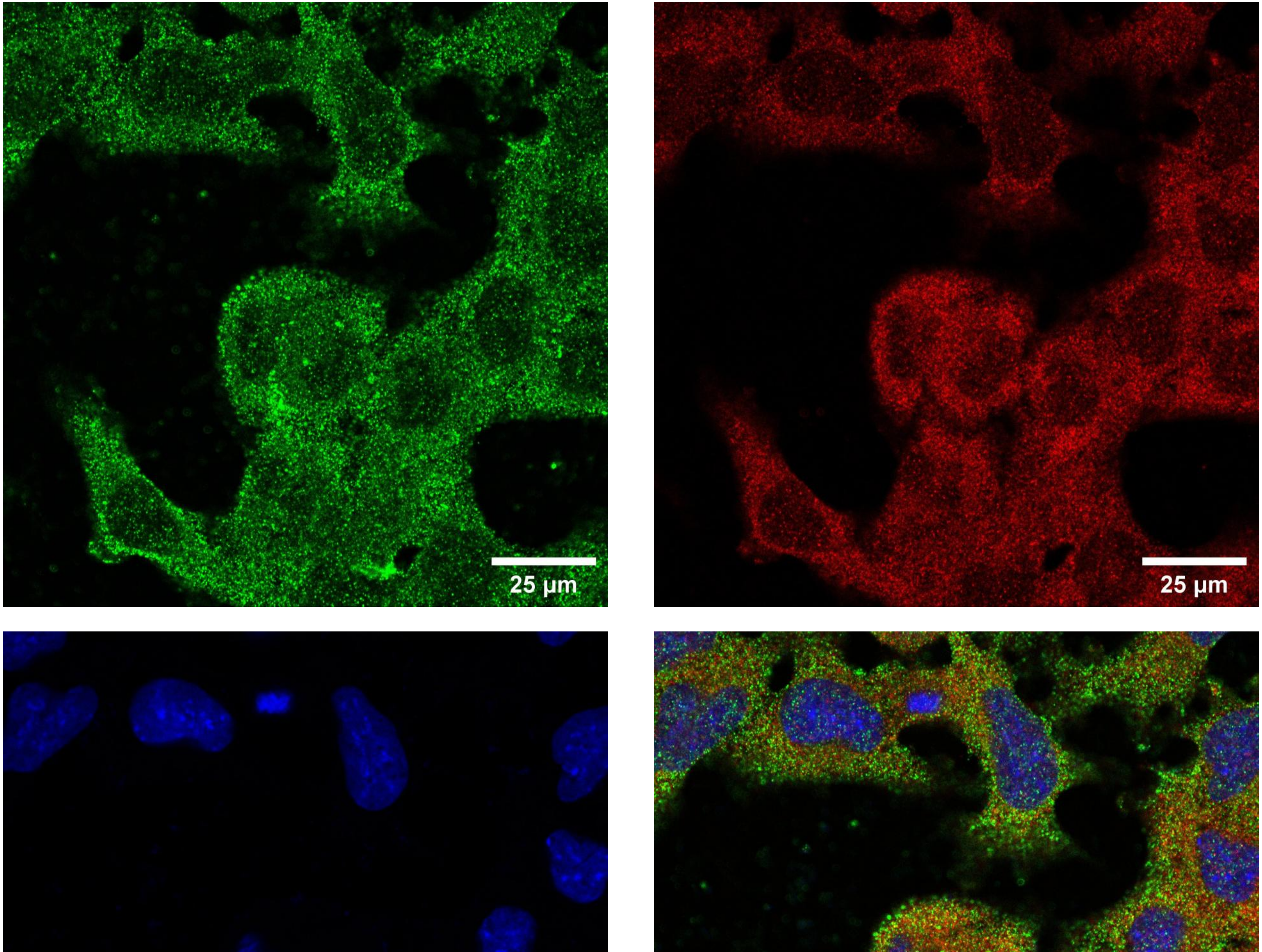

shPLSCR1-1

MDA-MB-231-Edr

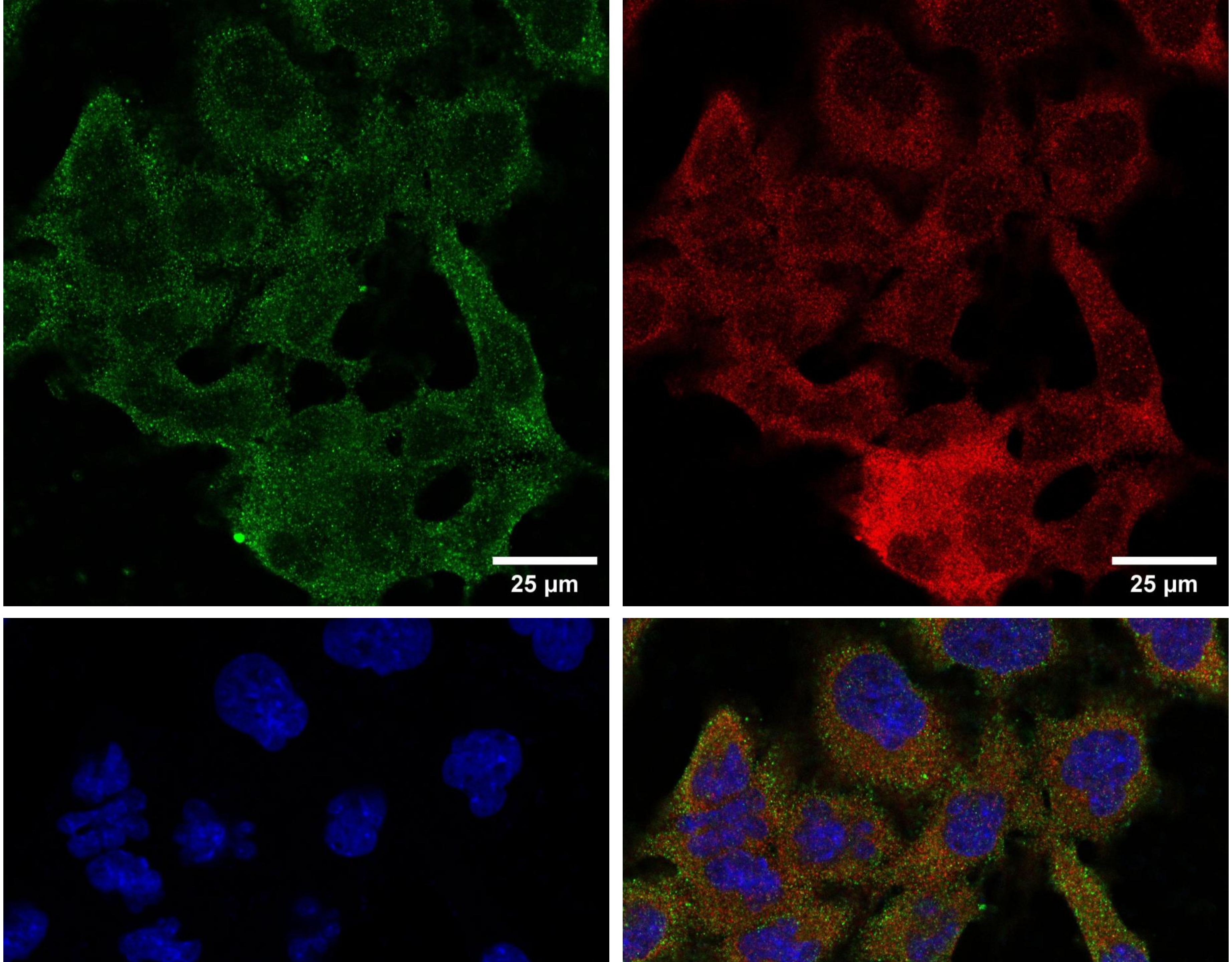

shPLSCR1-2

MDA-MB-231-Edr

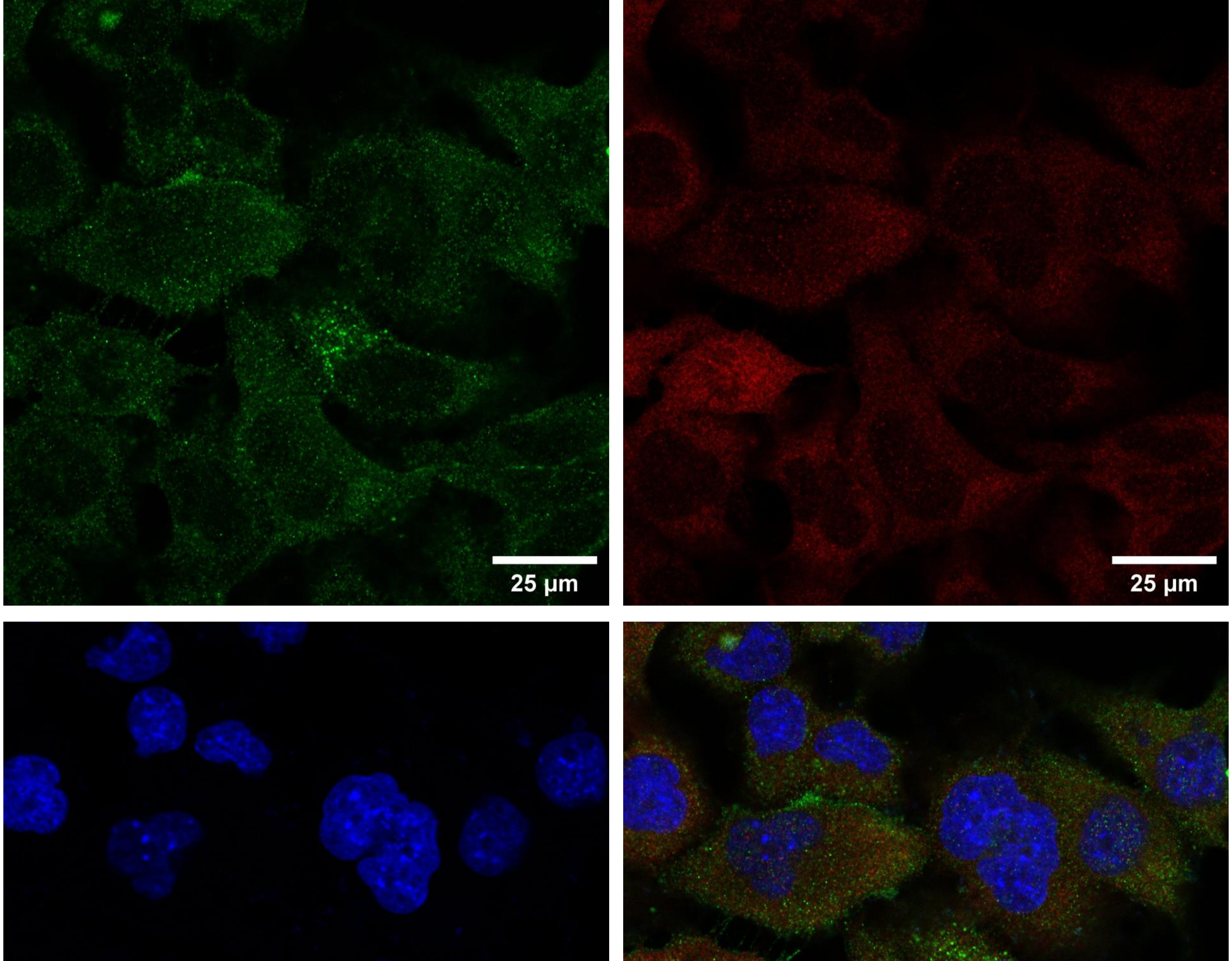

shNC

Organoid

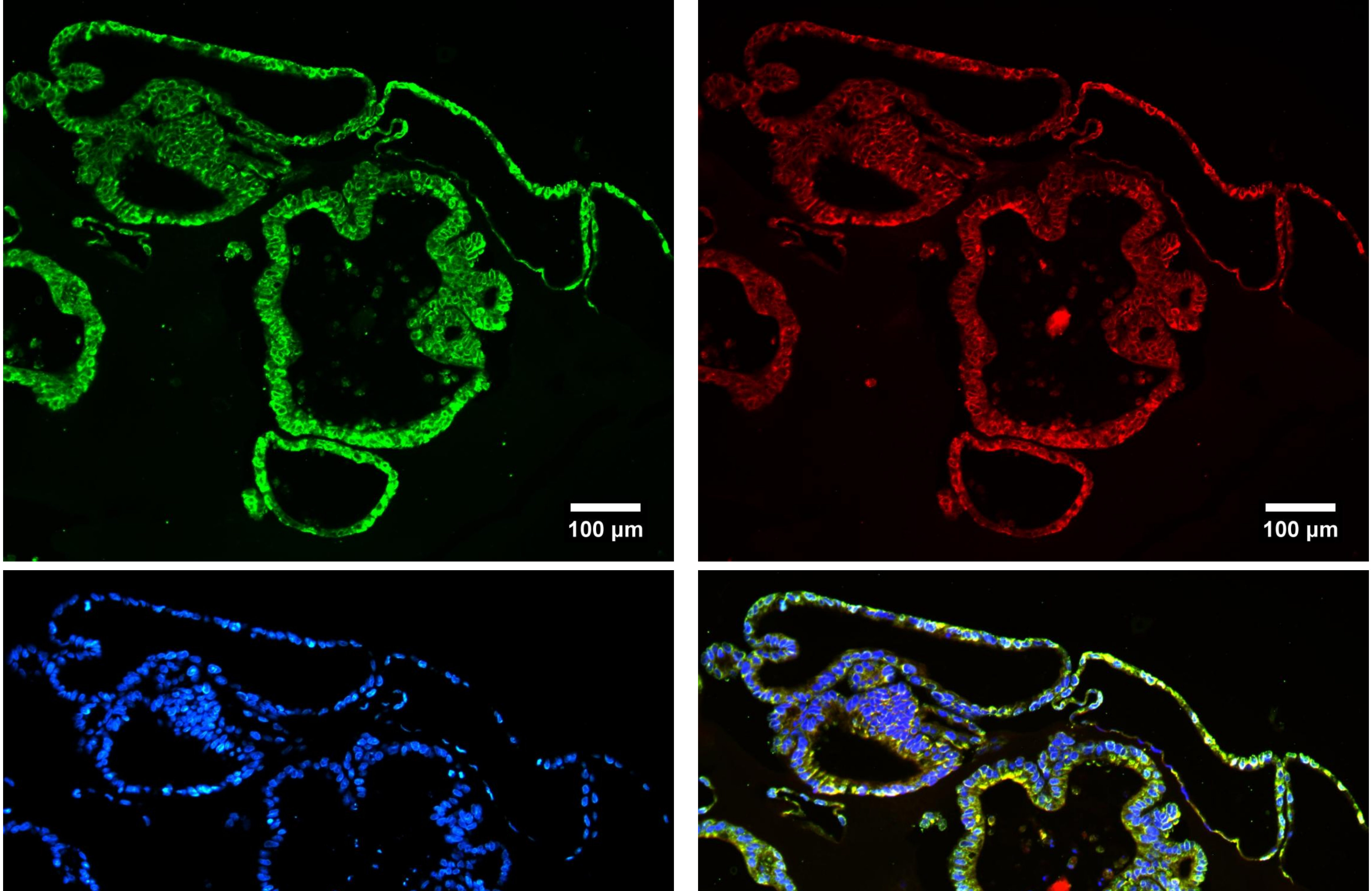

shPLSCR1-1

Organoid

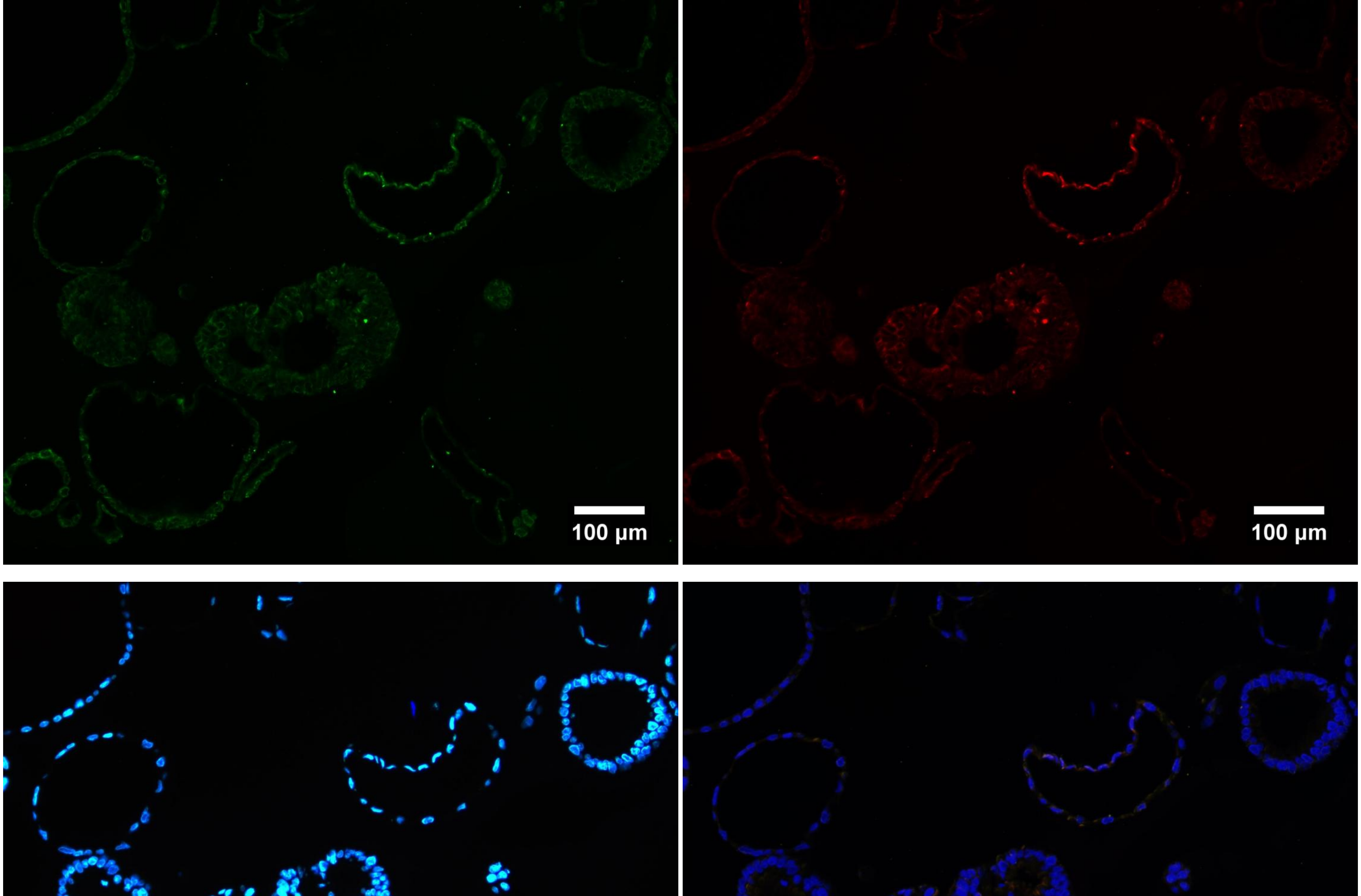

Figure 4f

MDA-MB-231-Edr

shNC

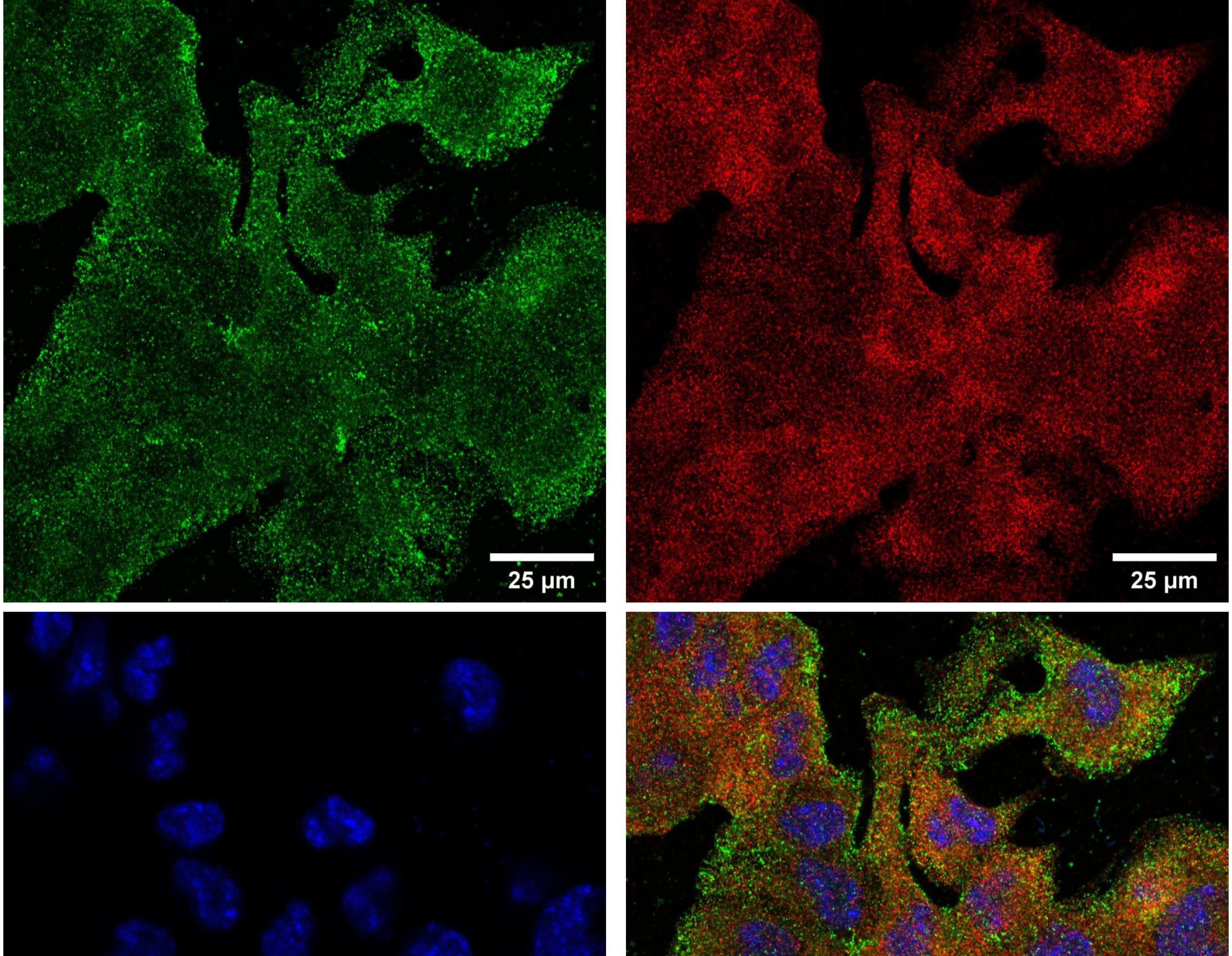

shMETTL3-1

MDA-MB-231-Edr

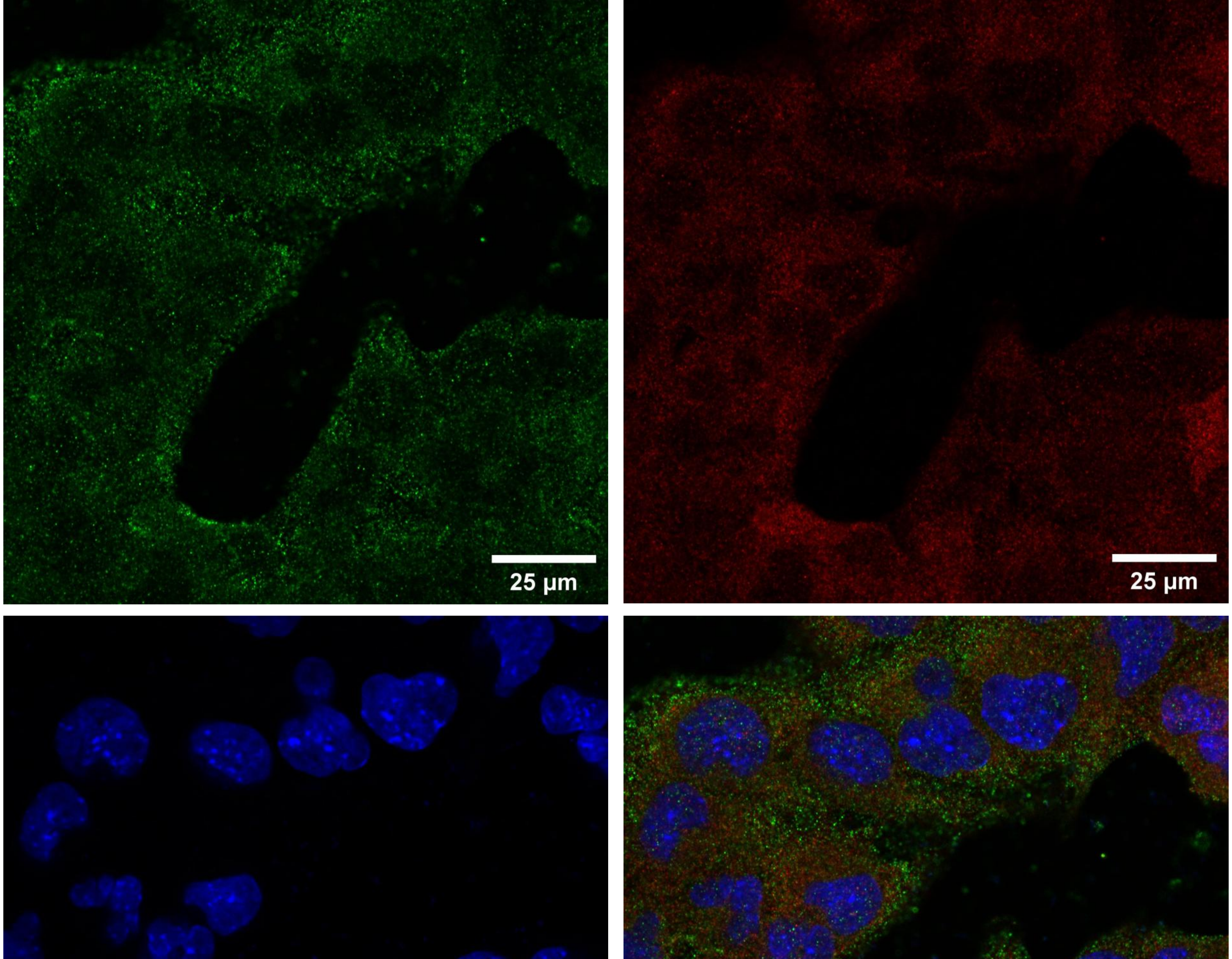

shMETTL3-2

MDA-MB-231-Edr

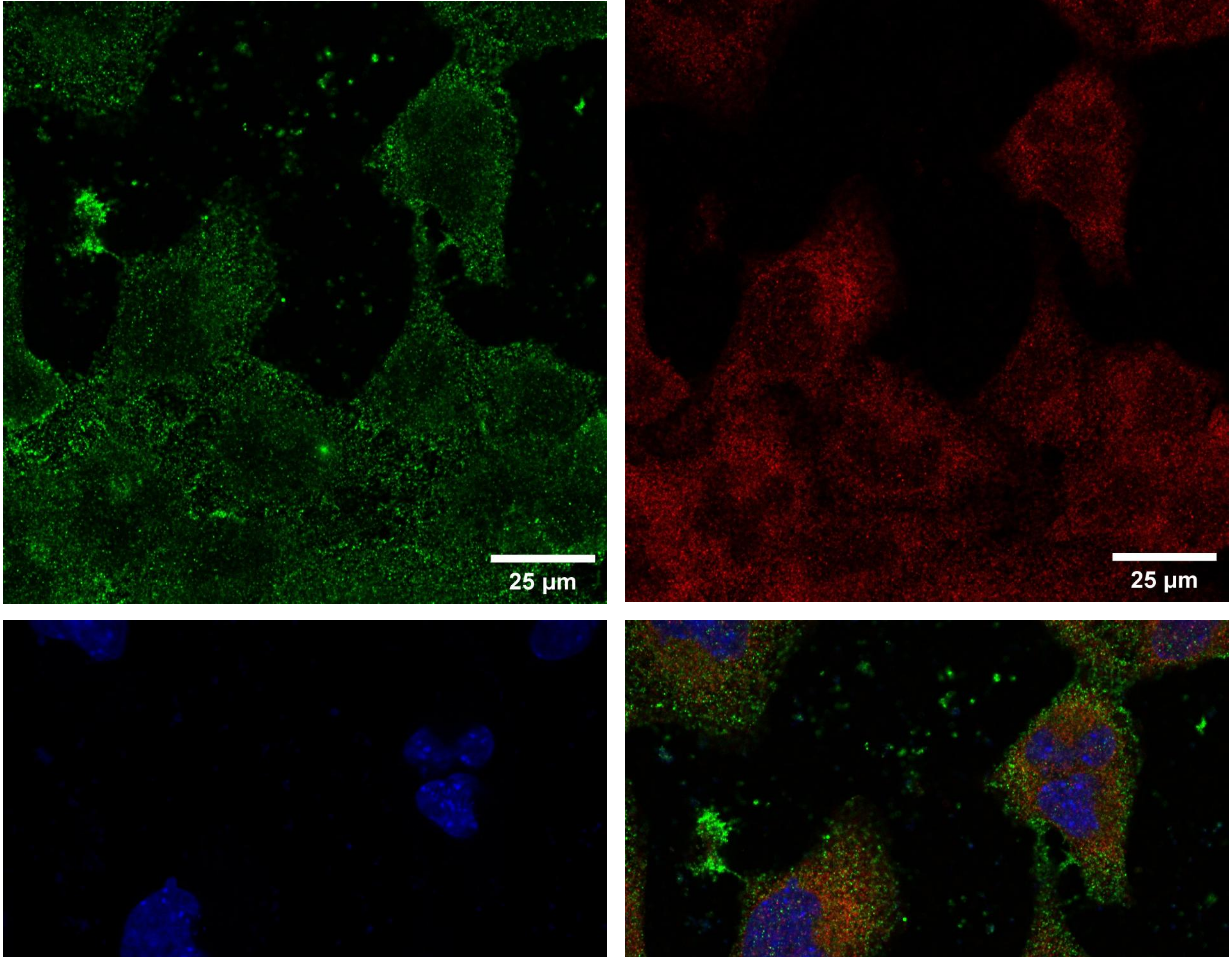

shNC

Organoid

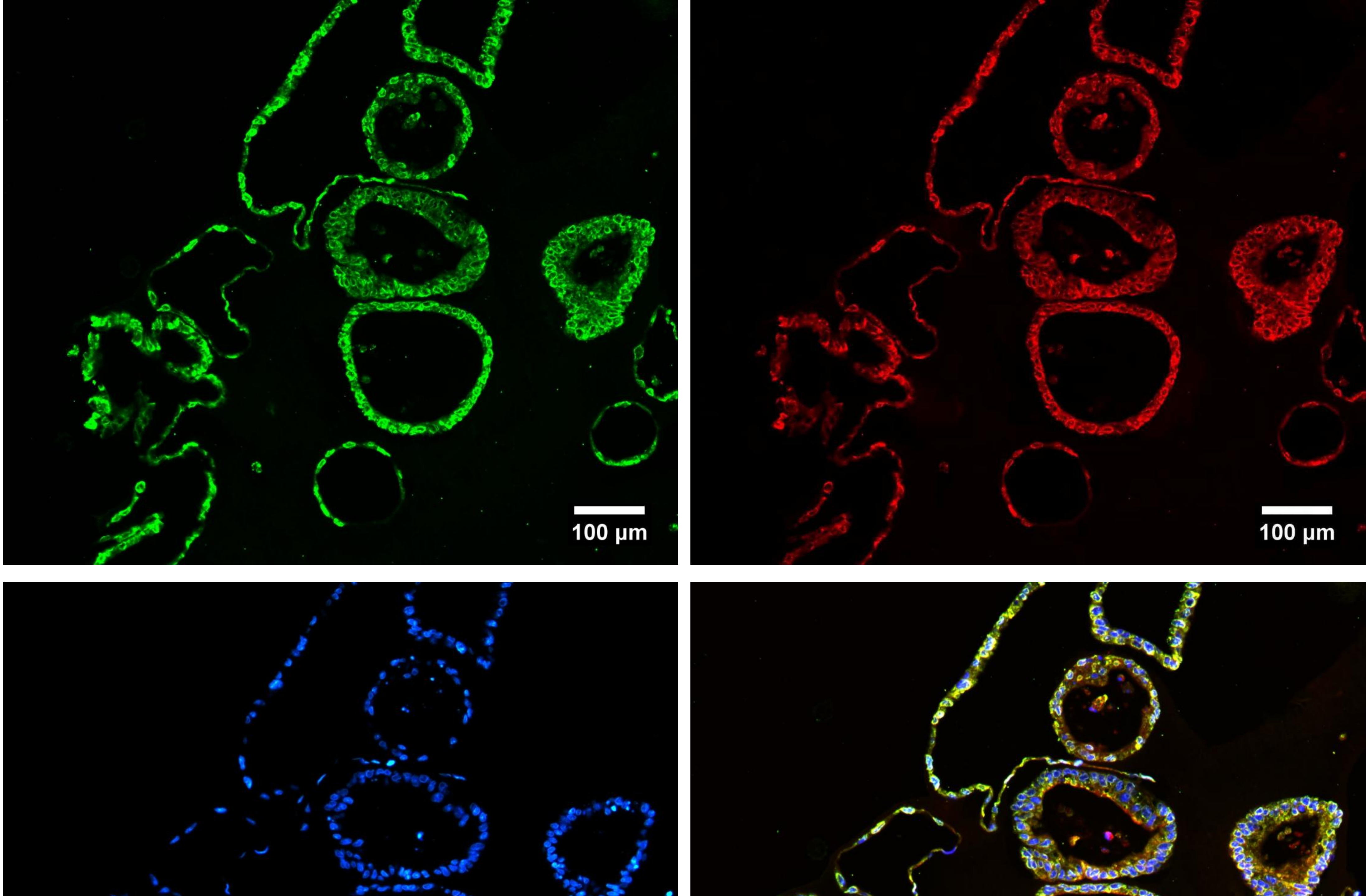

shMETTL3

Organoid

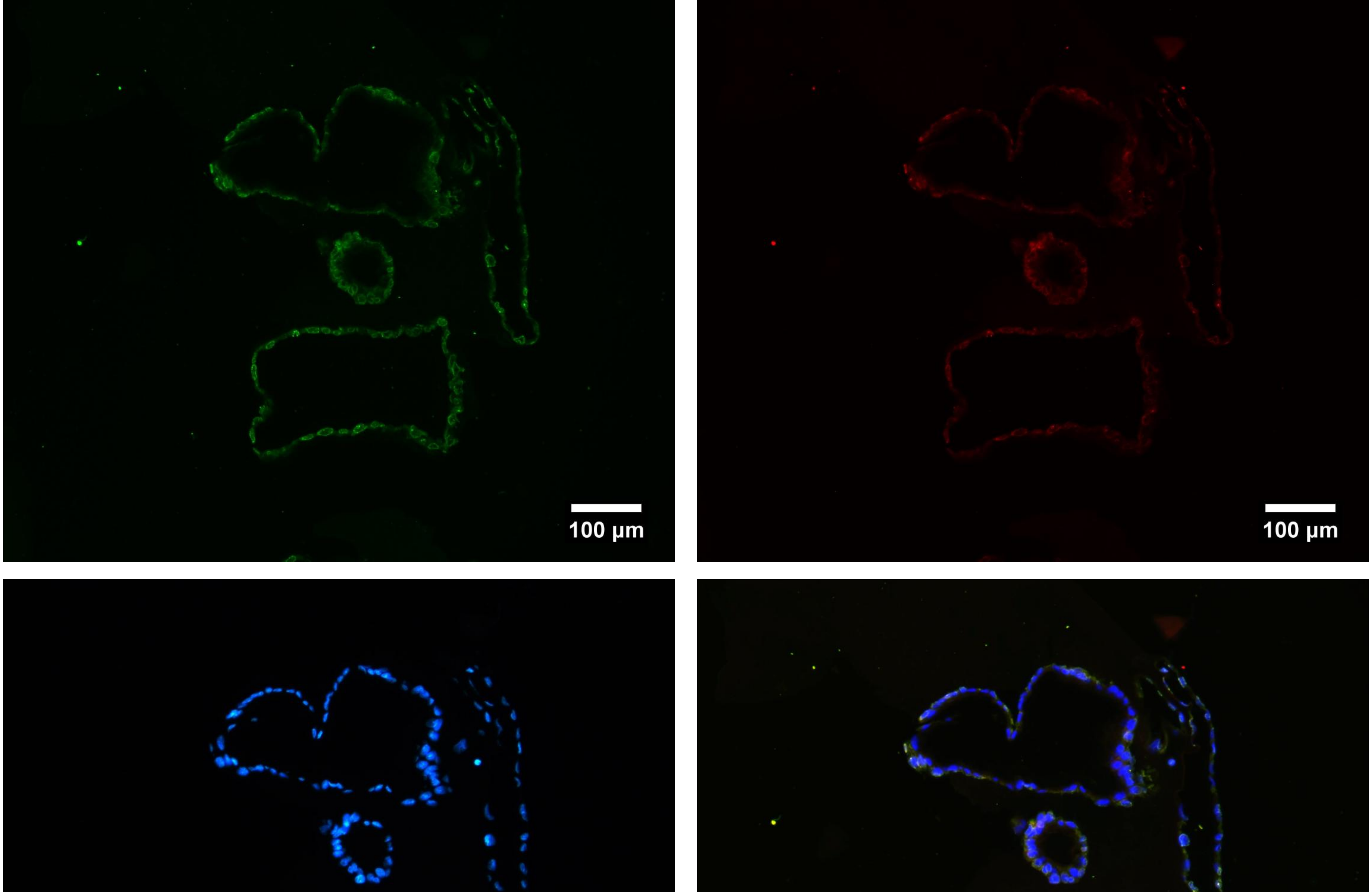

Figure 5e

Organoid

shNC

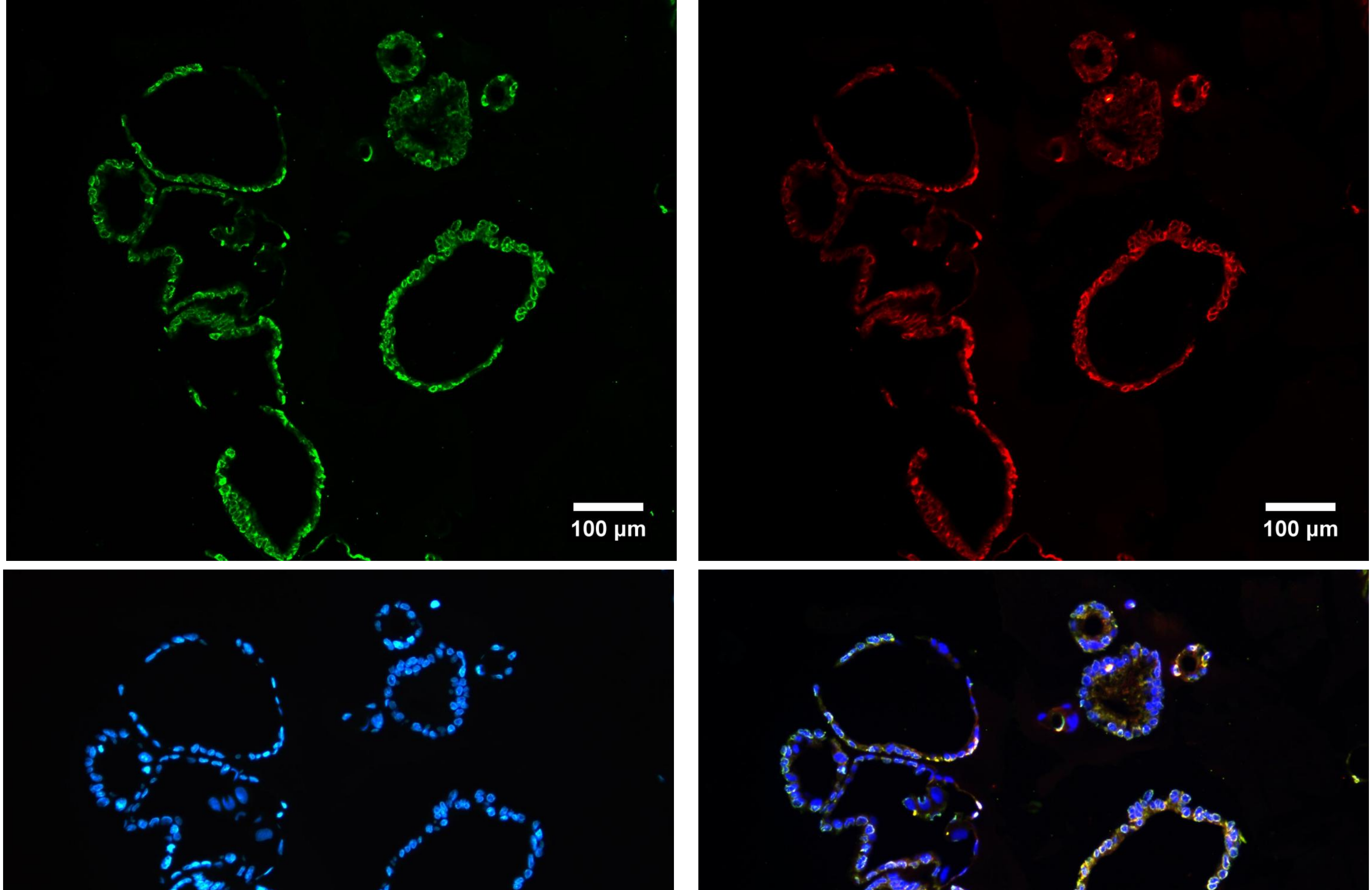

shIGF2

Organoid

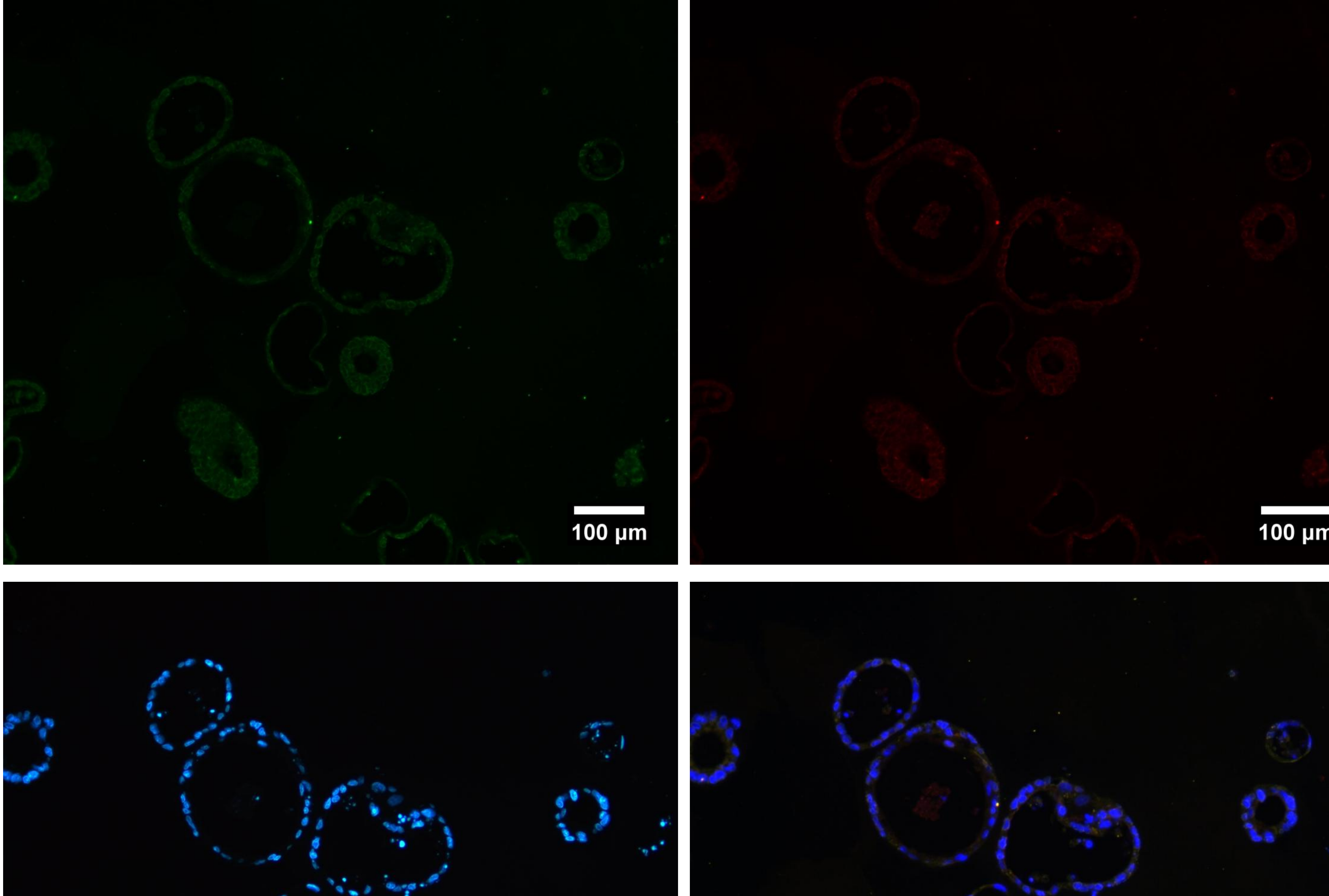

Figure S4d
MDA-MB-436-Edr

shNC

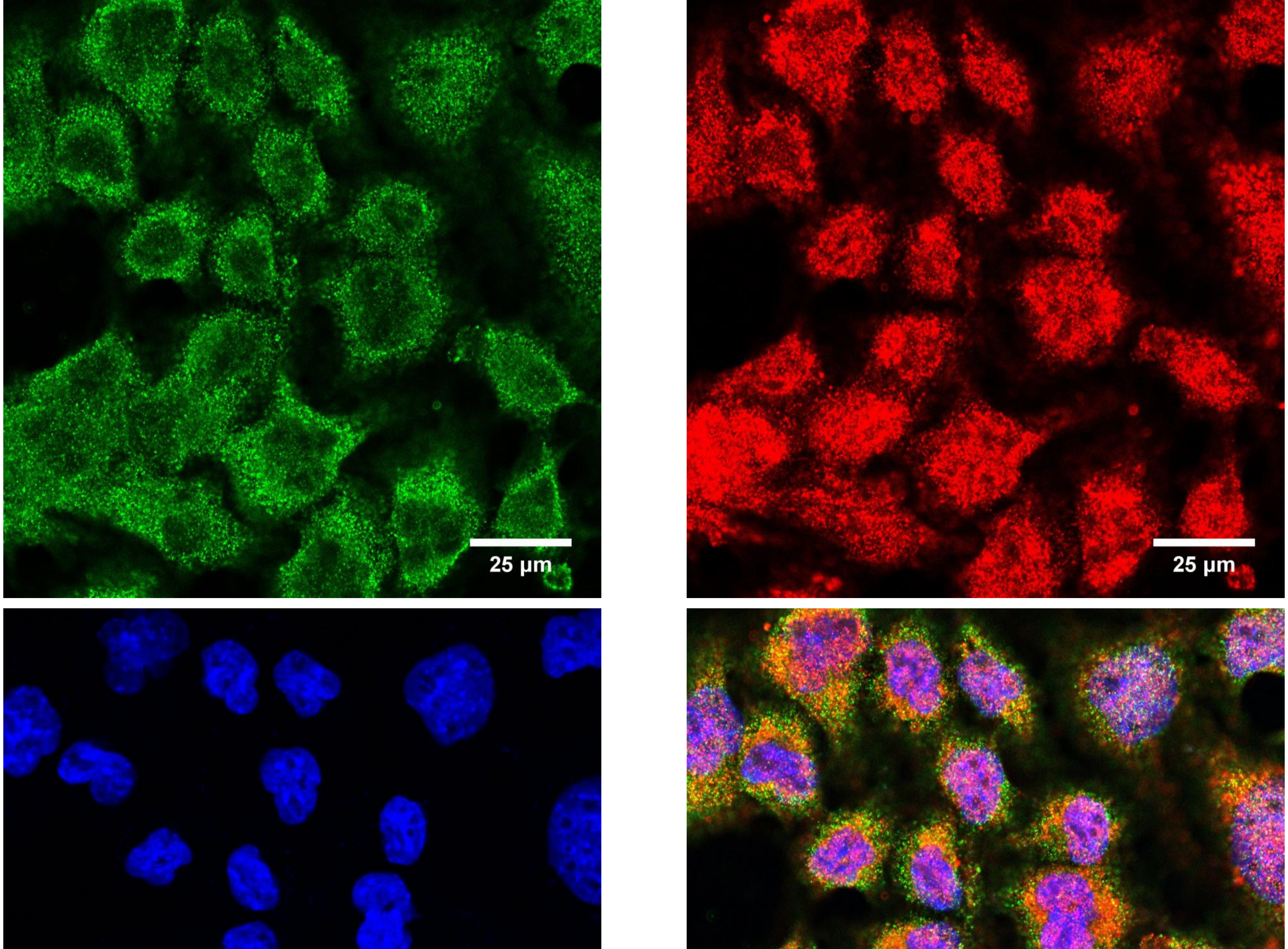

shPLSCR1-1

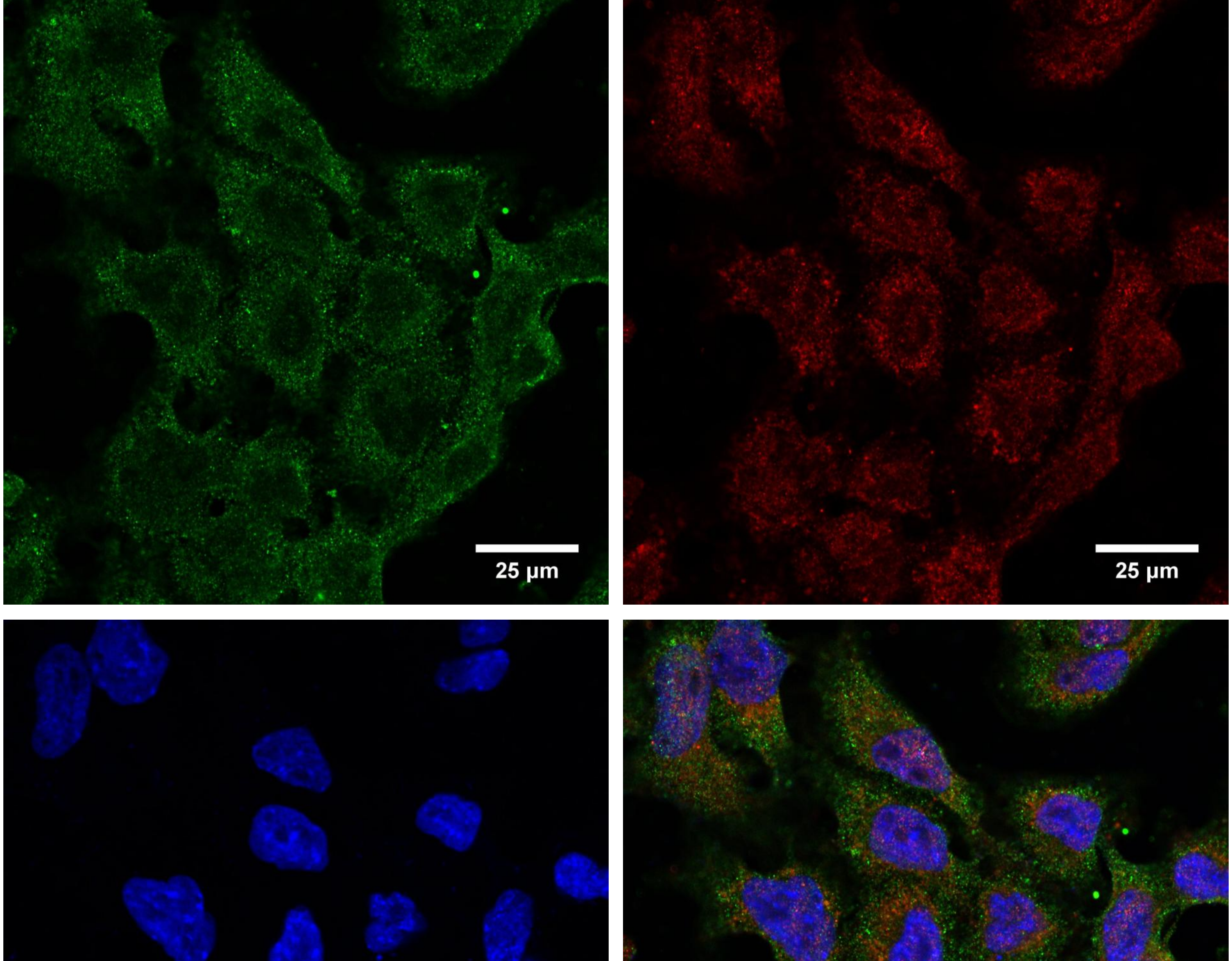

shPLSCR1-2

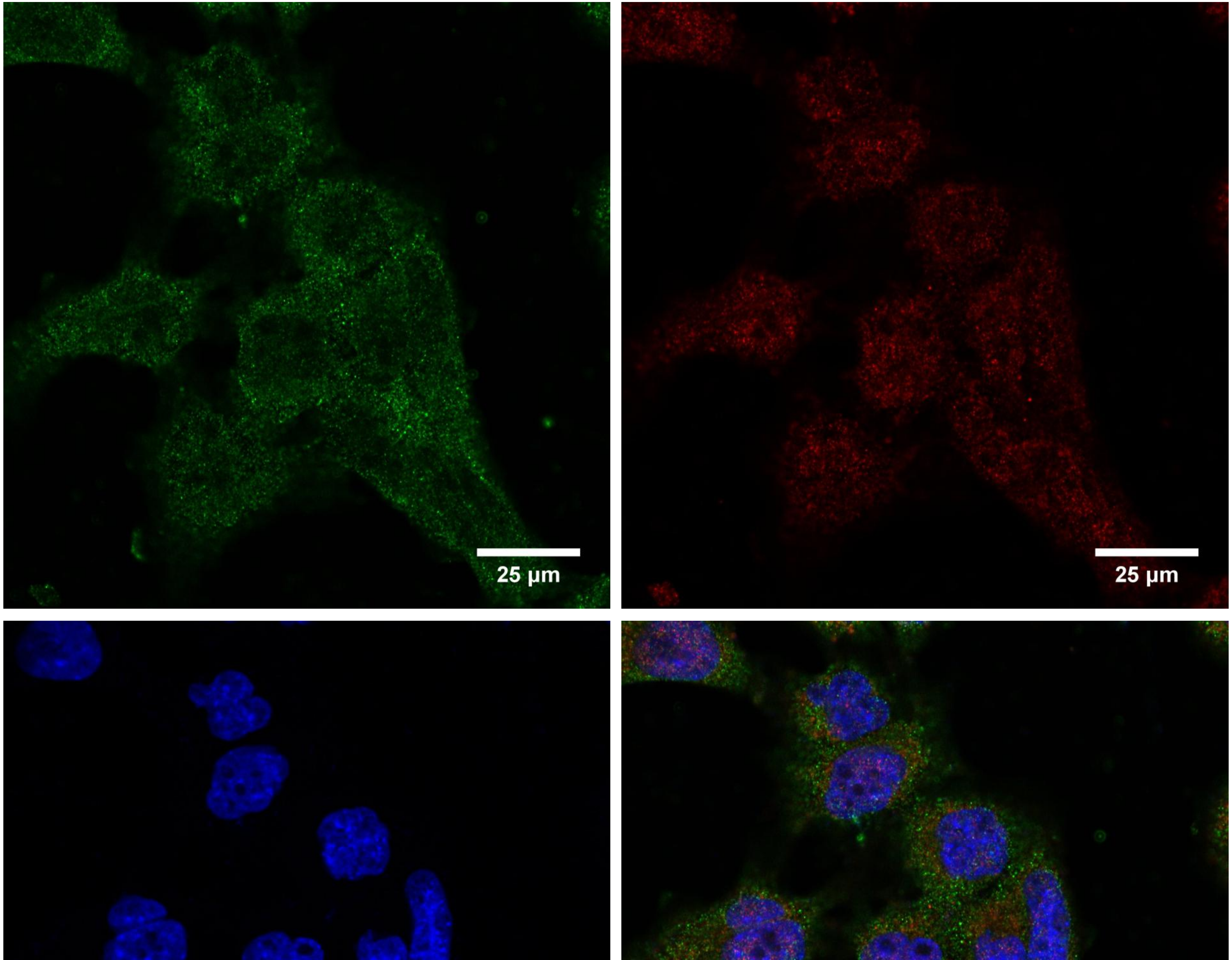

Figure S5

shNC

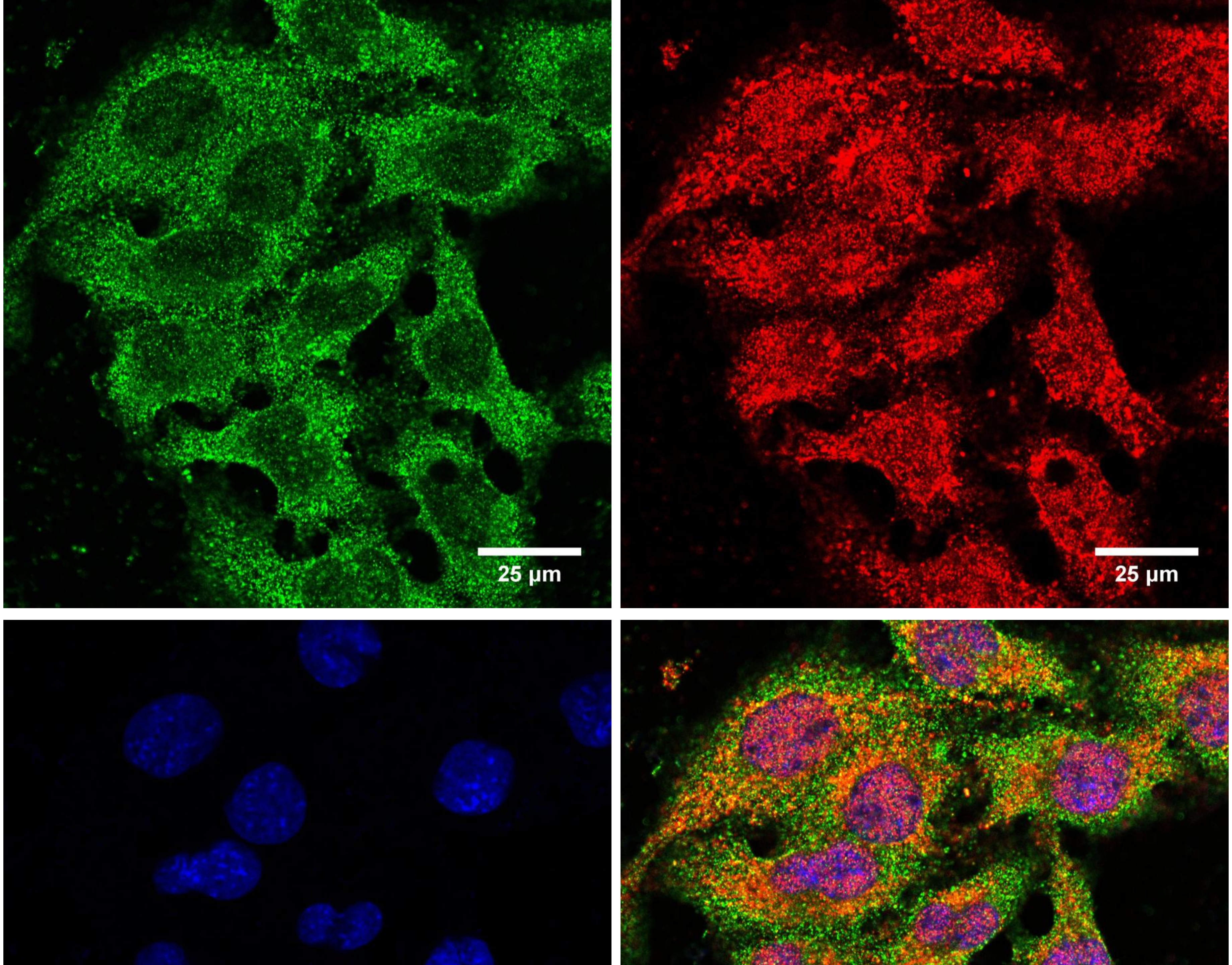

shMETTL3-1

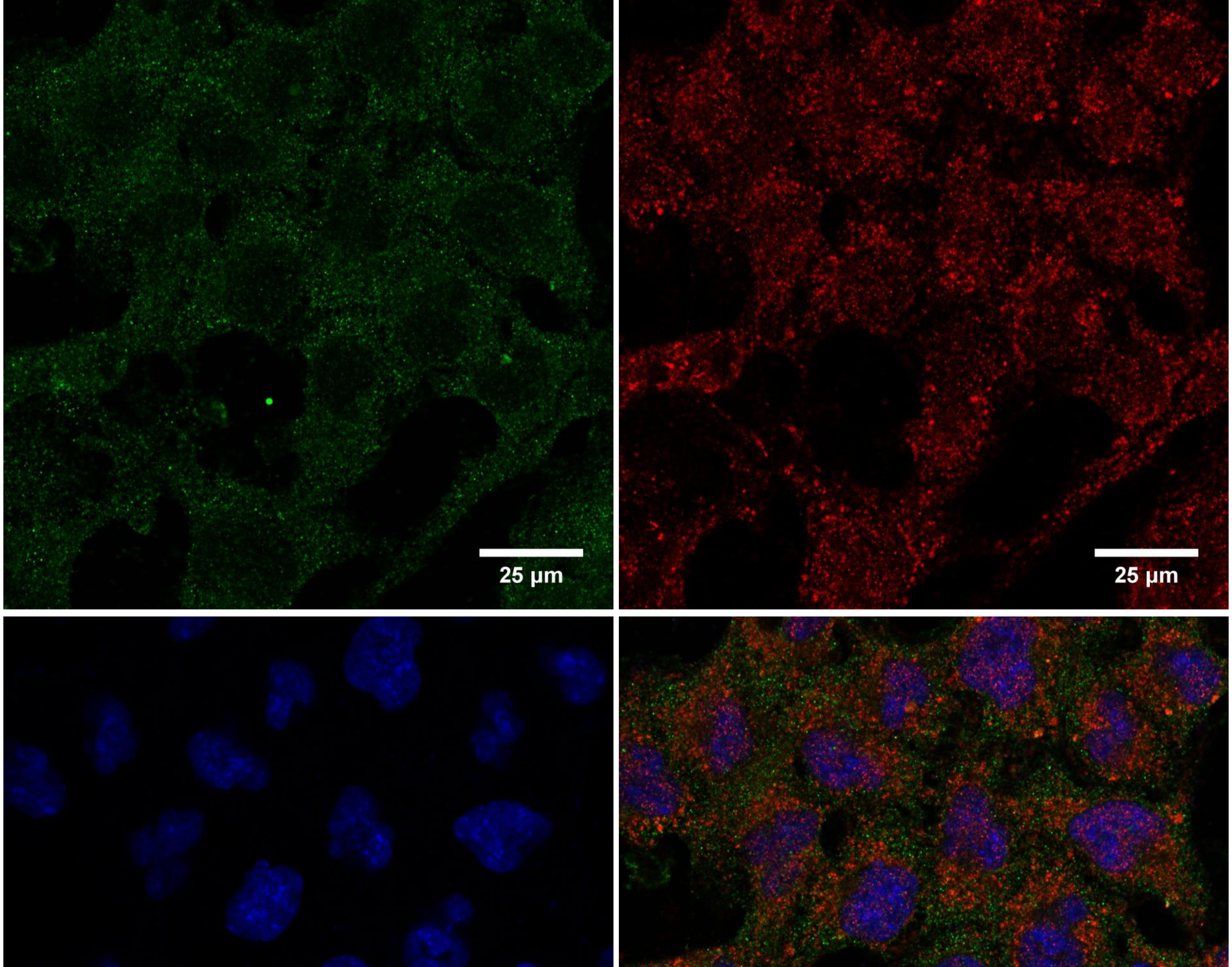

shMETTL3-2

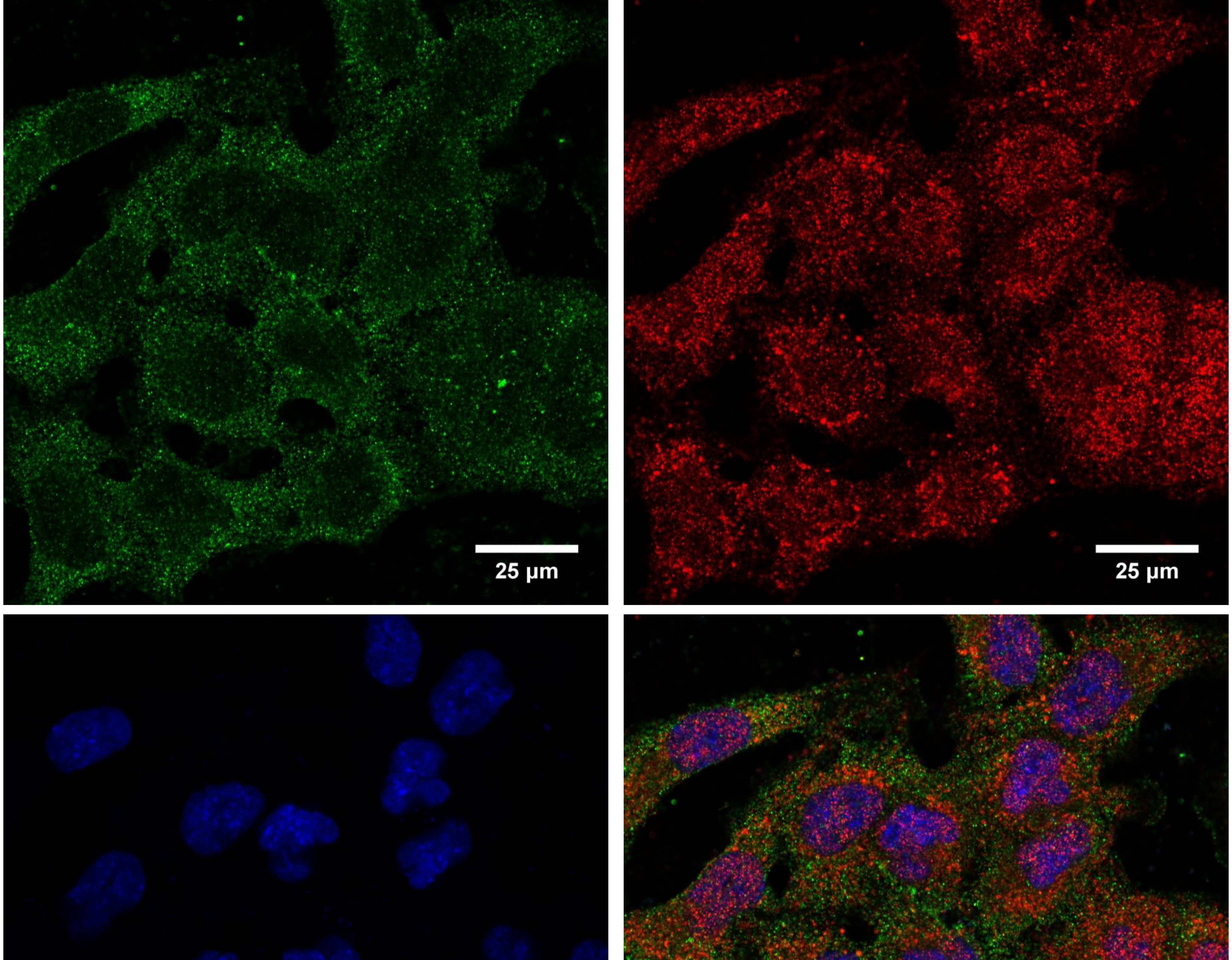

Figure S6

231-shNC

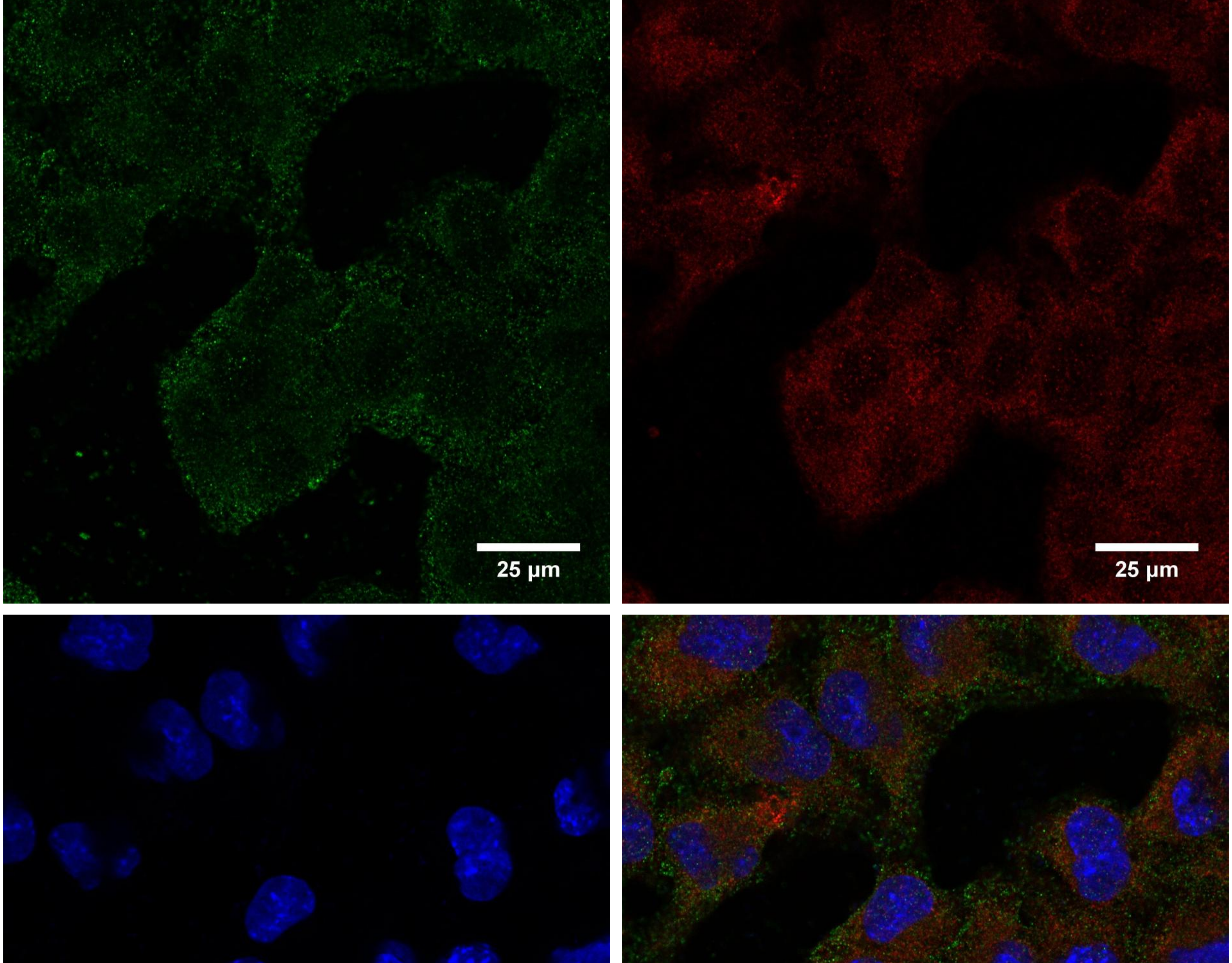

231-shIGF2-1

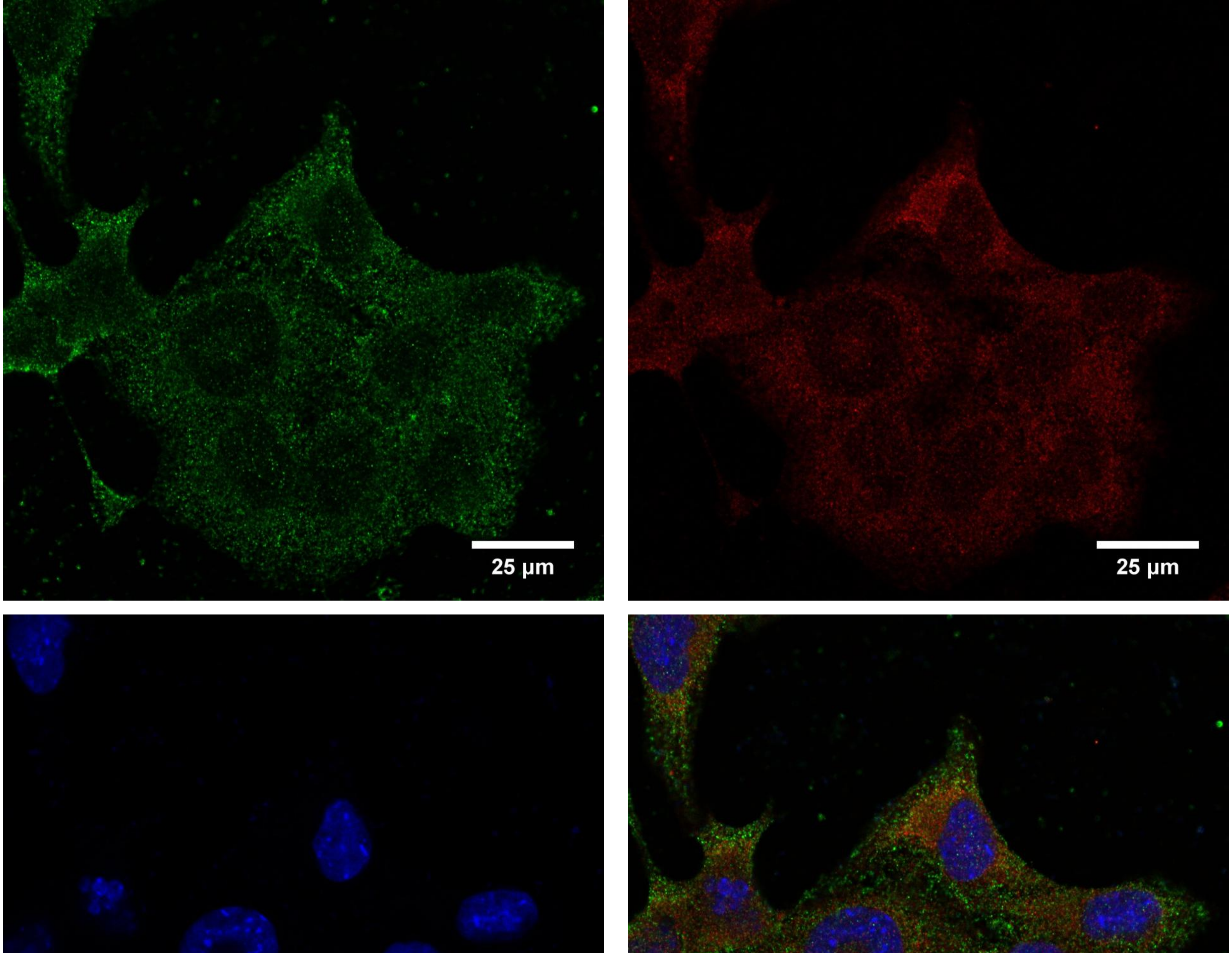

231-shIGF2-2

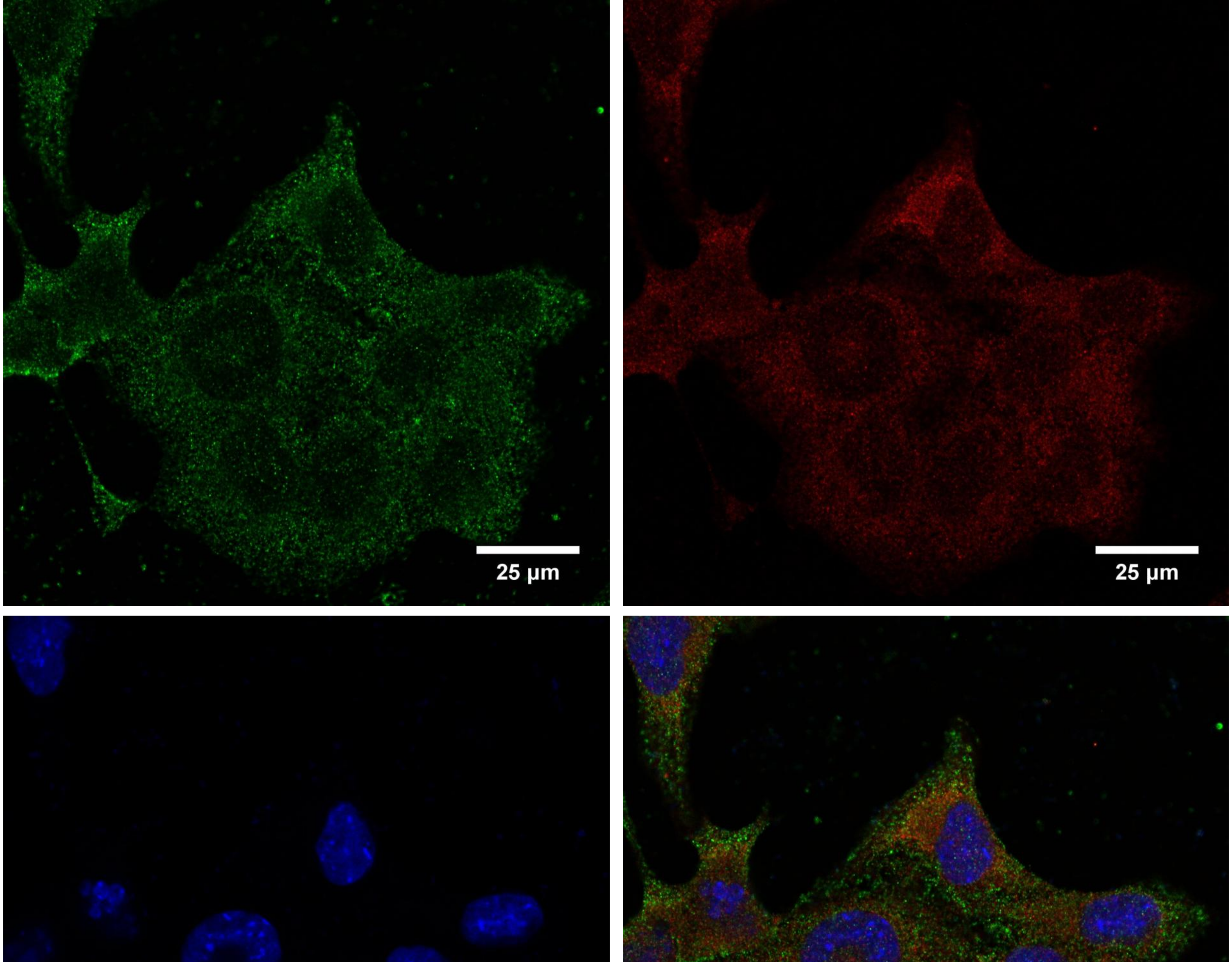

Figure S6

436-shNC

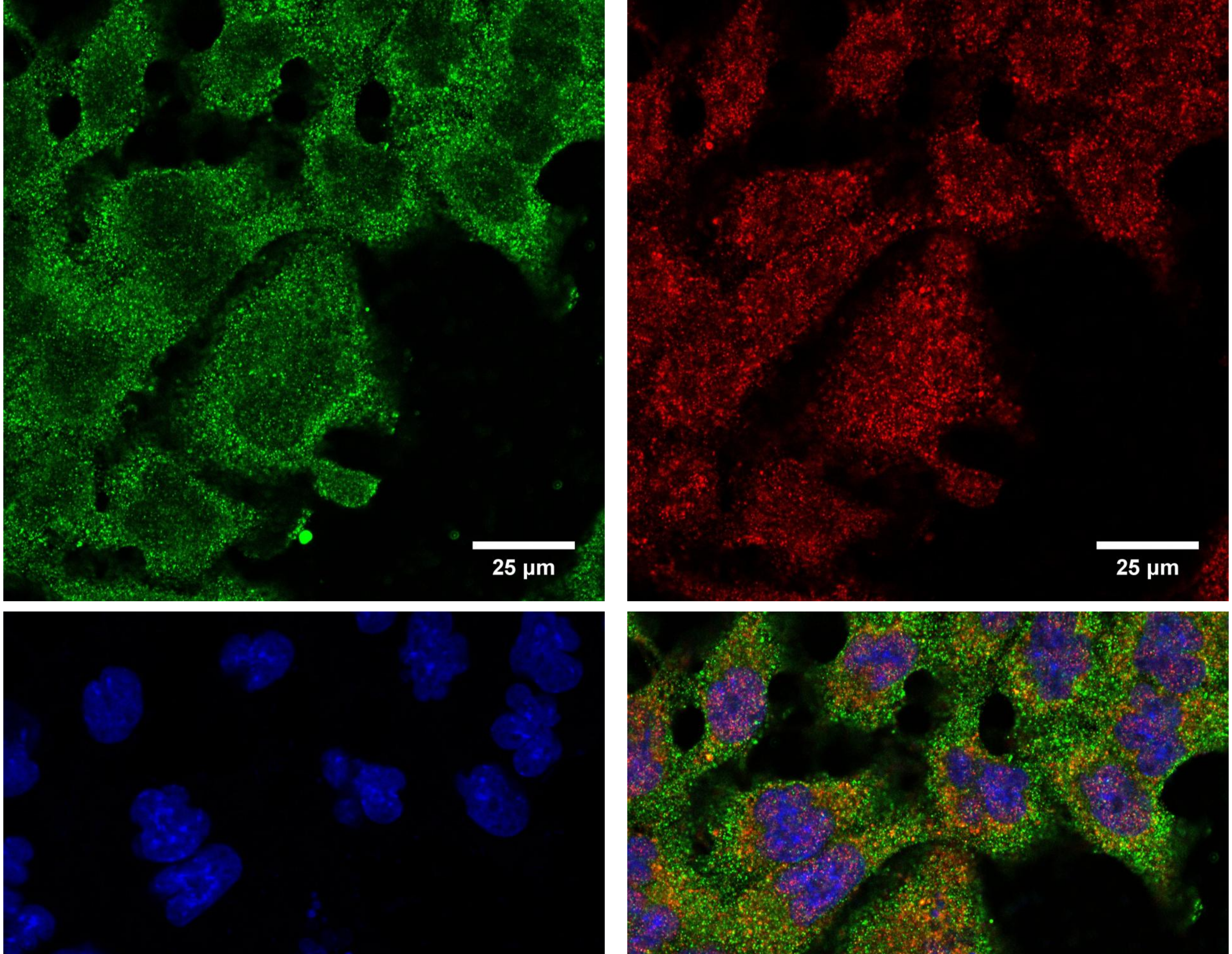

436-shIGF2-1

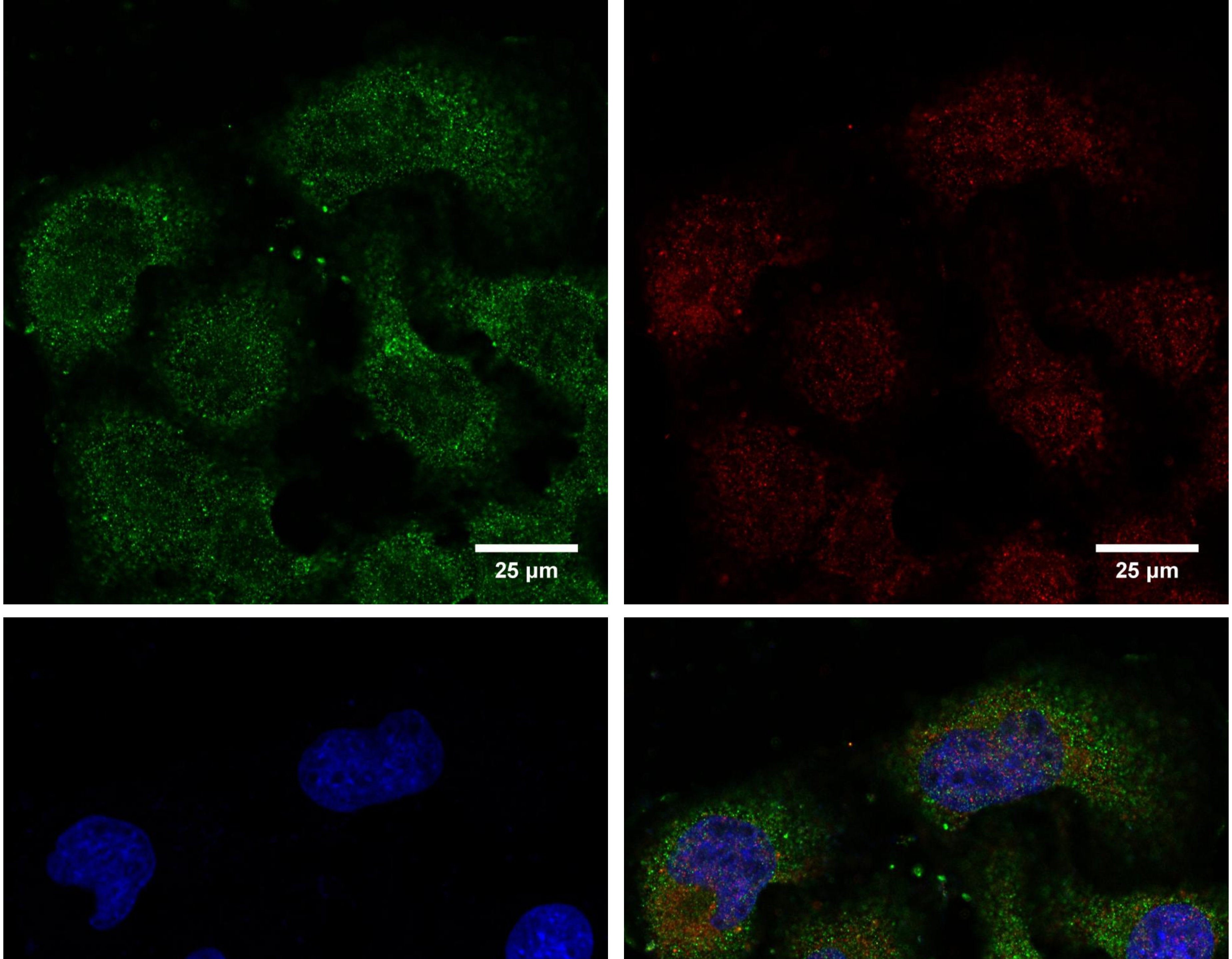

436-shIGF2-2

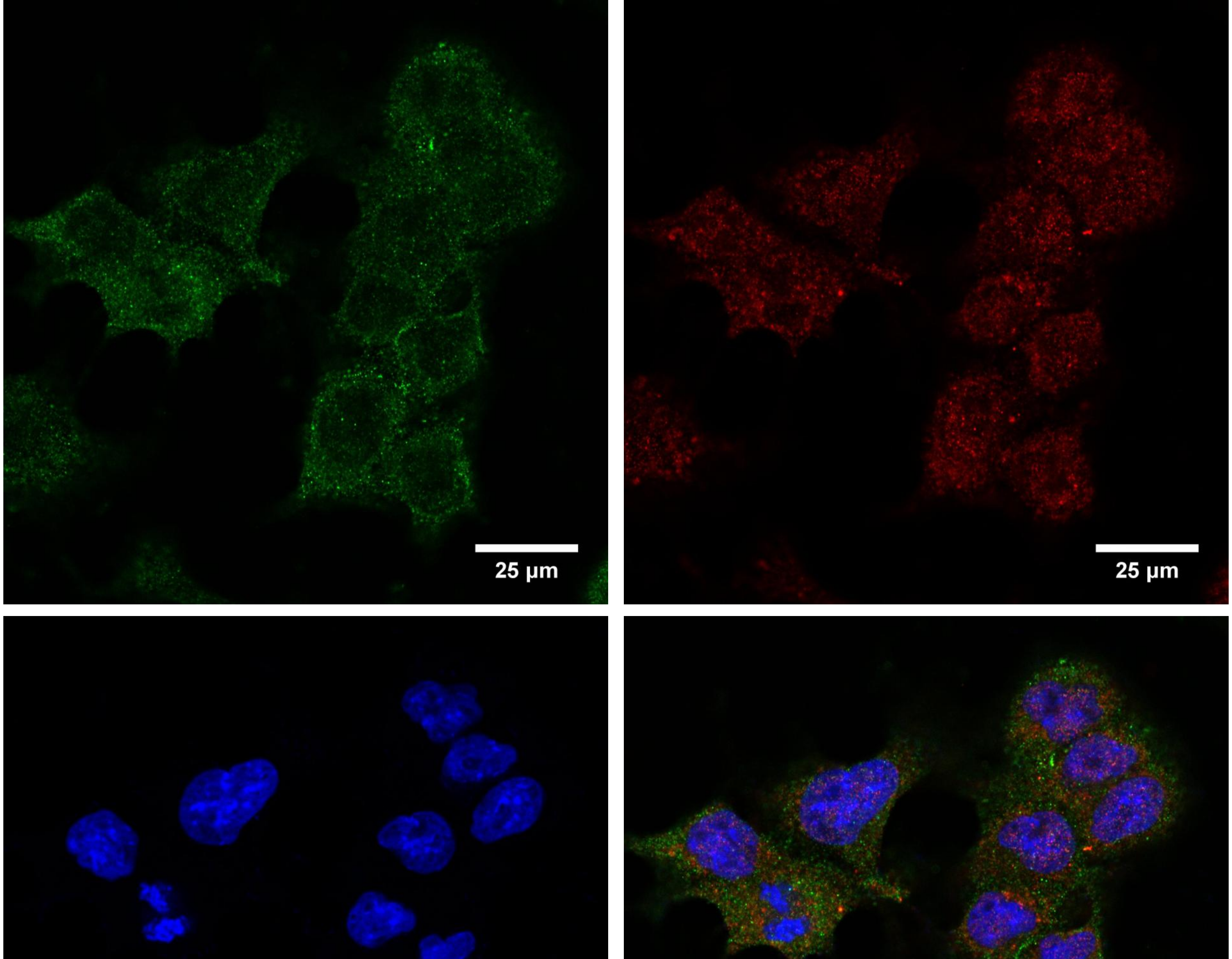

Supplement: Supplementary file 4 — IF figures [file 41419_2026_8845_MOESM4_ESM.pdf]
