# Supplementary material for: PLSCR1 drives chemoresistance in TNBC via METTL3/IGF2BP3-mediated mRNA stabilization and EGFR-MAPK pathway activation
Source: Cell Death Dis. 2026 May 15;17(1):624. doi: 10.1038/s41419-026-08845-4 (PMC13347015; doi:10.1038/s41419-026-08845-4)

Figure 1d

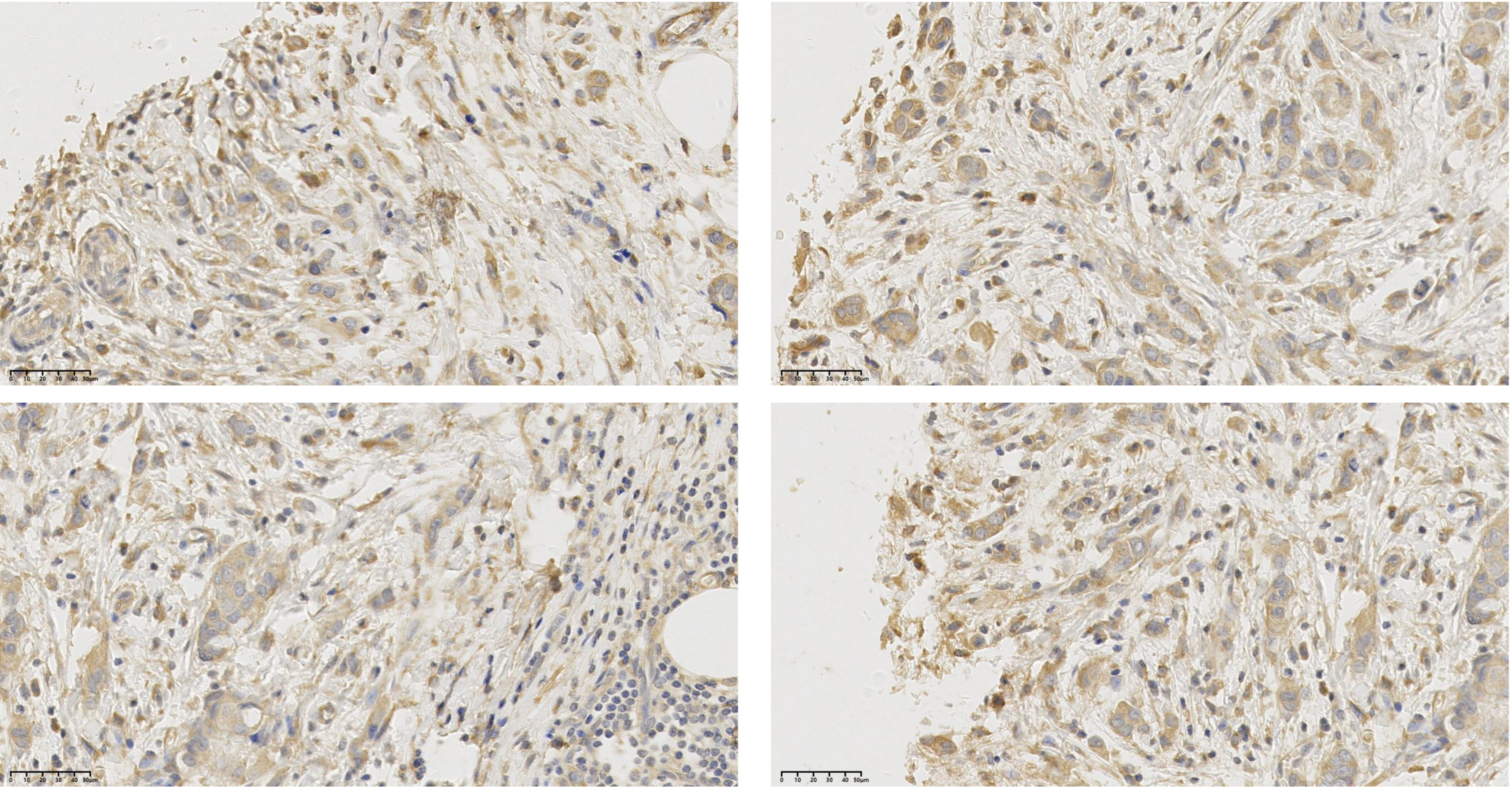
0 10 20 30 40 50μm
0 10 20 30 40 50μm
0 10 20 30 40 50μm
0 10 20 30 40 50μm

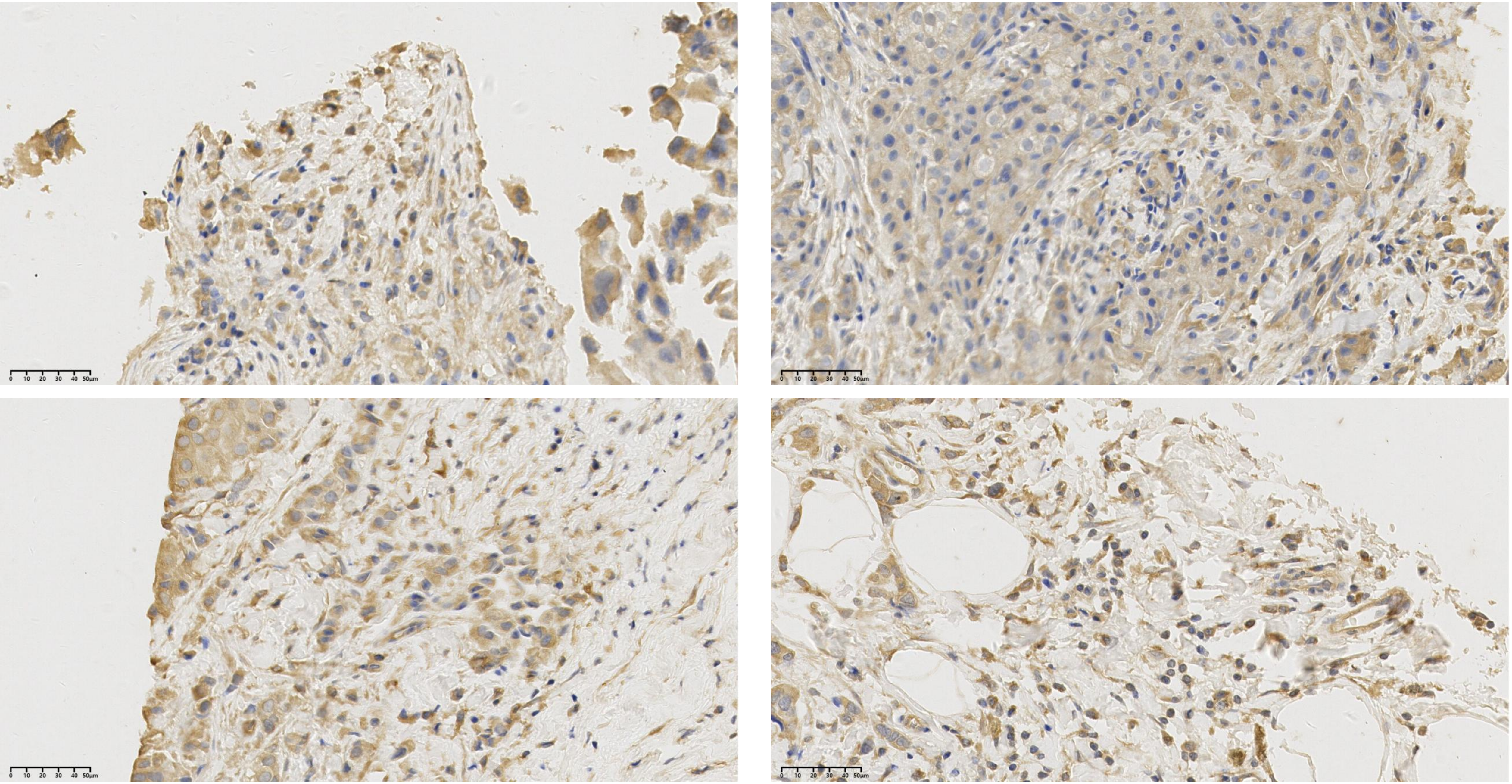
0 10 20 30 40 50μm
0 10 20 30 40 50μm
0 10 20 30 40 50μm
0 10 20 30 40 50μm

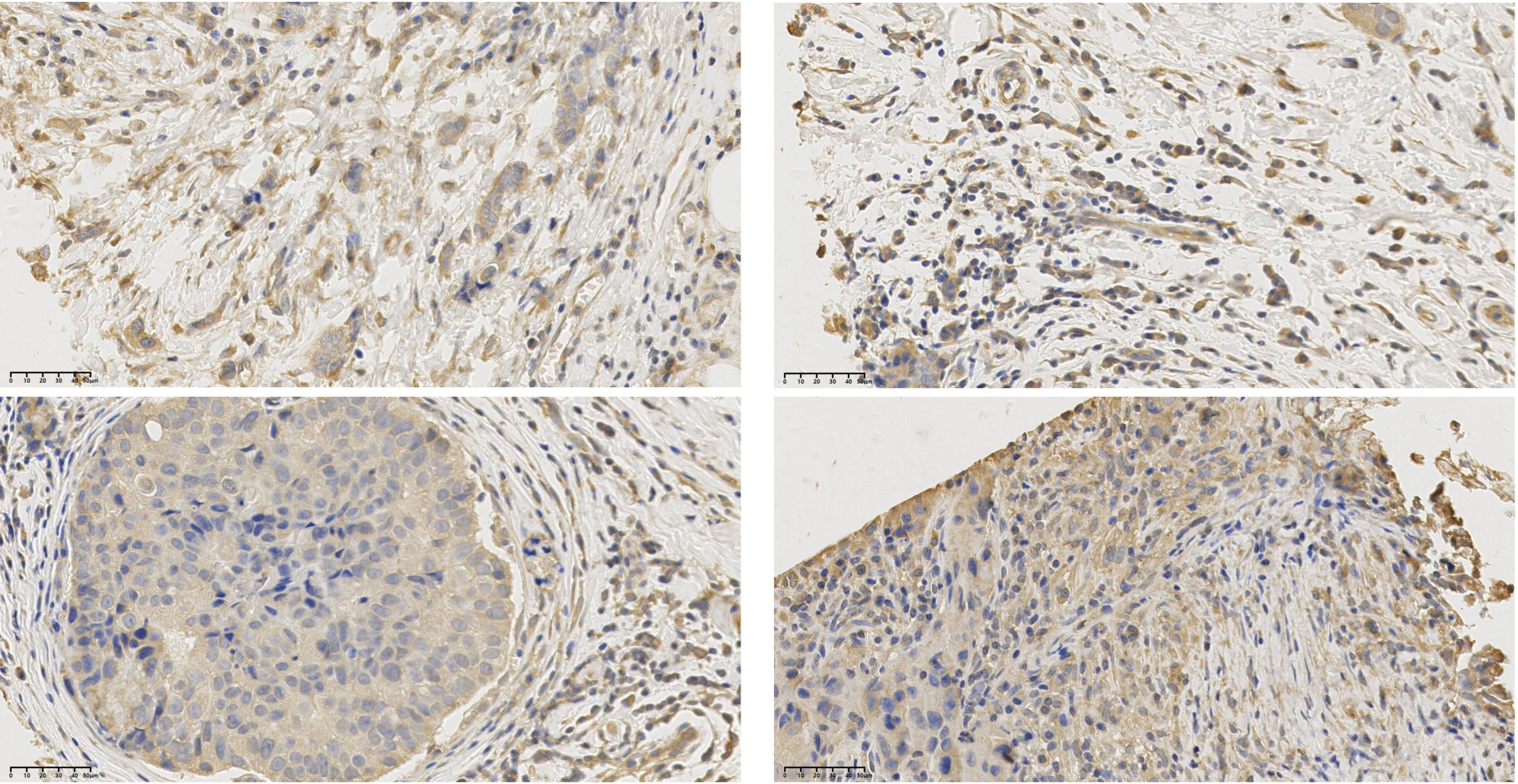
0 10 20 30 40 50µm

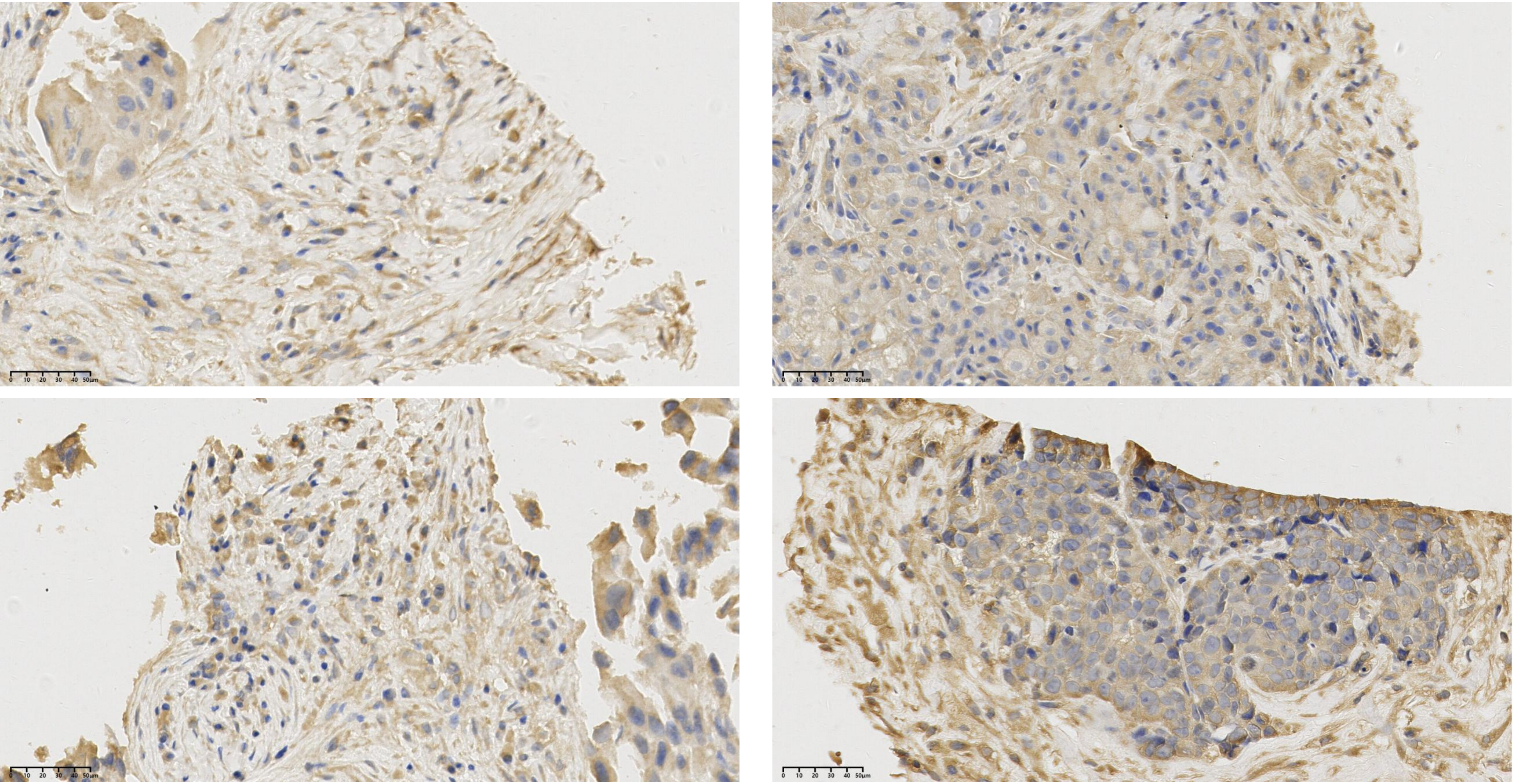
0 10 20 30 40 50µm
0 10 20 30 40 50µm
0 10 20 30 40 50µm
0 10 20 30 40 50µm

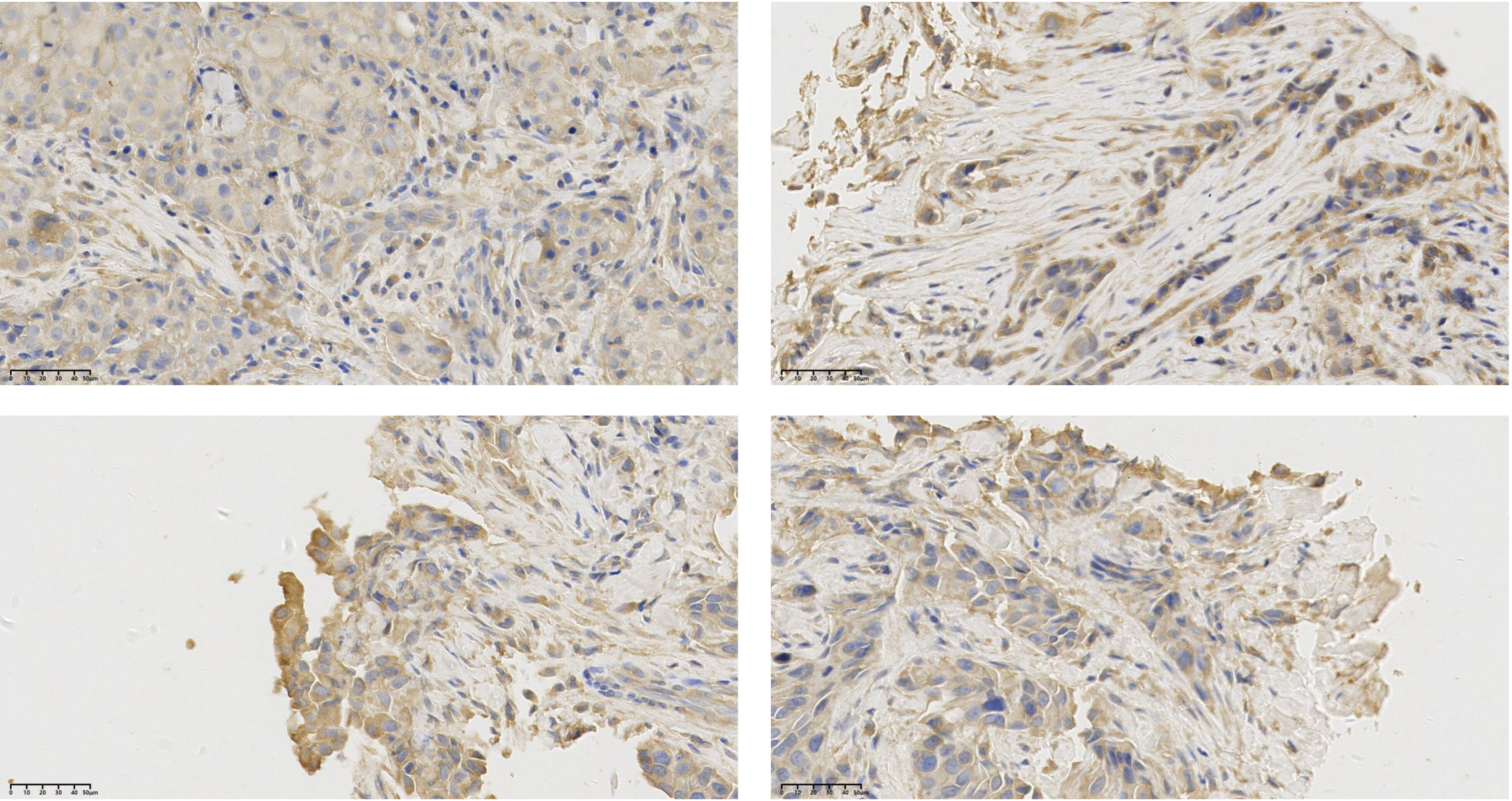
0 10 20 30 40 50μm
0 10 20 30 40 50μm
0 10 20 30 40 50μm
0 10 20 30 40 50μm

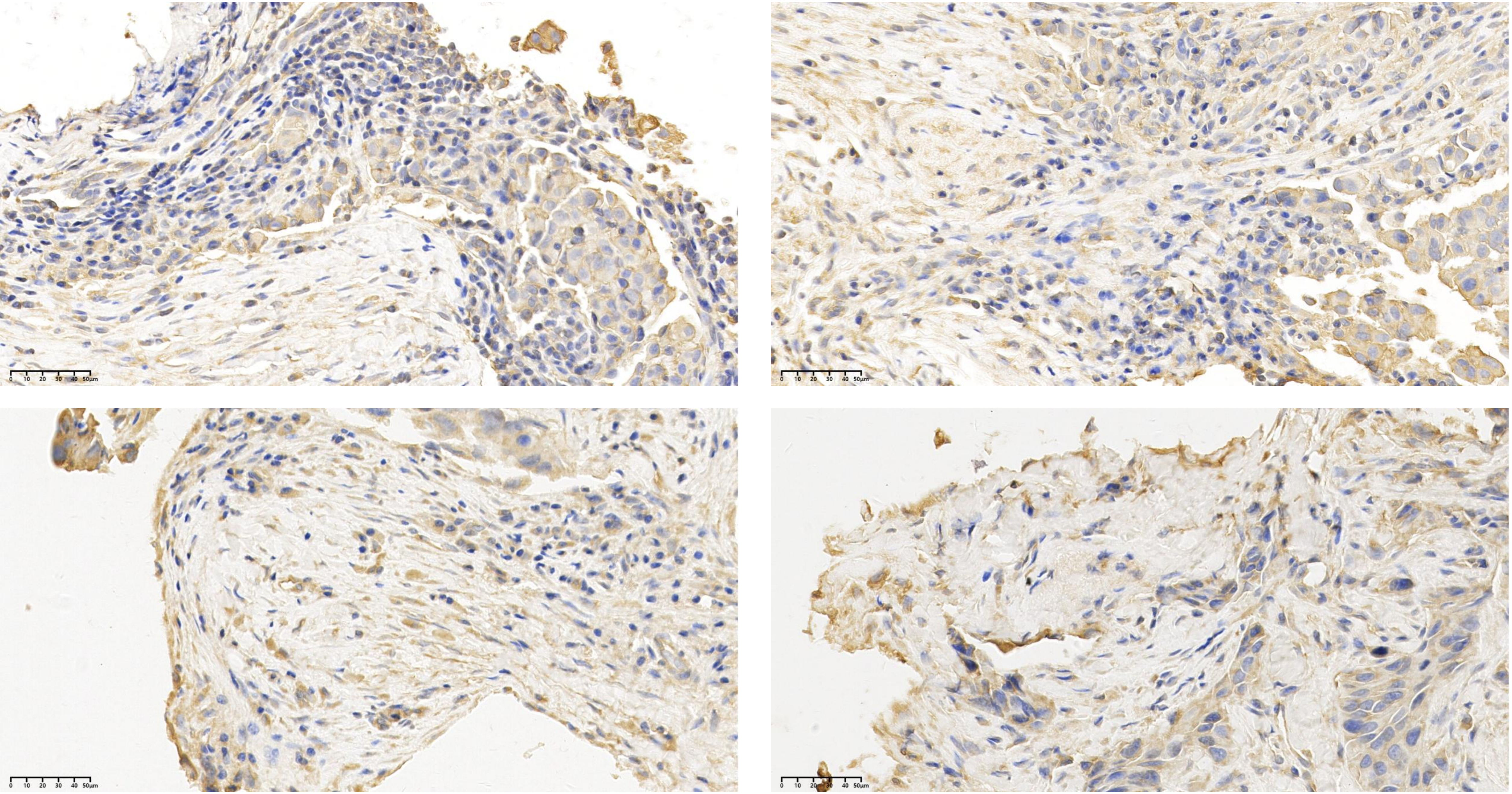
0 10 20 30 40 50μm
0 10 20 30 40 50μm
0 10 20 30 40 50μm
0 10 20 30 40 50μm

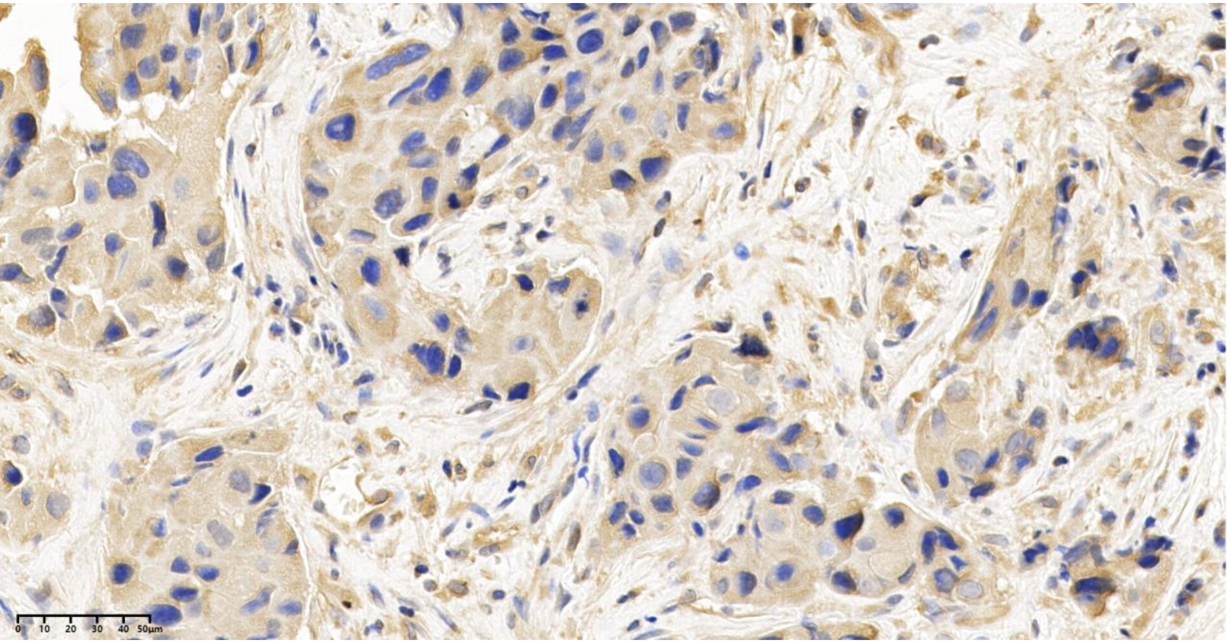
0 10 20 30 40 50μm
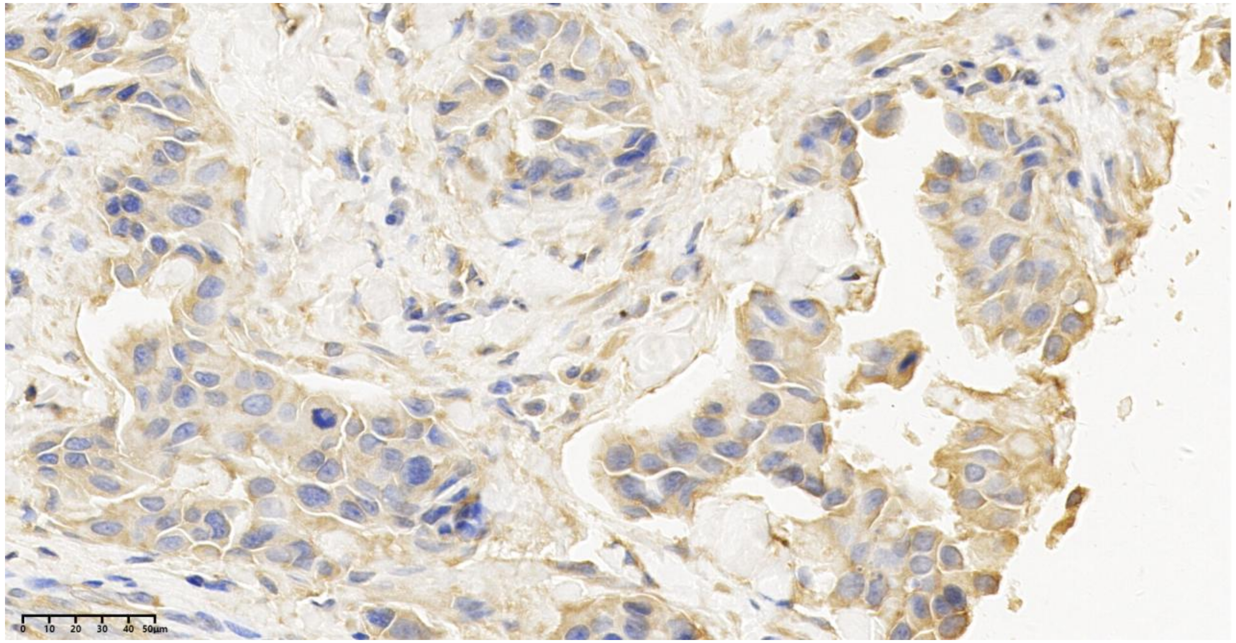
0 10 20 30 40 50μm
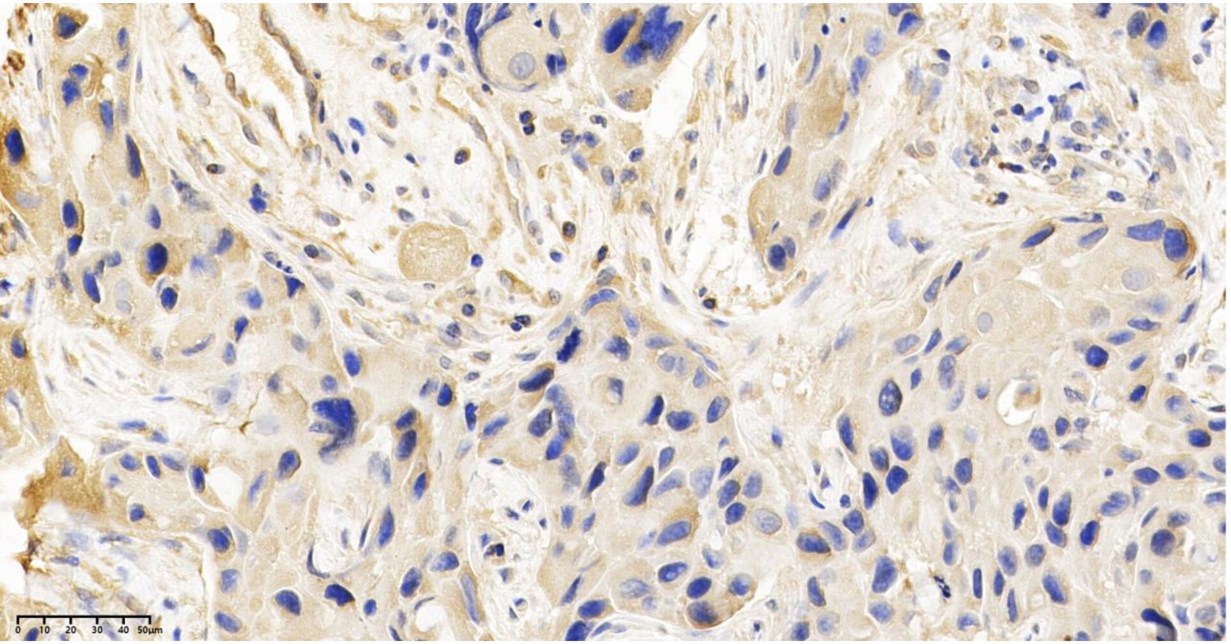
0 10 20 30 40 50μm
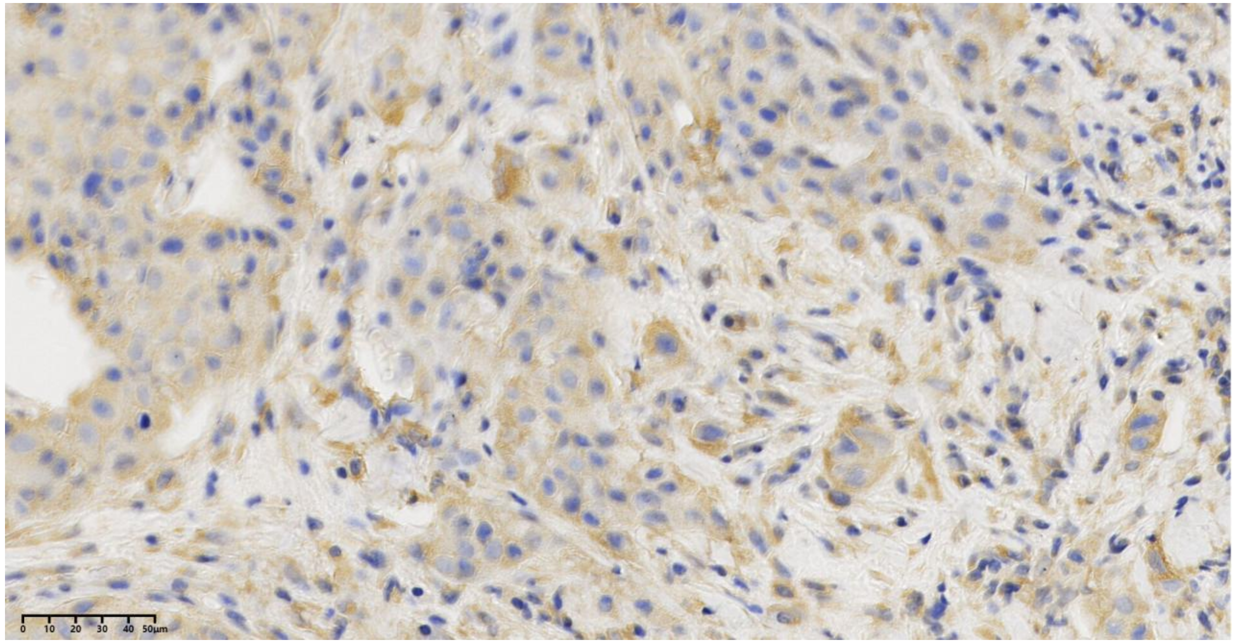
0 10 20 30 40 50μm

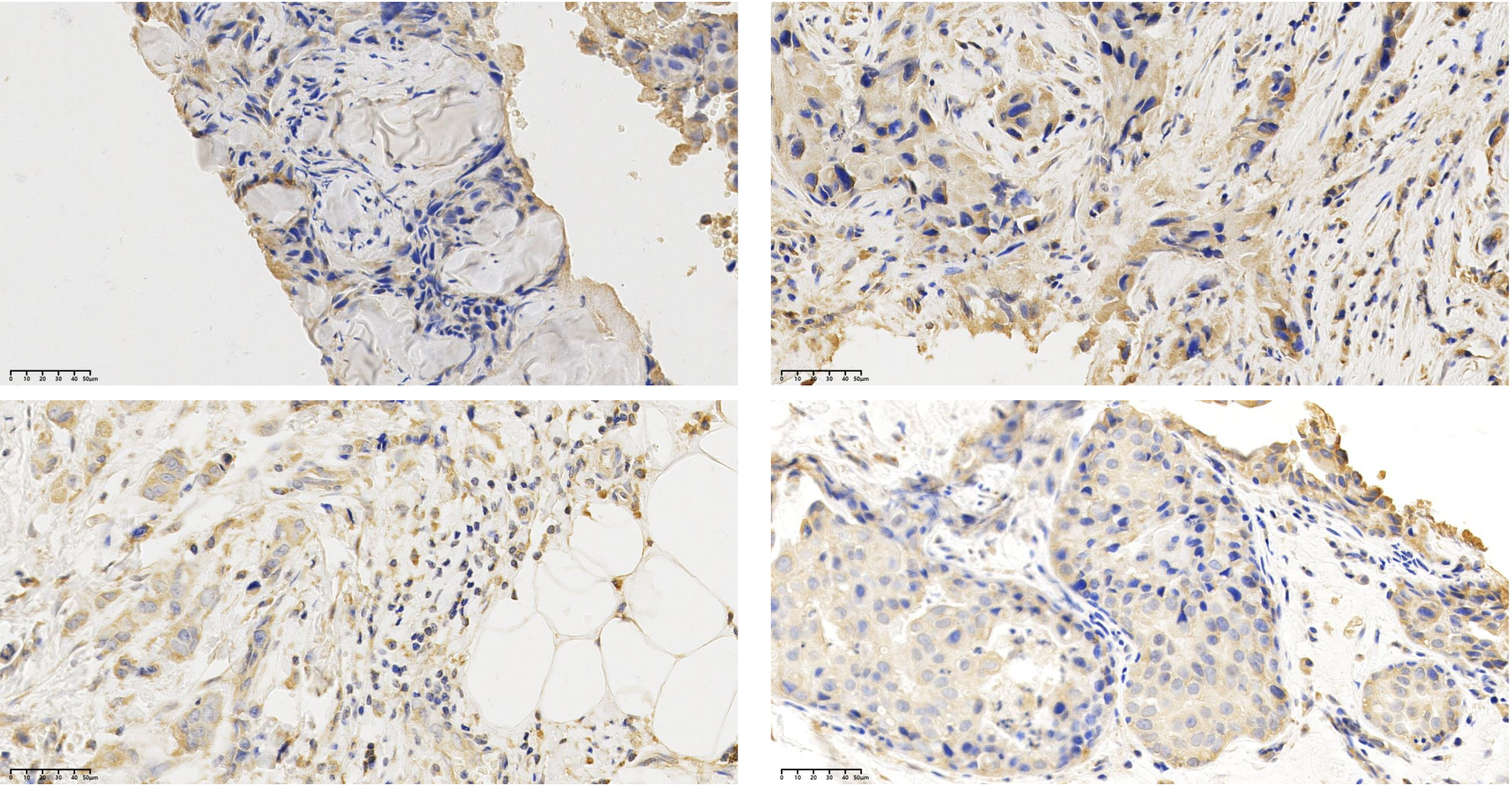
0 10 20 30 40 50μm
0 10 20 30 40 50μm
0 10 20 30 40 50μm
0 10 20 30 40 50μm

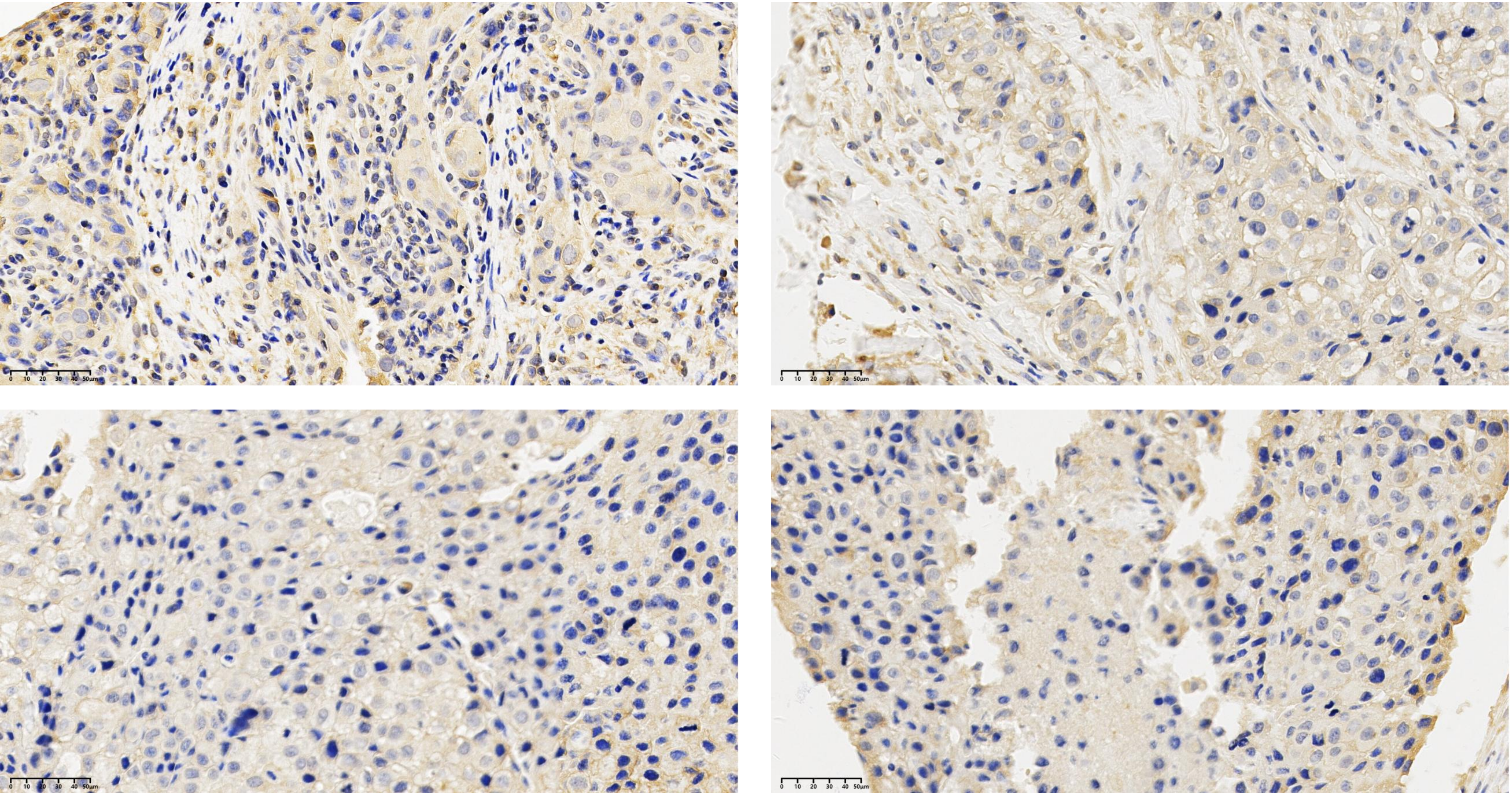
0 10 20 30 40 50μm
0 10 20 30 40 50μm
0 10 20 30 40 50μm
0 10 20 30 40 50μm

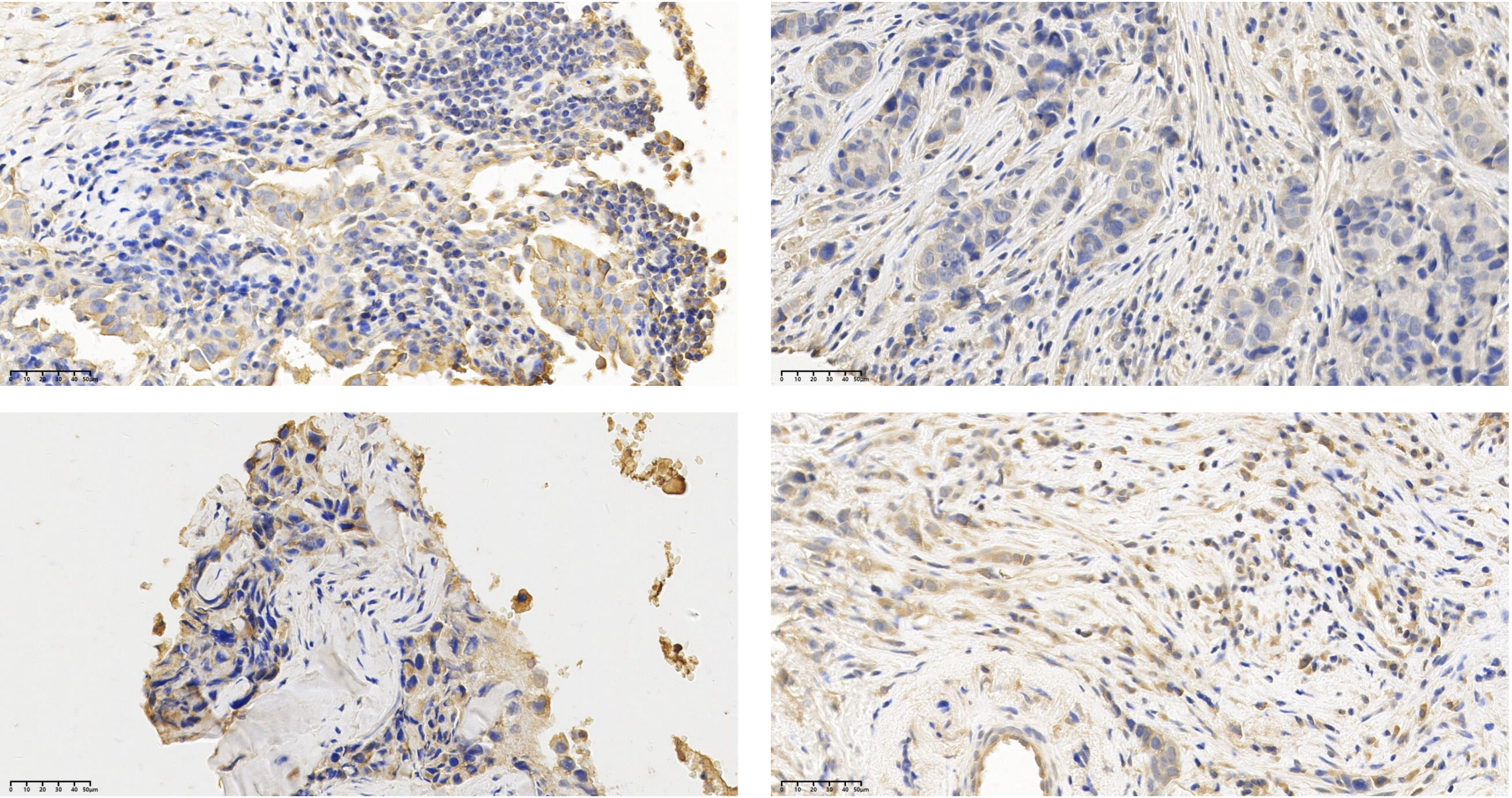
0 10 20 30 40 50μm

Figure 2h

Anti-PLSCR1

shNC

shPLSCR1

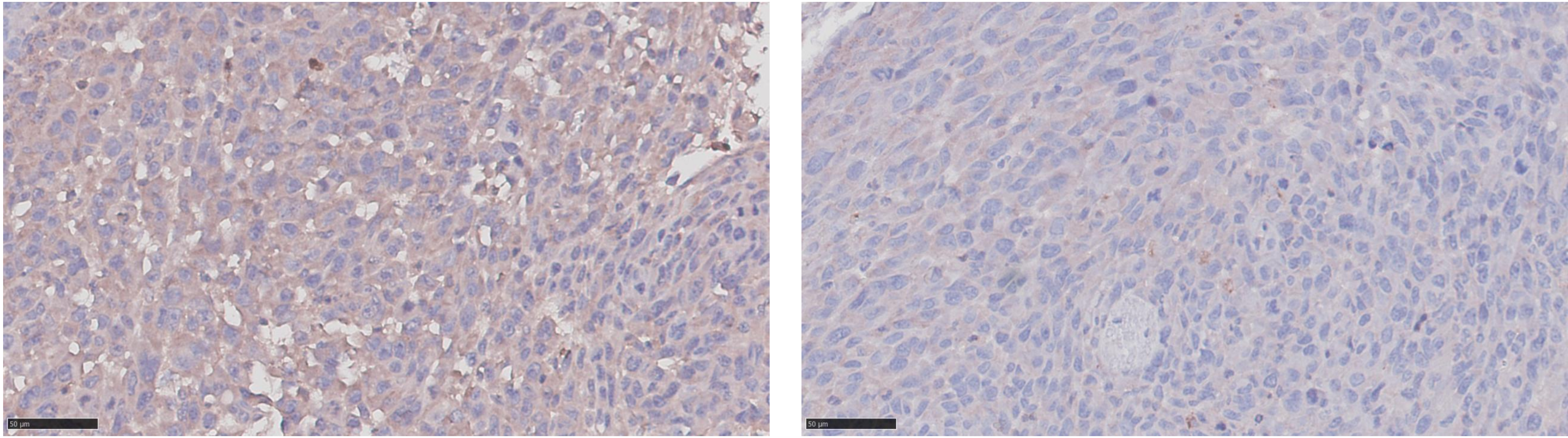

Anti-Ki67

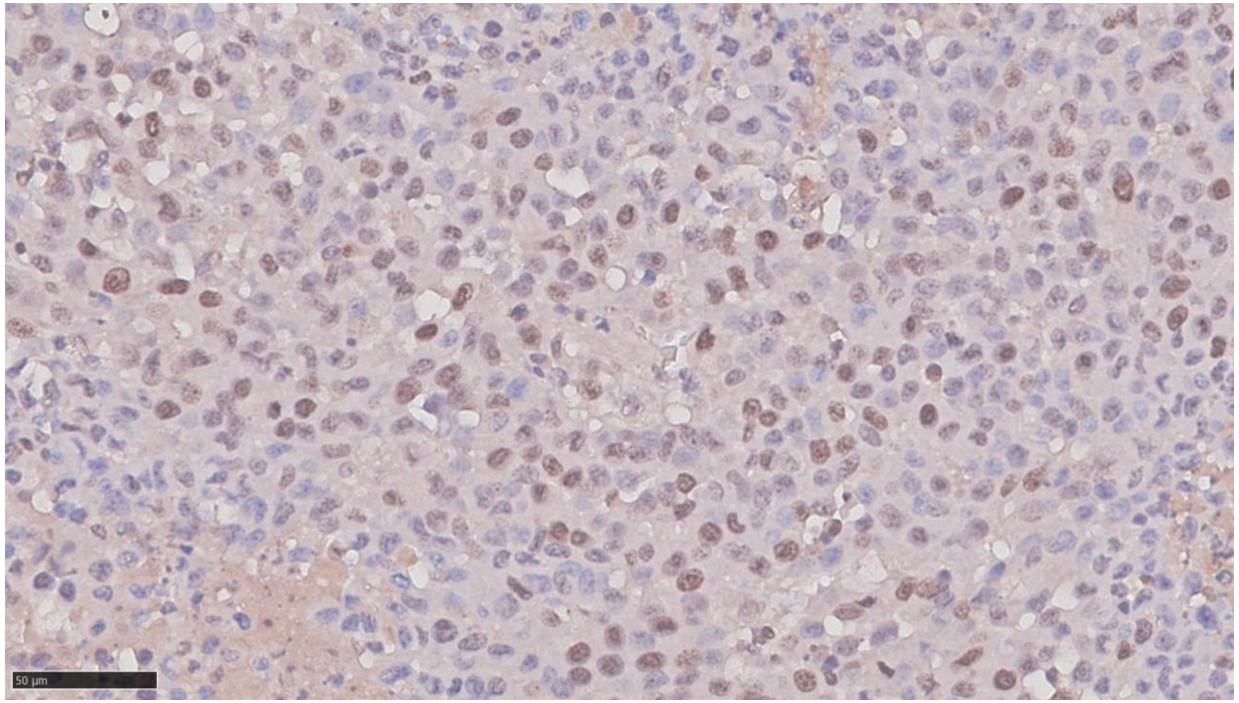

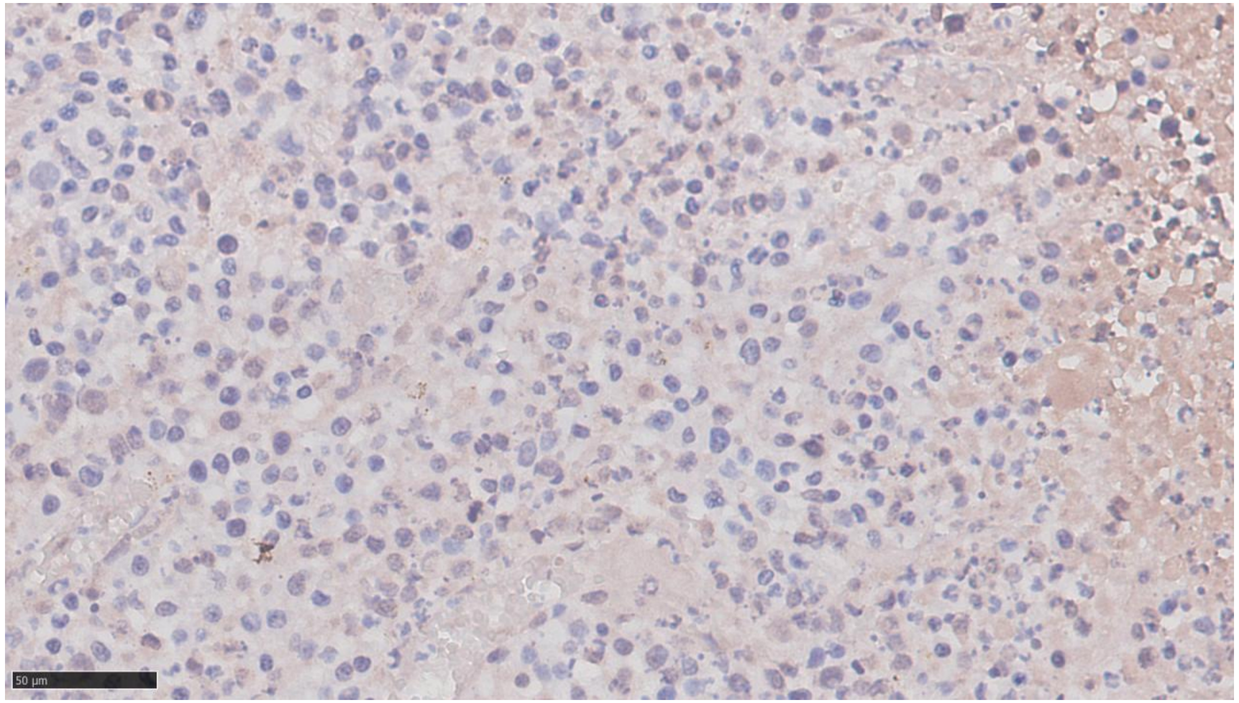

Figure S3

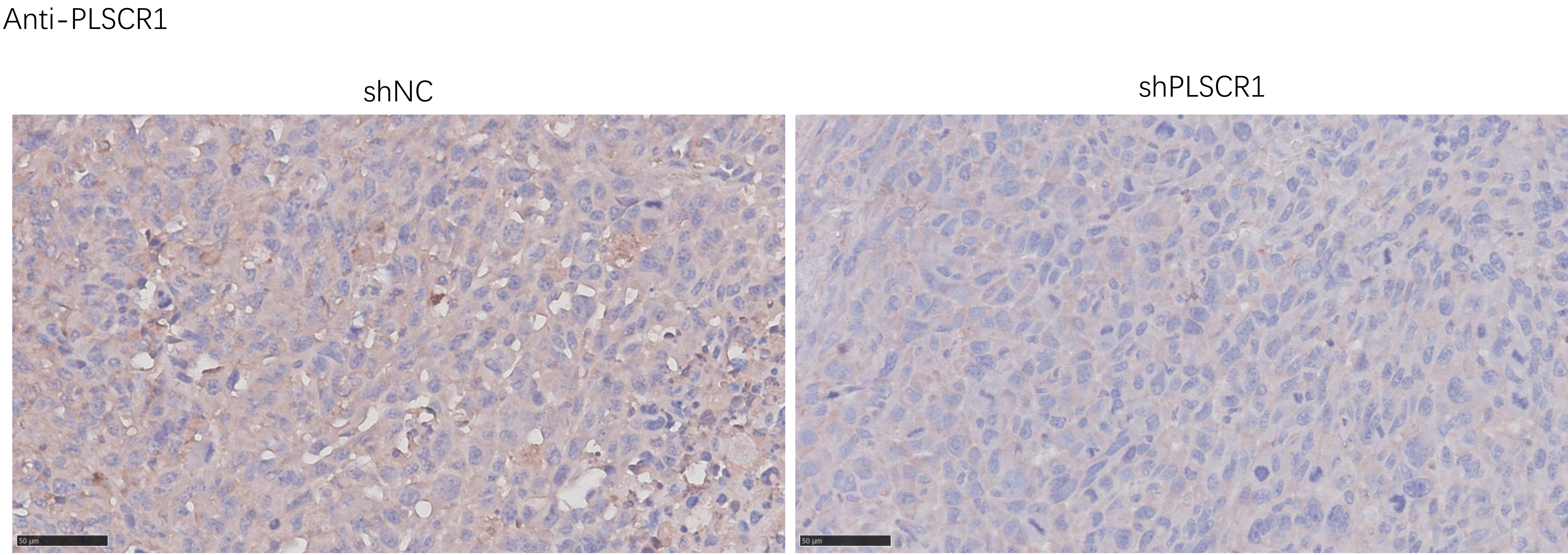

Anti-Ki67

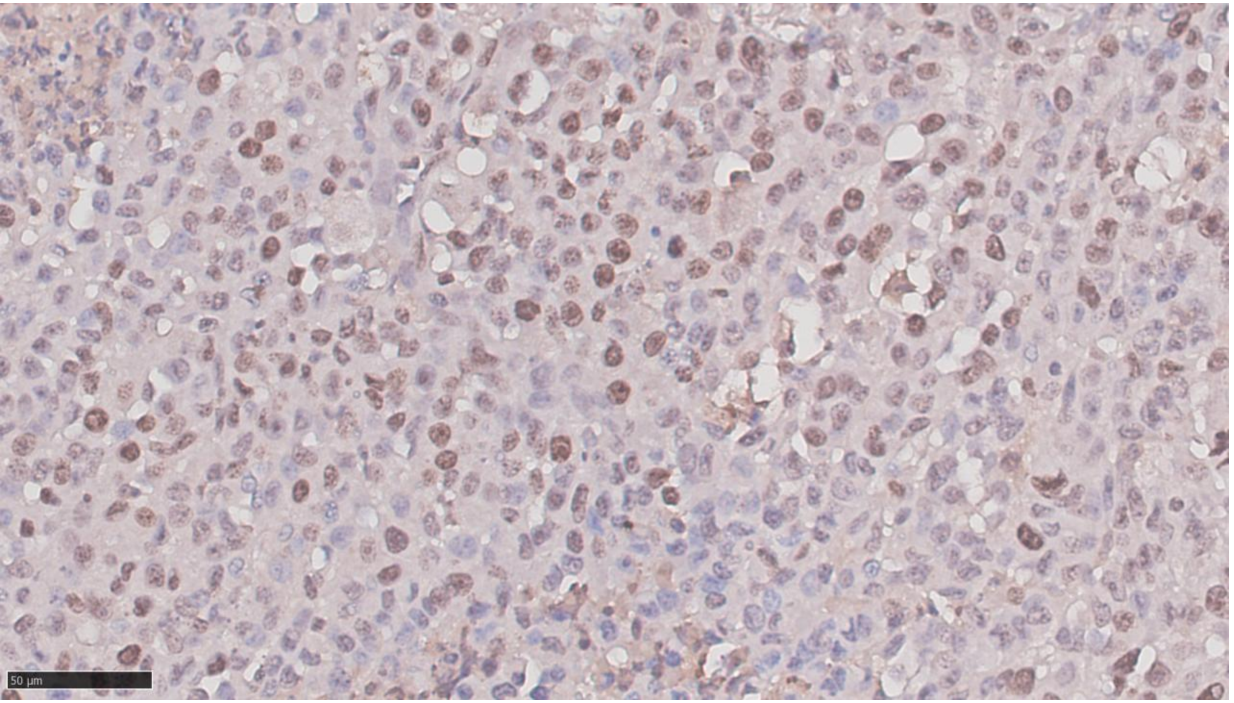

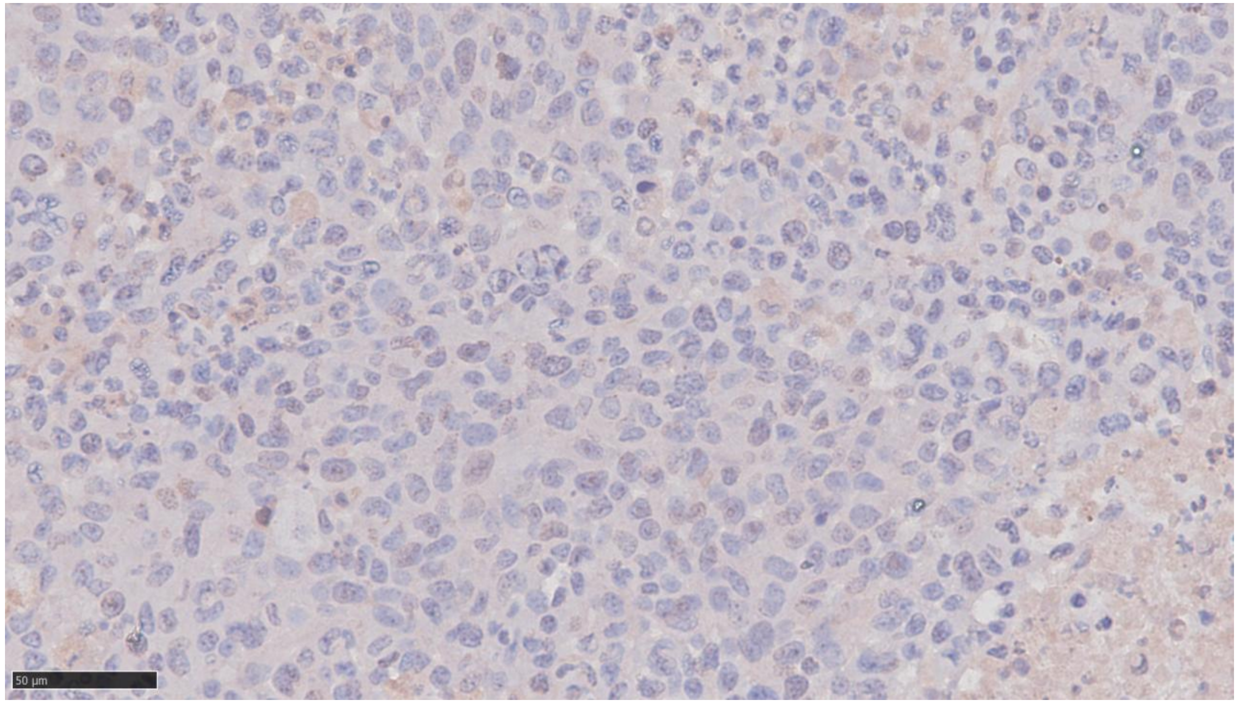

Figure 7

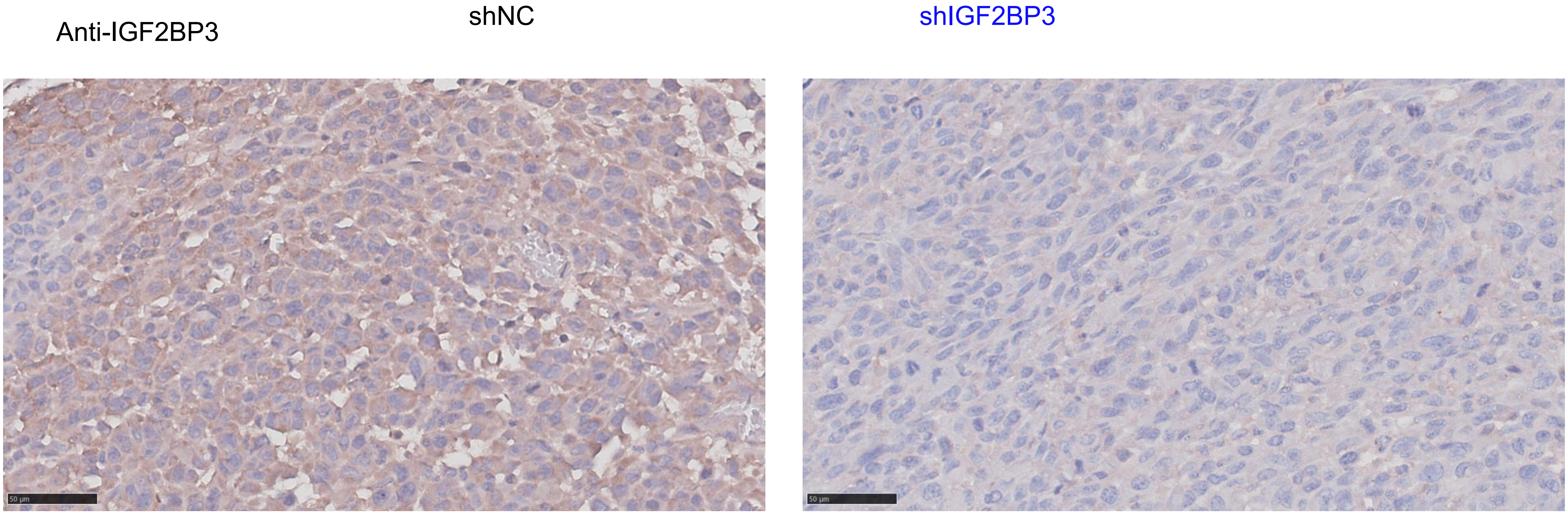

shIGF2BP3 +OE-Vec

shIGF2BP3 +OE-PLSCR1

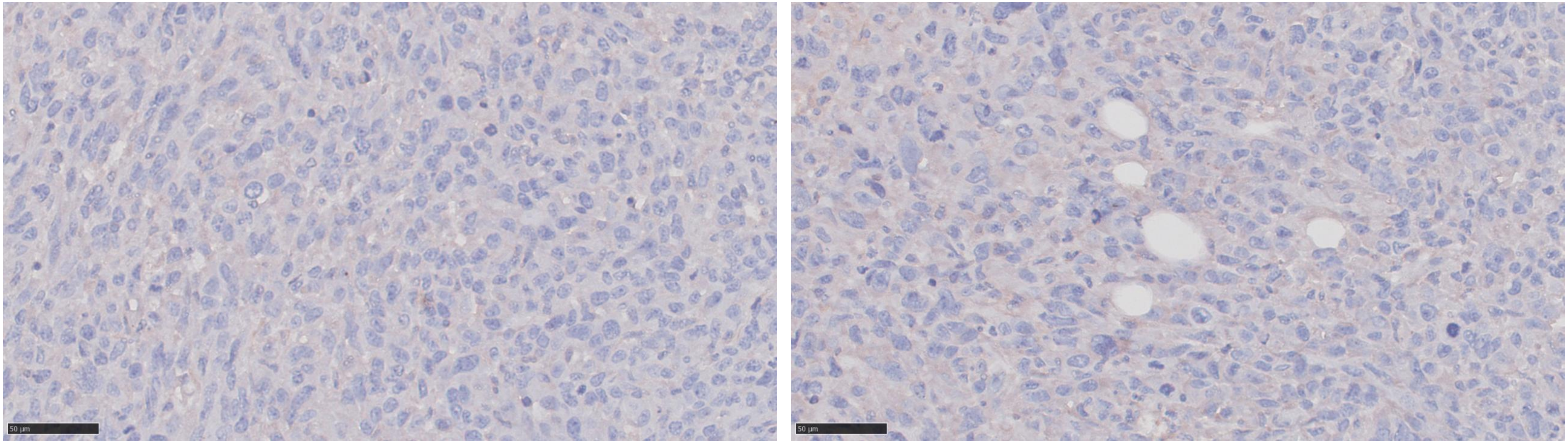

Anti-PLSCR1

shNC

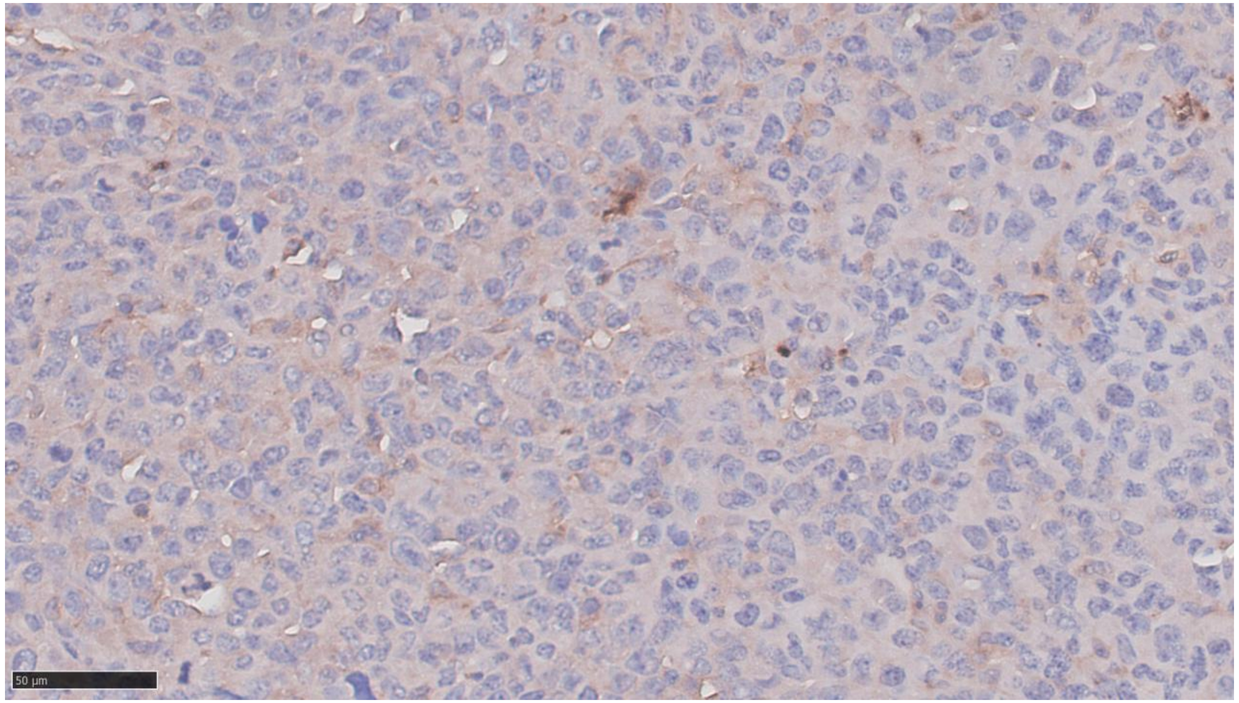

shIGF2BP3

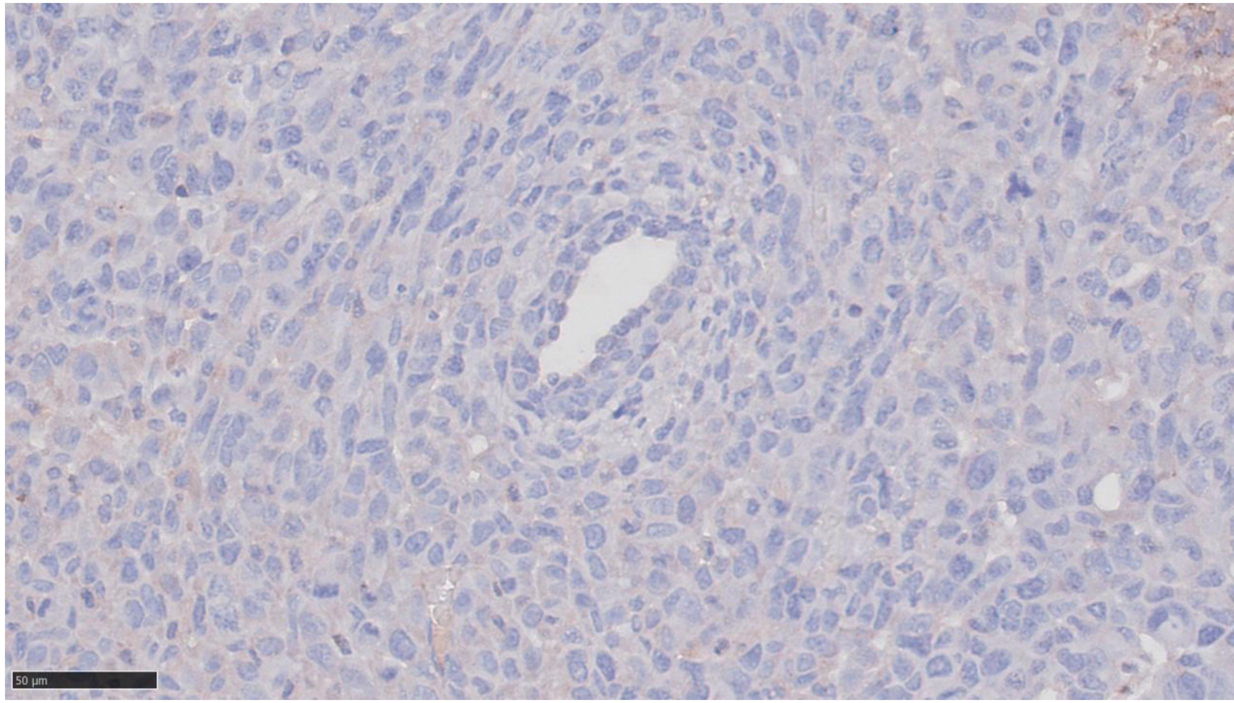

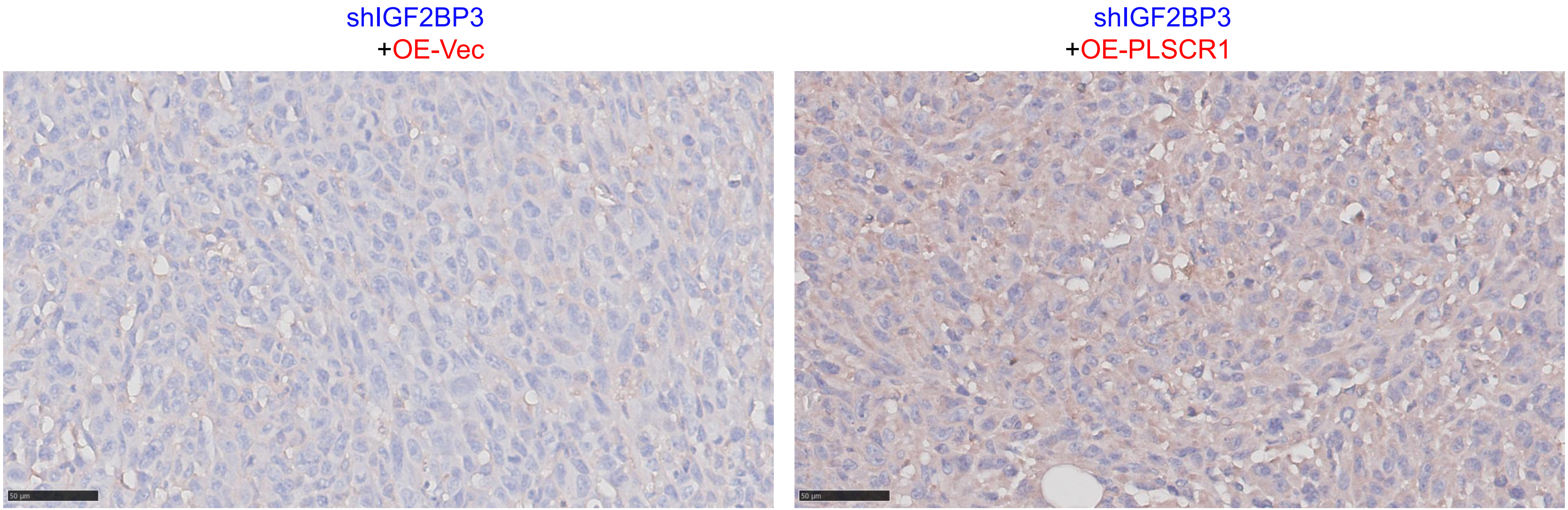
shIGF2BP3
+OE-Vec
50 µm
shIGF2BP3
+OE-PLSCR1
50 µm

Anti-Ki67

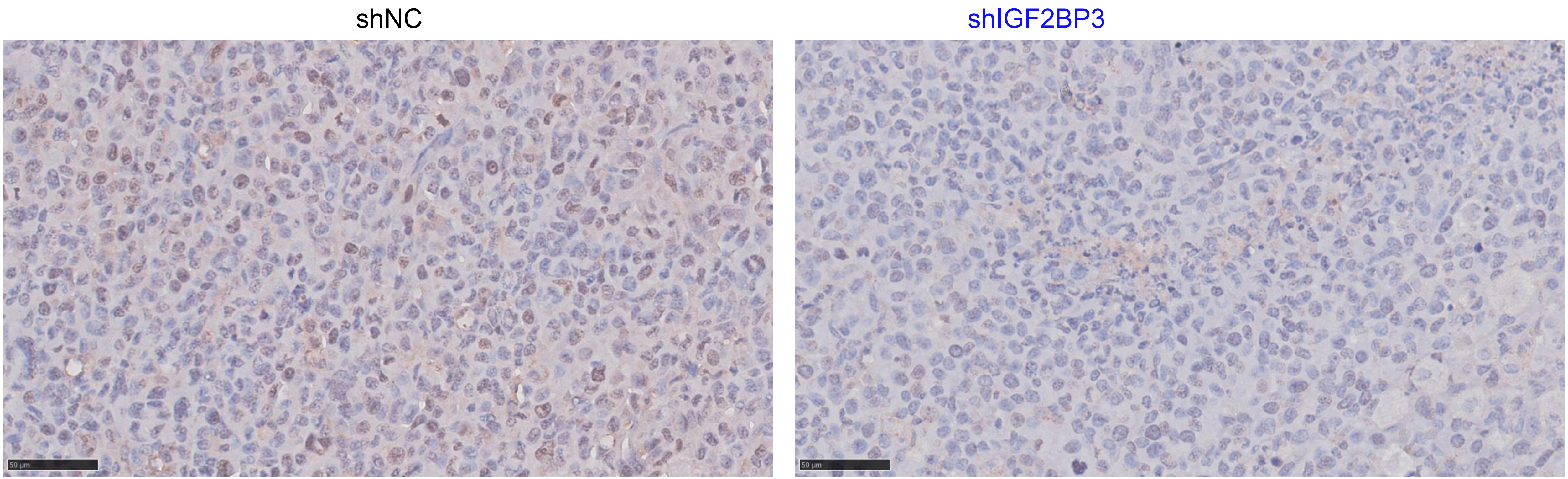

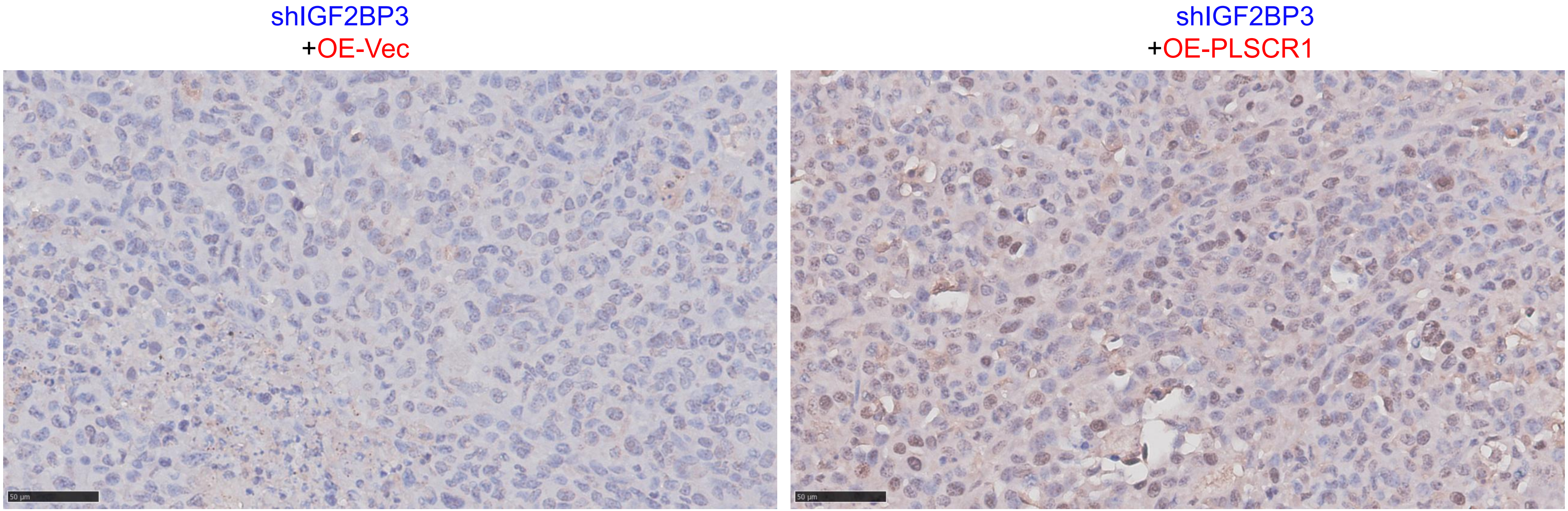
shIGF2BP3
+OE-Vec
shIGF2BP3
+OE-PLSCR1
50 µm
50 µm

Figure S7

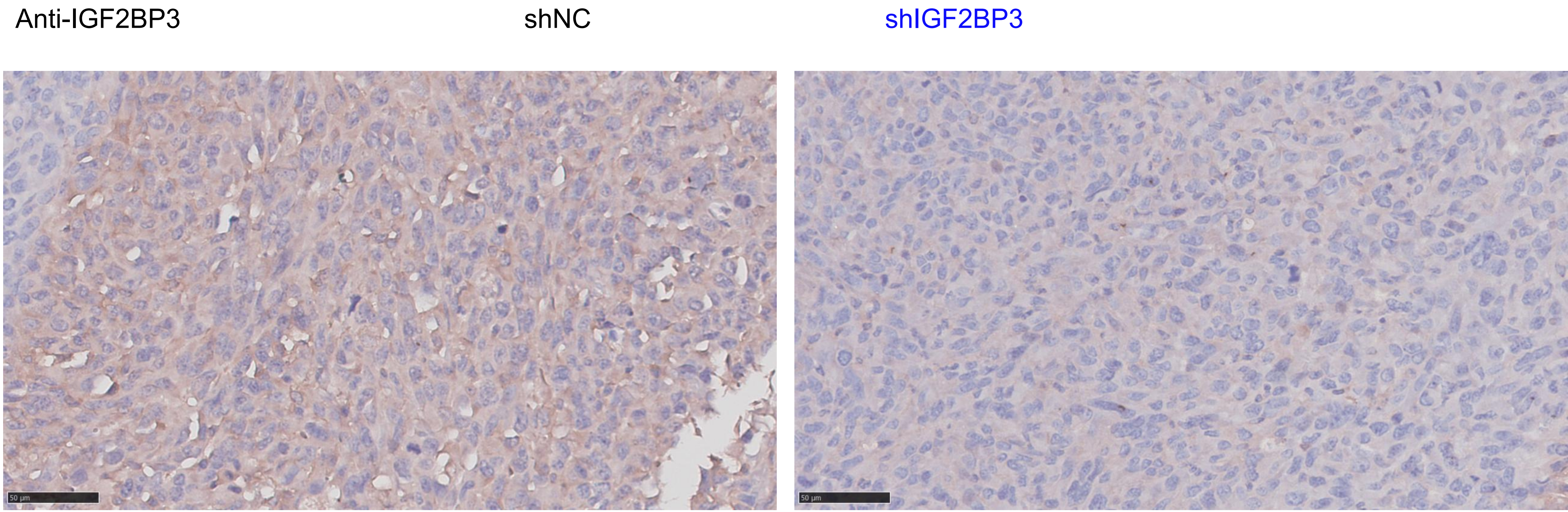

shIGF2BP3 +OE-Vec

shIGF2BP3 +OE-PLSCR1

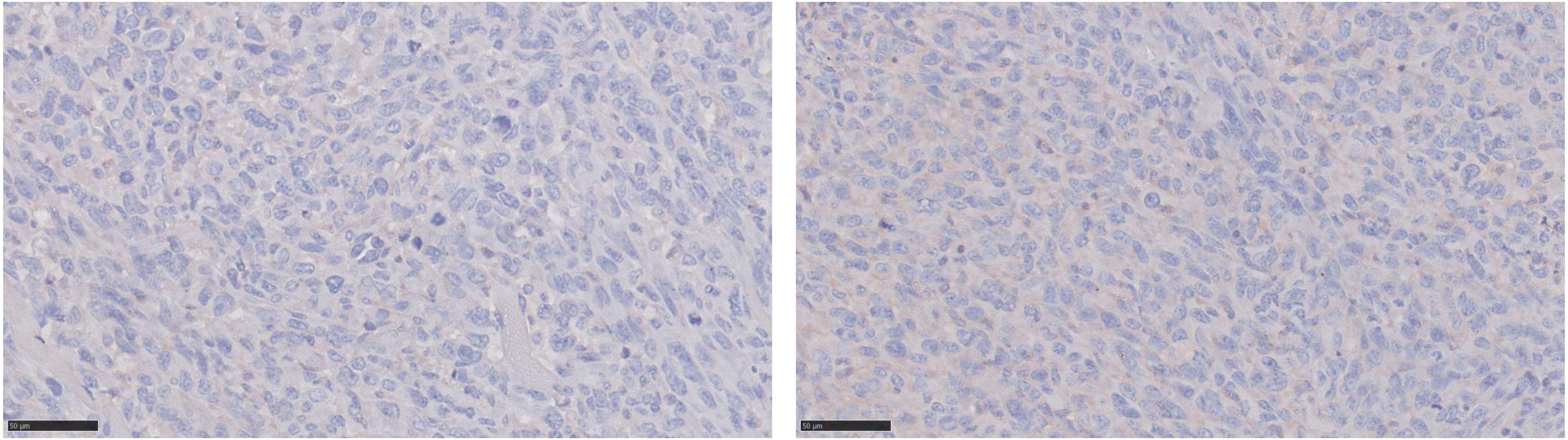

Anti-PLSCR1 shNC shIGF2BP3

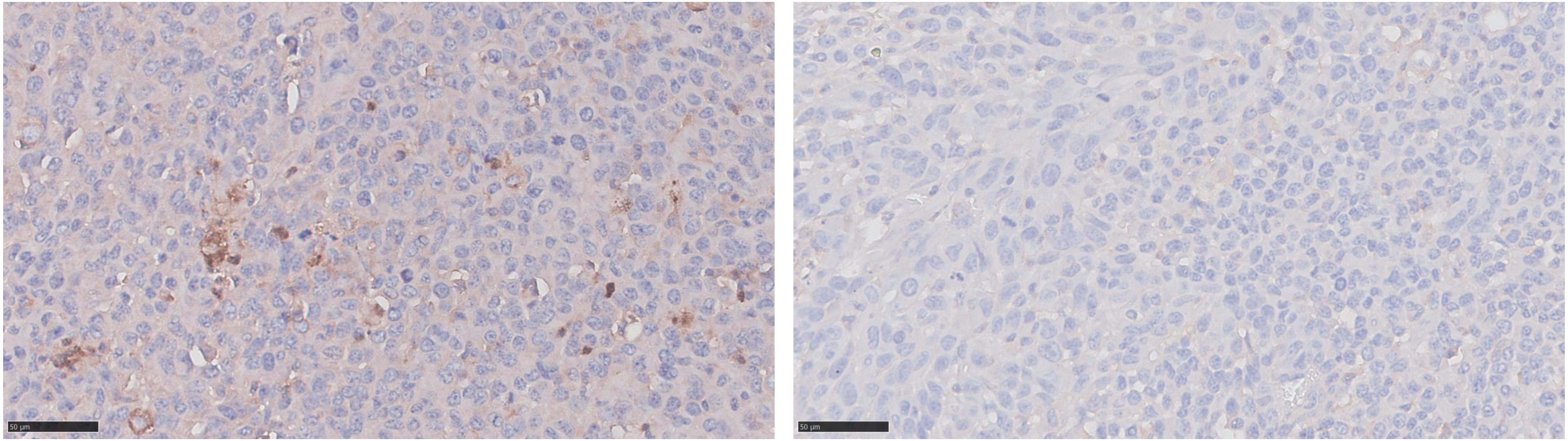

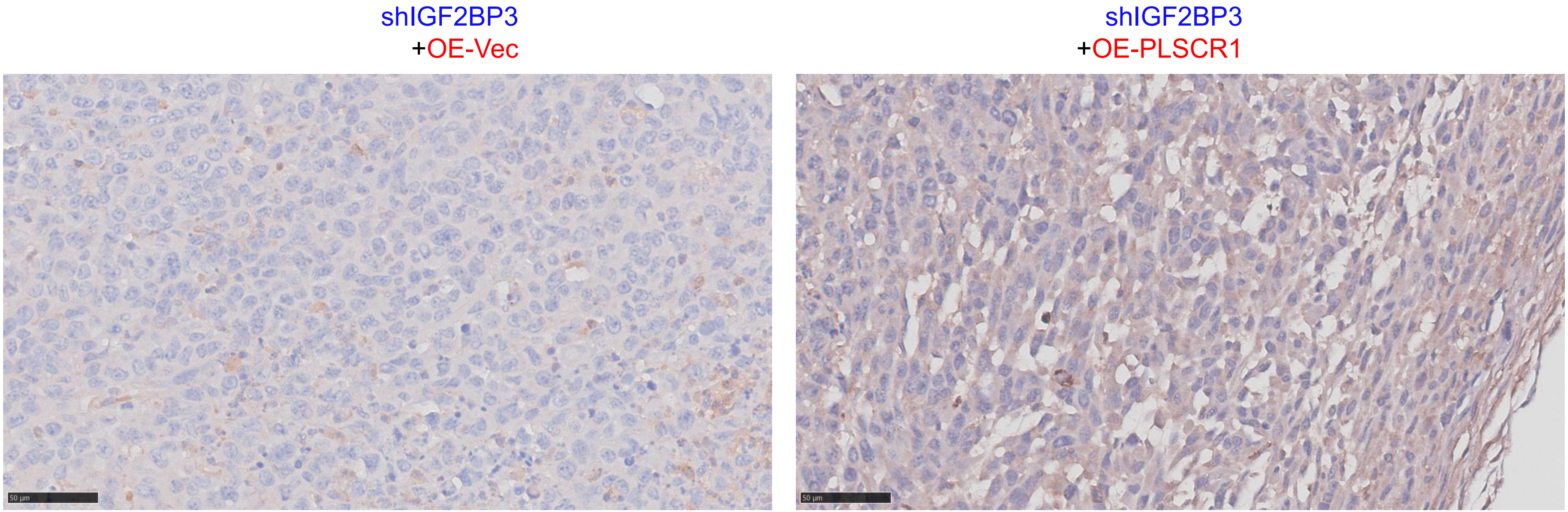
shIGF2BP3
+OE-Vec
shIGF2BP3
+OE-PLSCR1
50 µm
50 µm

Anti-Ki67

shNC

shIGF2BP3

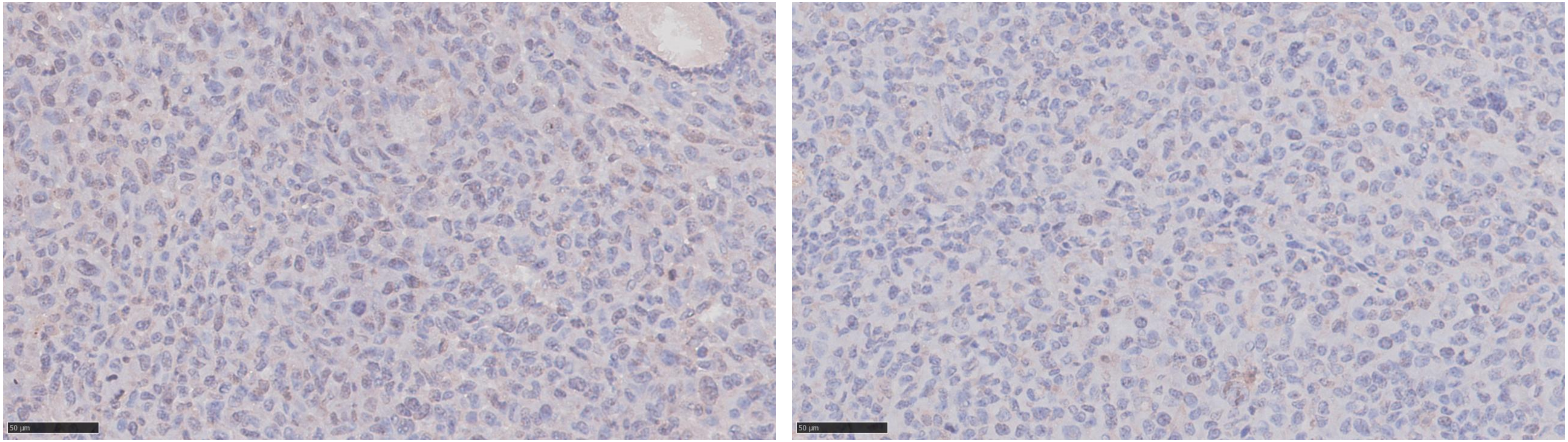

shIGF2BP3
+OE-Vec

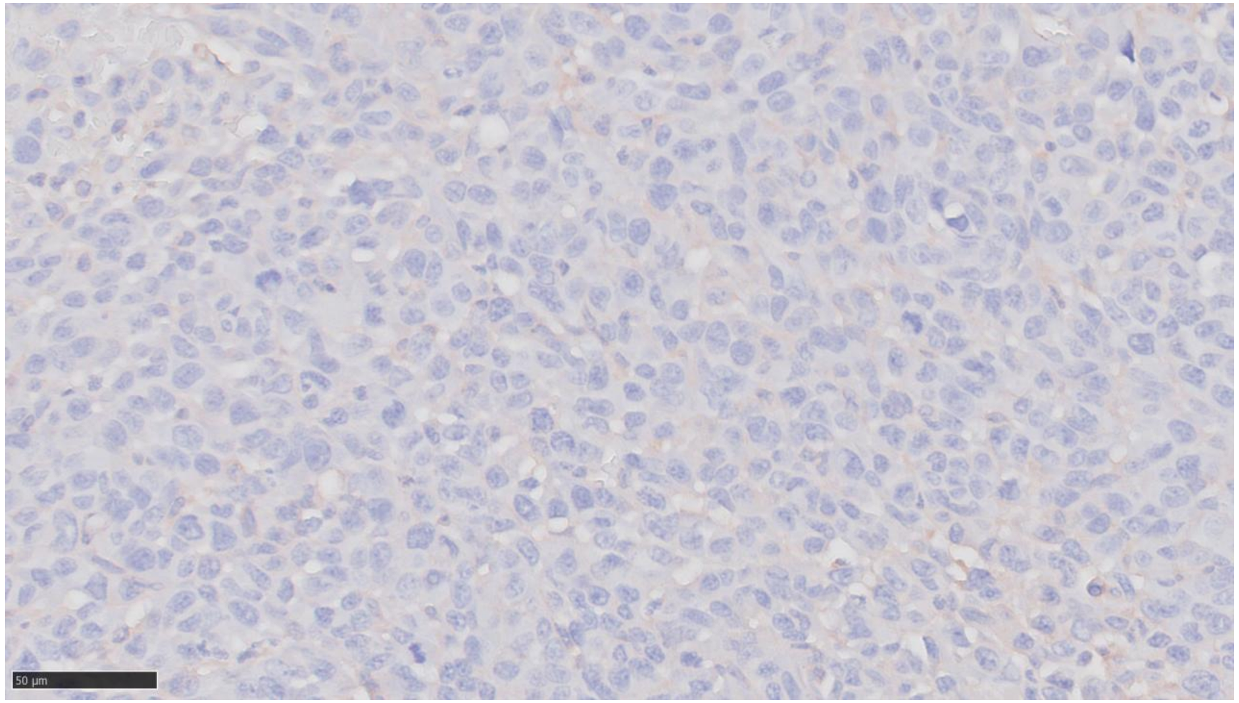

shIGF2BP3
+OE-PLSCR1

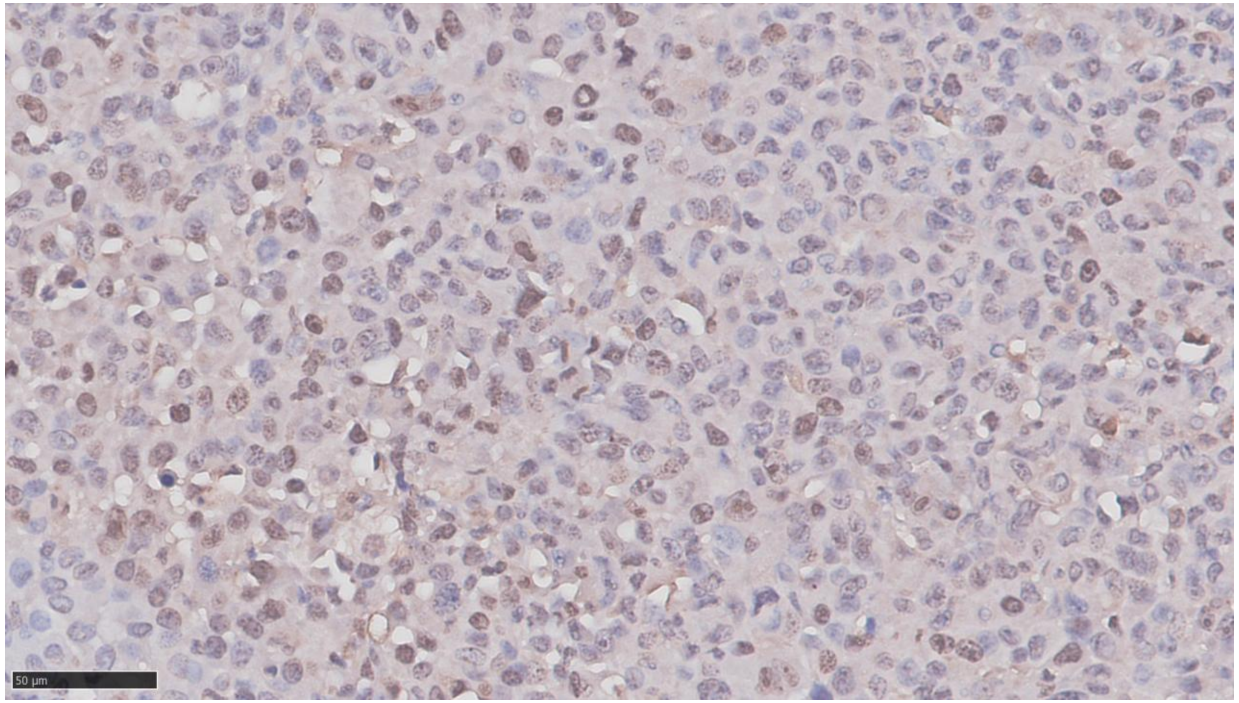

Supplement: Supplementary file 5 — IHC figures [file 41419_2026_8845_MOESM5_ESM.pdf]
