# Supplementary material for: PLSCR1 drives chemoresistance in TNBC via METTL3/IGF2BP3-mediated mRNA stabilization and EGFR-MAPK pathway activation
Source: Cell Death Dis. 2026 May 15;17(1):624. doi: 10.1038/s41419-026-08845-4 (PMC13347015; doi:10.1038/s41419-026-08845-4)
Supplement: Supplementary file 6 — WB unsliced figures [file 41419_2026_8845_MOESM6_ESM.pdf]

Fig S2e

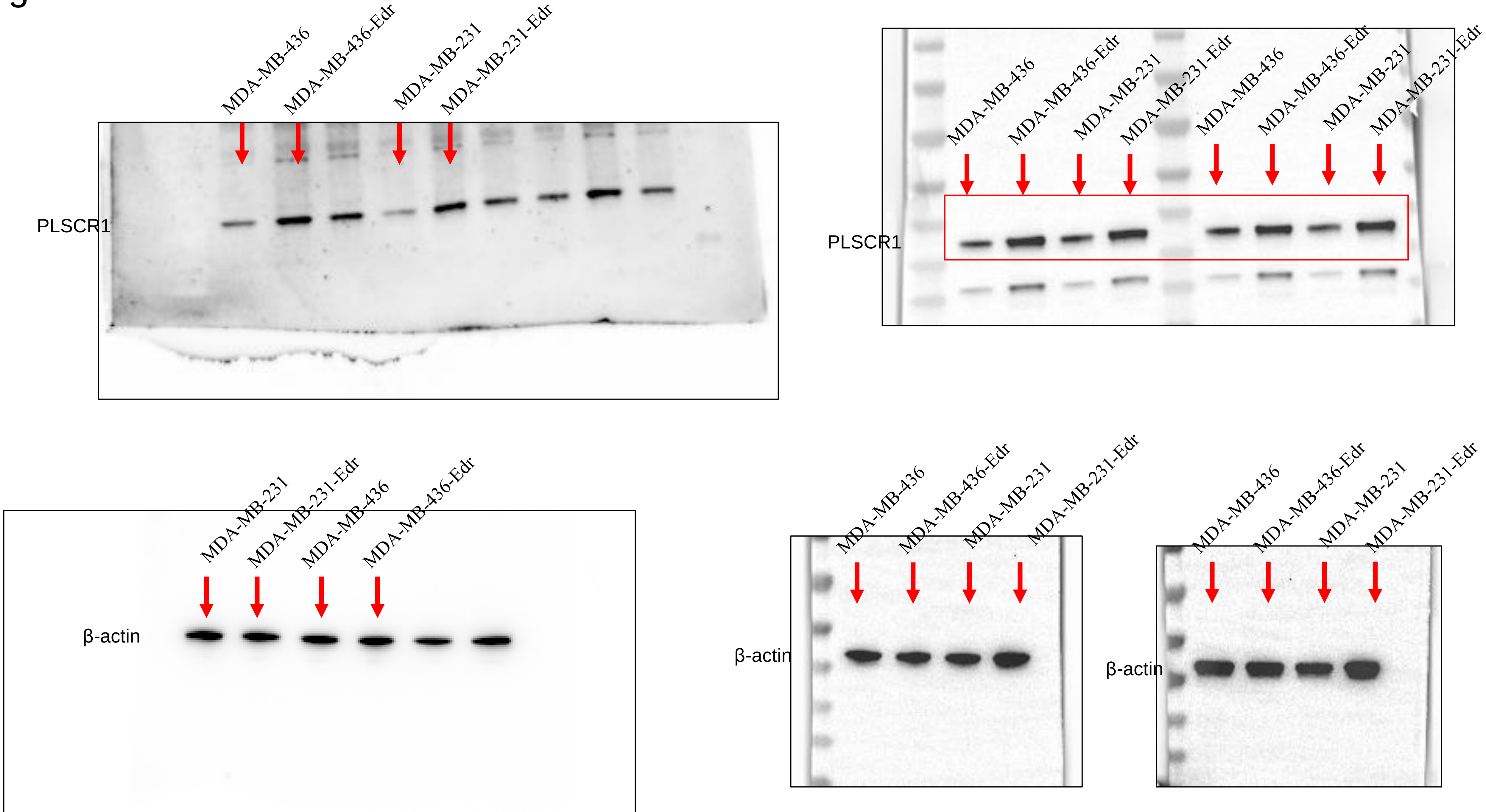
MDA-MB-436
MDA-MB-436-Edr
MDA-MB-231
MDA-MB-231-Edr
PLSCR1
MDA-MB-436
MDA-MB-436-Edr
MDA-MB-231
MDA-MB-231-Edr
MDA-MB-436
MDA-MB-436-Edr
MDA-MB-231
MDA-MB-231-Edr
PLSCR1
MDA-MB-231
MDA-MB-231-Edr
MDA-MB-436
MDA-MB-436-Edr
β-actin
MDA-MB-436
MDA-MB-436-Edr
MDA-MB-231
MDA-MB-231-Edr
β-actin
MDA-MB-436
MDA-MB-436-Edr
MDA-MB-231
MDA-MB-231-Edr
β-actin

Fig S3b

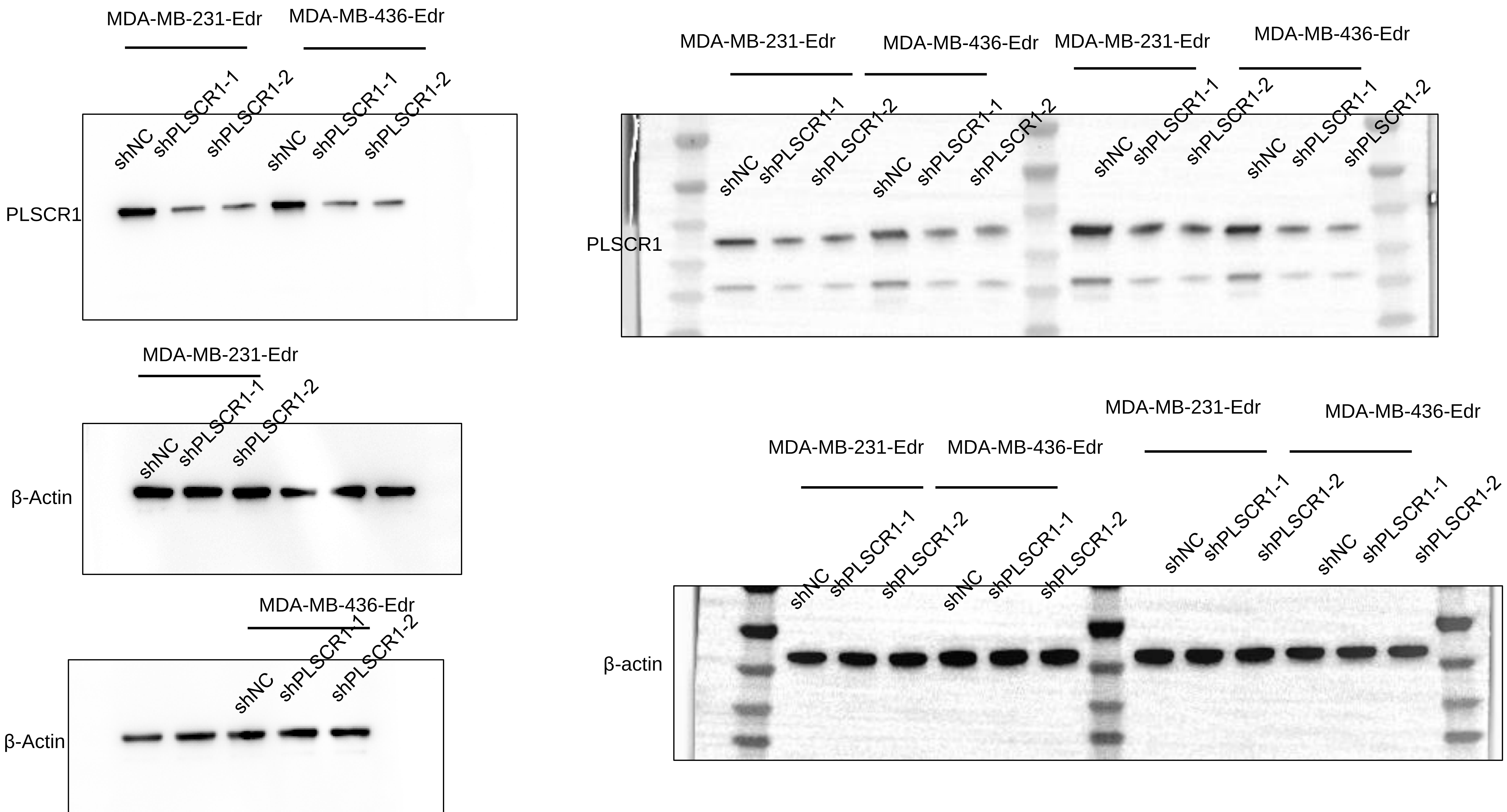
MDA-MB-231-Edr
MDA-MB-436-Edr
shNC
shPLSCR1-1
shPLSCR1-2
shNC
shPLSCR1-1
shPLSCR1-2
PLSCR1
MDA-MB-231-Edr
MDA-MB-436-Edr
MDA-MB-231-Edr
MDA-MB-436-Edr
shNC
shPLSCR1-1
shPLSCR1-2
shNC
shPLSCR1-1
shPLSCR1-2
shNC
shPLSCR1-1
shPLSCR1-2
shNC
shPLSCR1-1
shPLSCR1-2
PLSCR1
MDA-MB-231-Edr
shNC
shPLSCR1-1
shPLSCR1-2
β-Actin
MDA-MB-231-Edr
MDA-MB-436-Edr
MDA-MB-231-Edr
MDA-MB-436-Edr
shNC
shPLSCR1-1
shPLSCR1-2
shNC
shPLSCR1-1
shPLSCR1-2
shNC
shPLSCR1-1
shPLSCR1-2
shNC
shPLSCR1-1
shPLSCR1-2
β-actin
MDA-MB-436-Edr
shNC
shPLSCR1-1
shPLSCR1-2
β-Actin

Fig S3c

c

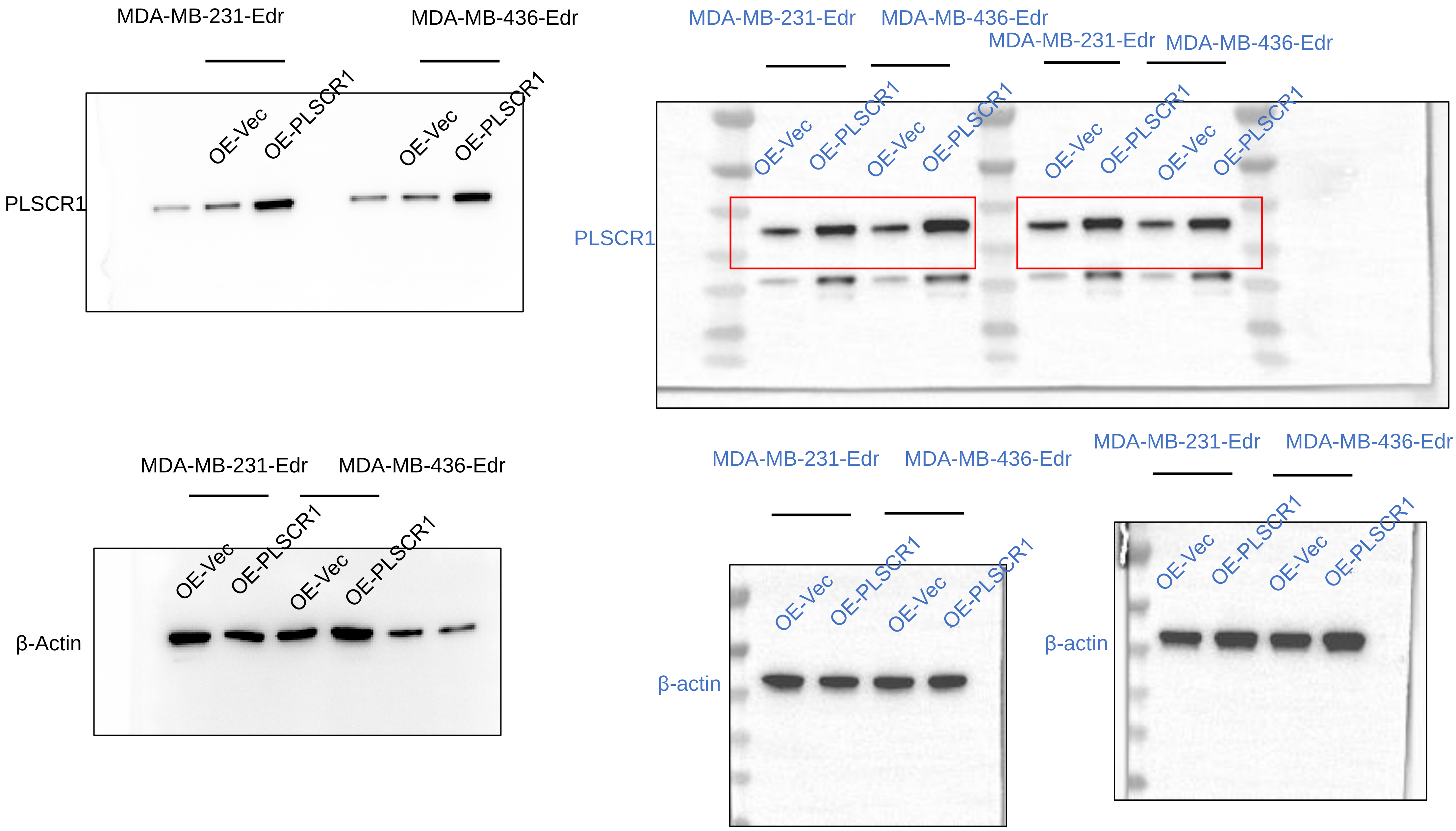
MDA-MB-231-Edr
MDA-MB-436-Edr
OE-Vec
OE-PLSCR1
OE-Vec
OE-PLSCR1
PLSCR1
MDA-MB-231-Edr
MDA-MB-436-Edr
MDA-MB-231-Edr
MDA-MB-436-Edr
OE-Vec
OE-PLSCR1
OE-Vec
OE-PLSCR1
OE-Vec
OE-PLSCR1
OE-Vec
OE-PLSCR1
PLSCR1
MDA-MB-231-Edr
MDA-MB-436-Edr
OE-Vec
OE-PLSCR1
OE-Vec
OE-PLSCR1
β-Actin
MDA-MB-231-Edr
MDA-MB-436-Edr
OE-Vec
OE-PLSCR1
OE-Vec
OE-PLSCR1
β-actin
MDA-MB-231-Edr
MDA-MB-436-Edr
OE-Vec
OE-PLSCR1
OE-Vec
OE-PLSCR1
β-actin

Fig 3

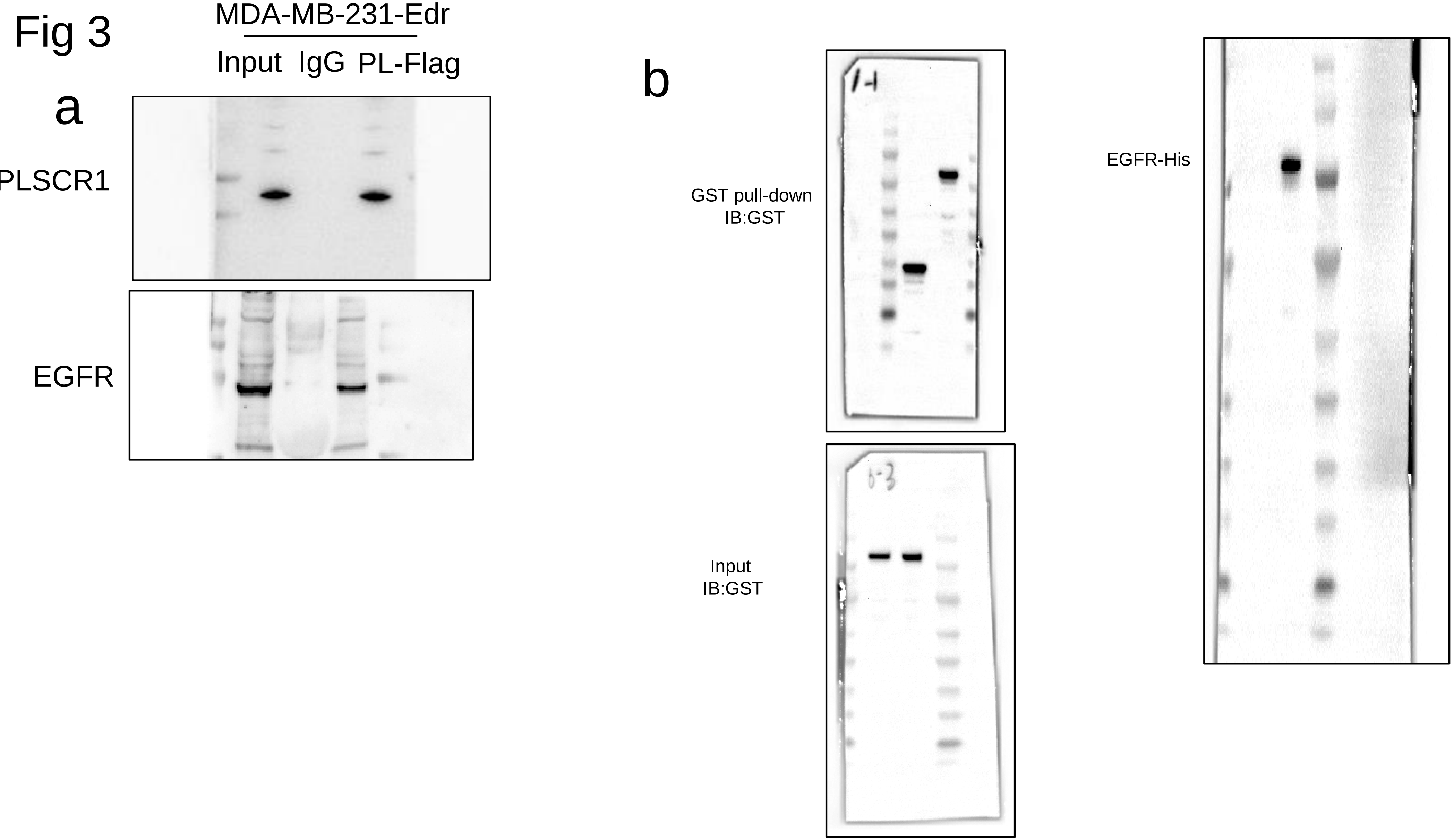
MDA-MB-231-Edr
Input
IgG
PL-Flag
a
PLSCR1
EGFR
b
GST pull-down
IB:GST
Input
IB:GST
EGFR-His

Fig 3e

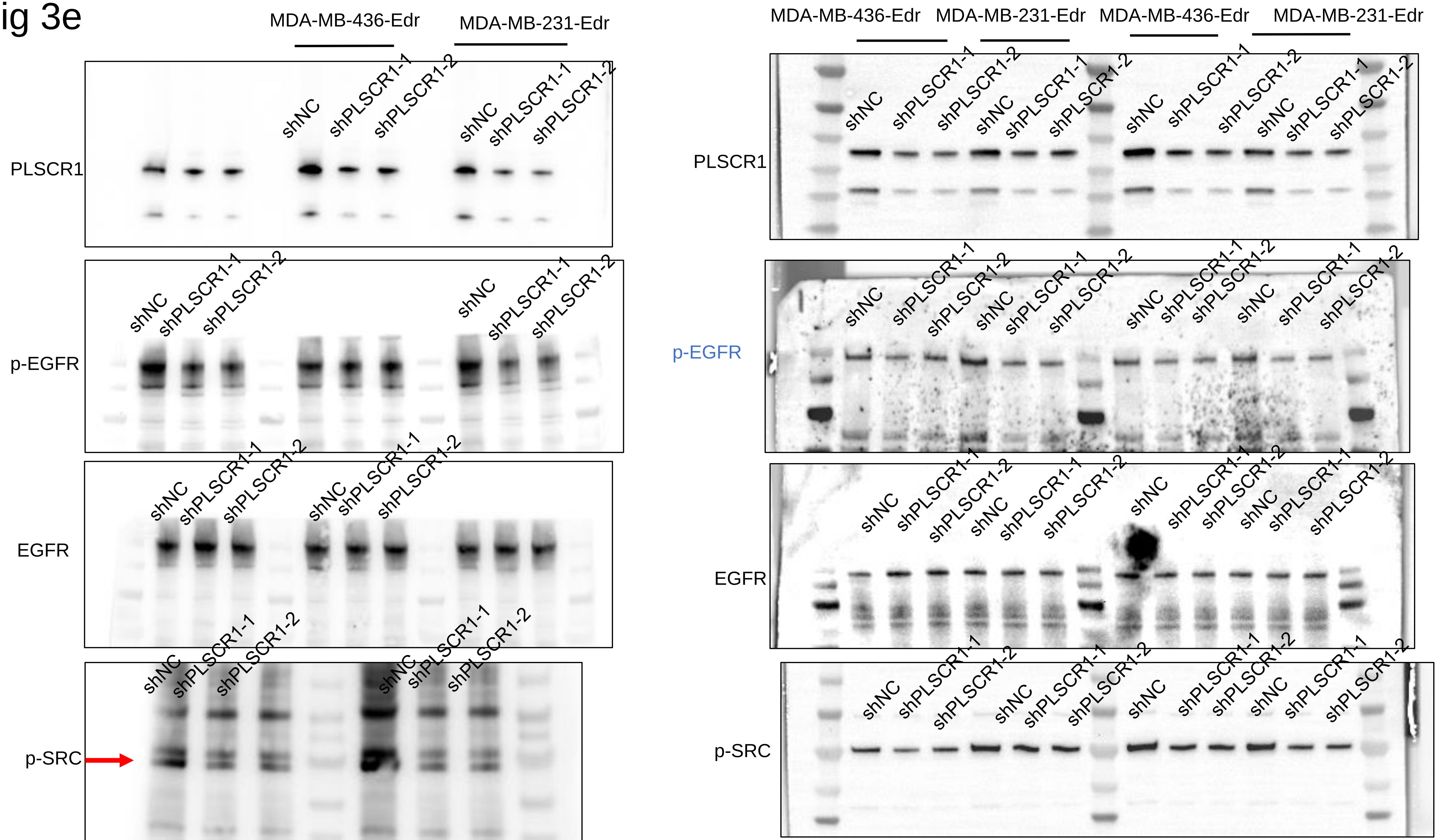

MDA-MB-436-Edr
MDA-MB-231-Edr
shNC
shPLSCR1-1
shPLSCR1-2
PLSCR1
p-EGFR
EGFR
p-SRC

Fig 3e

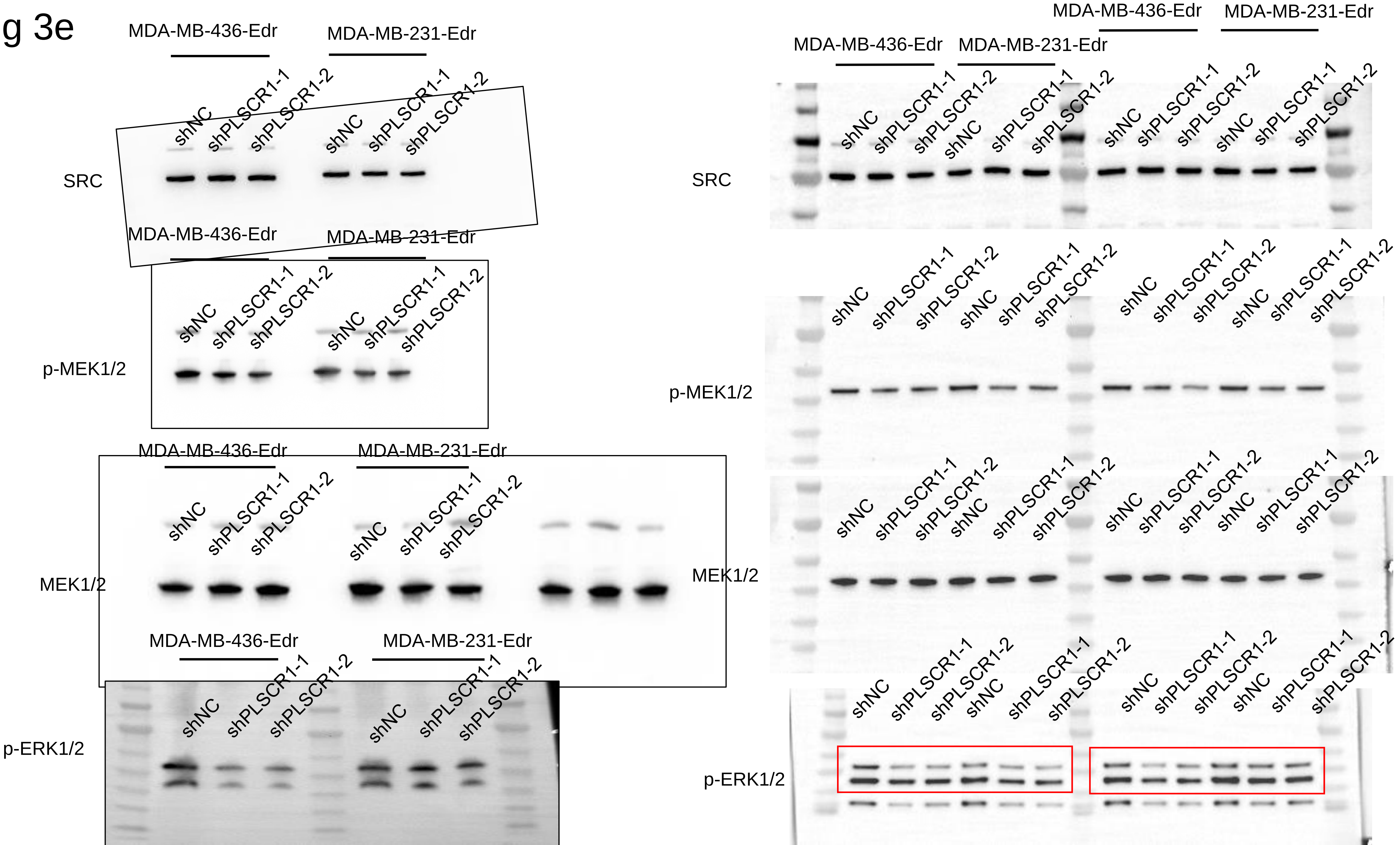
MDA-MB-436-Edr
MDA-MB-231-Edr
shNC
shPLSCR1-1
shPLSCR1-2
SRC
p-MEK1/2
MEK1/2
p-ERK1/2

Fig 3e

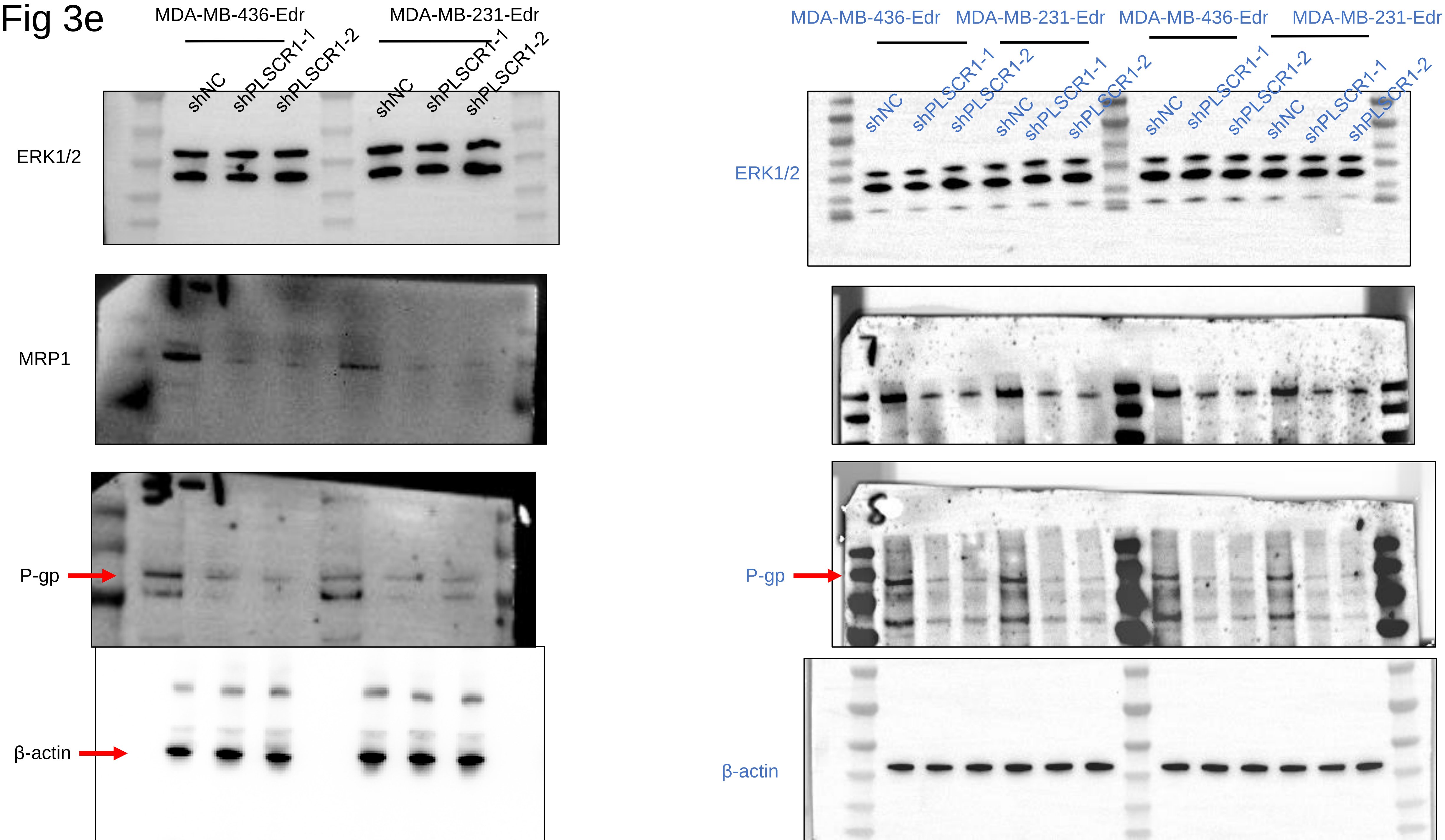
MDA-MB-436-Edr
MDA-MB-231-Edr
shNC
shPLSCR1-1
shPLSCR1-2
shNC
shPLSCR1-1
shPLSCR1-2
ERK1/2
MRP1
P-gp
β-actin
MDA-MB-436-Edr
MDA-MB-231-Edr
MDA-MB-436-Edr
MDA-MB-231-Edr
shNC
shPLSCR1-1
shPLSCR1-2
shNC
shPLSCR1-1
shPLSCR1-2
shNC
shPLSCR1-1
shPLSCR1-2
shNC
shPLSCR1-1
shPLSCR1-2
ERK1/2
P-gp
β-actin

Fig 3g

GST pull-down
IB: His

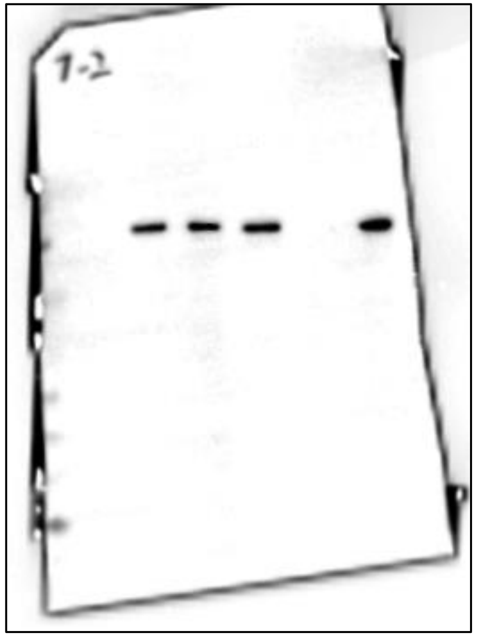

GST pull-down
IB: GST

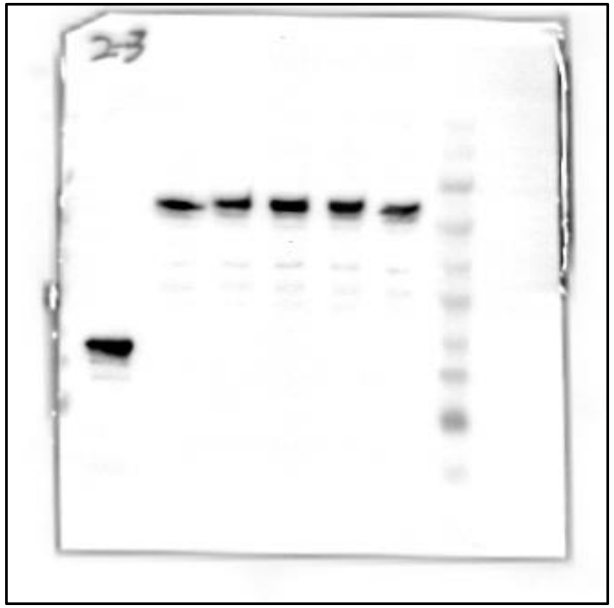

Input
IB: His

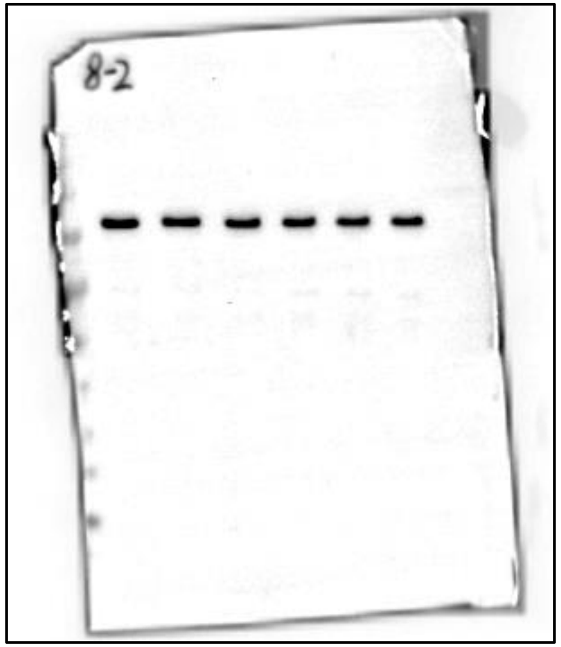

Fig S5

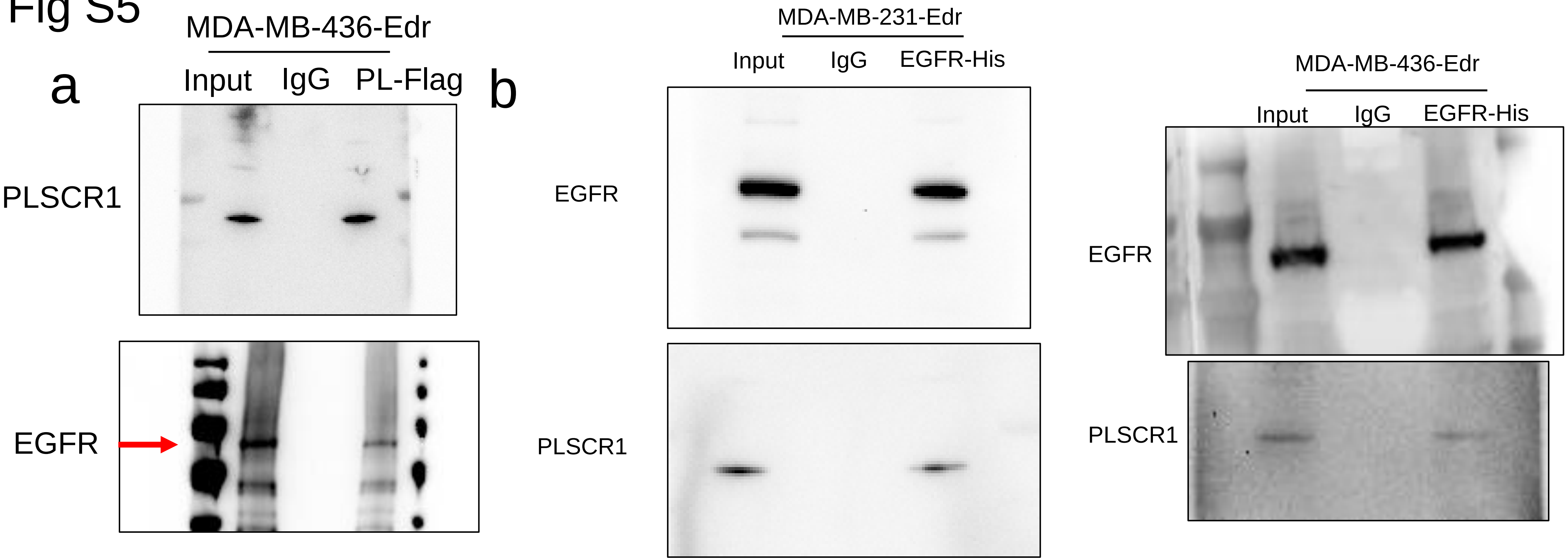
a
MDA-MB-436-Edr
Input
IgG
PL-Flag
PLSCR1
EGFR
b
MDA-MB-231-Edr
Input
IgG
EGFR-His
EGFR
PLSCR1
MDA-MB-436-Edr
Input
IgG
EGFR-His
EGFR
PLSCR1

Fig S5c

GST pull-down
IB: His

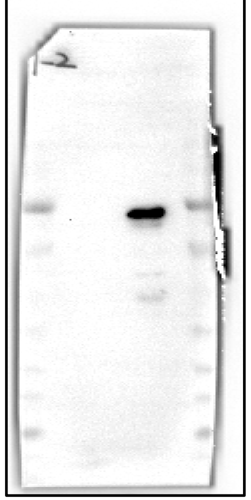

GST pull-down
IB: GST

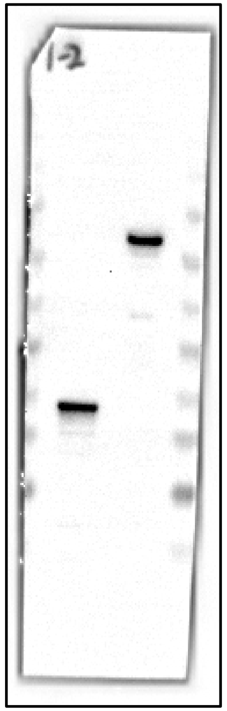

Input
IB: His

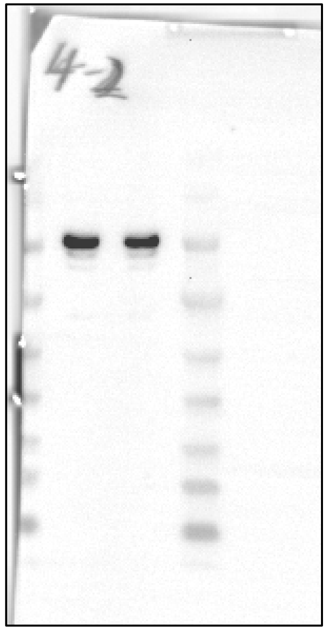

Fig S5f

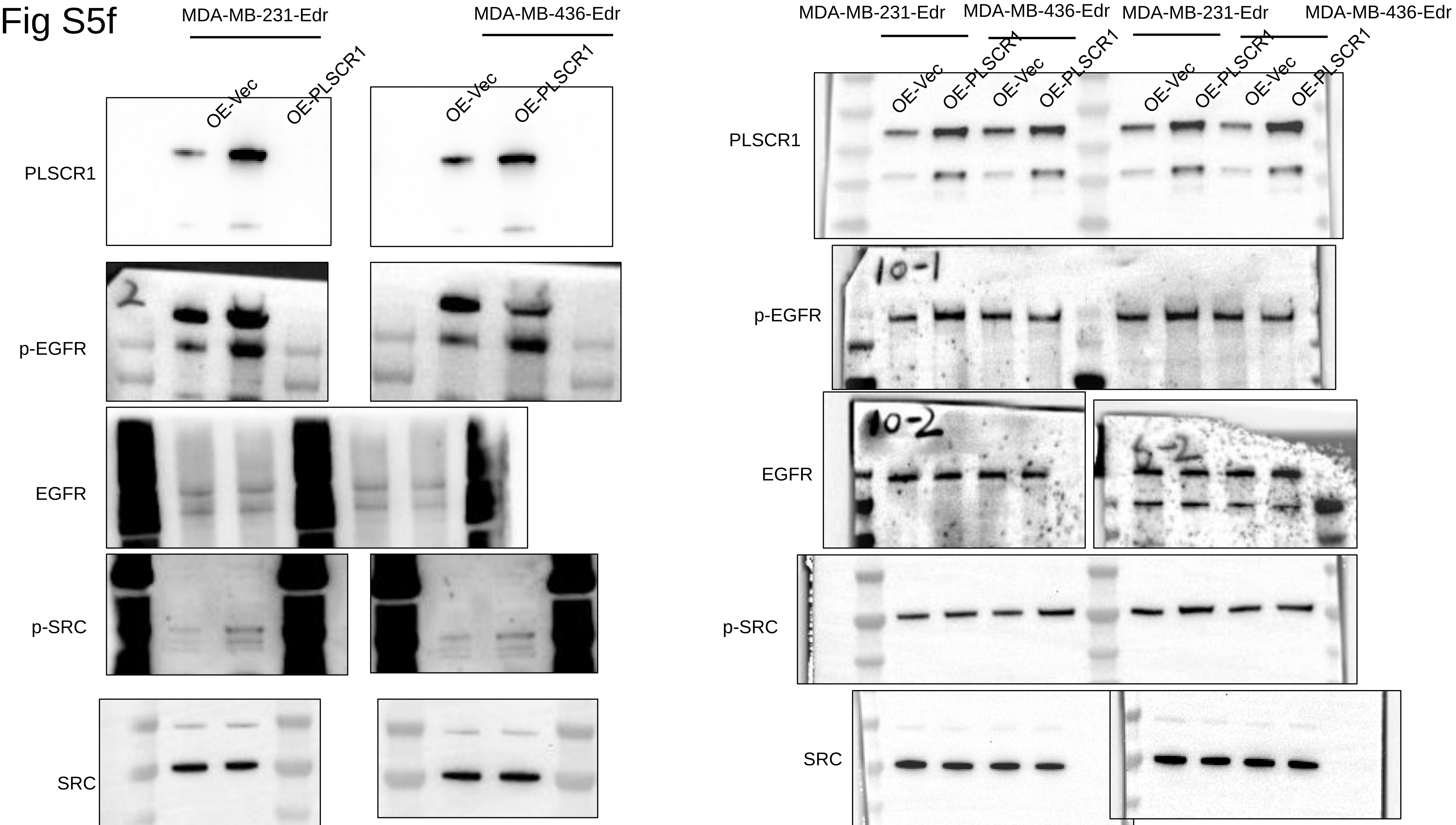
MDA-MB-231-Edr
MDA-MB-436-Edr
OE-Vec
OE-PLSCR1
OE-Vec
OE-PLSCR1
PLSCR1
p-EGFR
EGFR
p-SRC
SRC
MDA-MB-231-Edr
MDA-MB-436-Edr
MDA-MB-231-Edr
MDA-MB-436-Edr
OE-Vec
OE-PLSCR1
OE-Vec
OE-PLSCR1
OE-Vec
OE-PLSCR1
OE-Vec
OE-PLSCR1
PLSCR1
10-1
p-EGFR
10-2
EGFR
p-SRC
SRC

Fig S5f

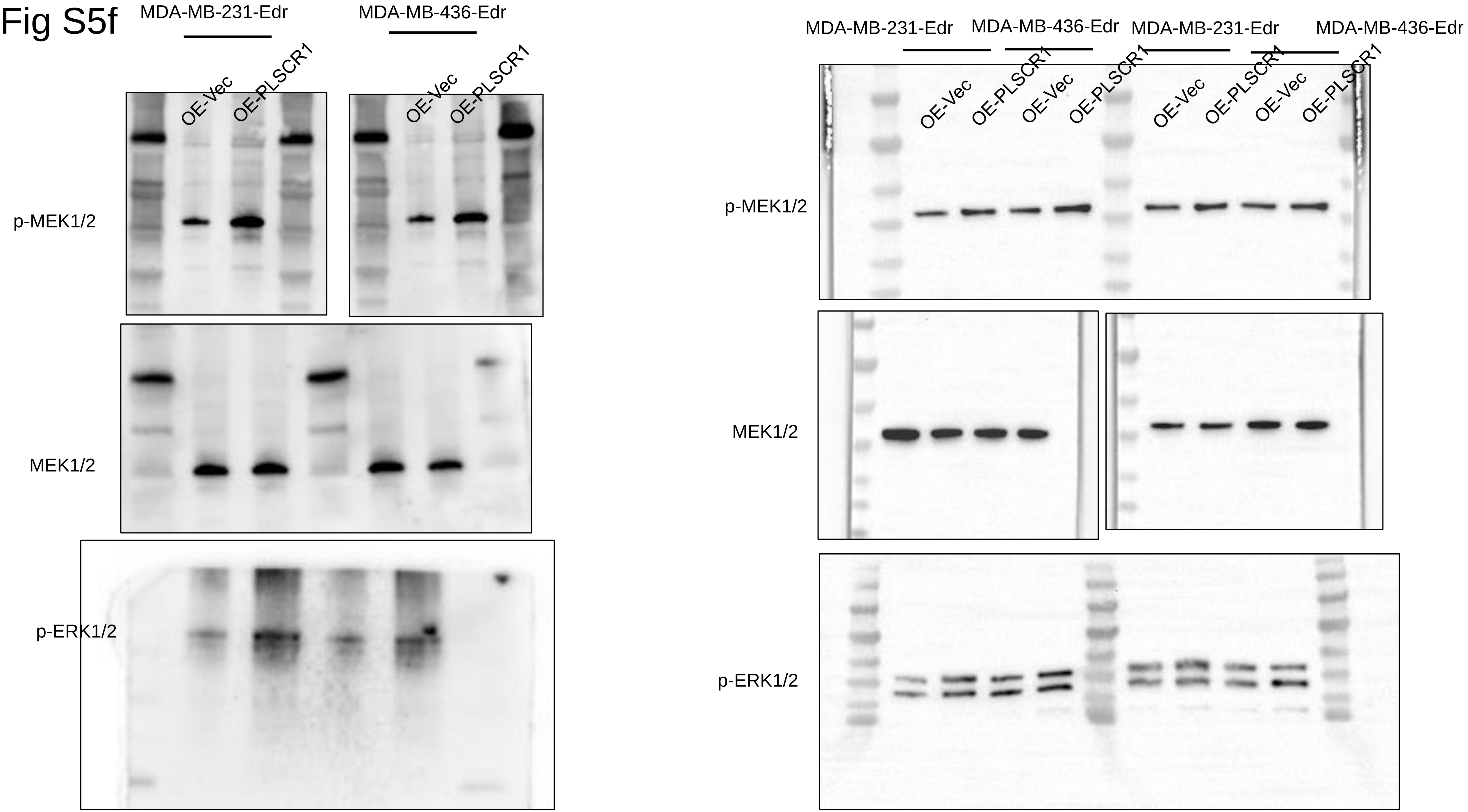

MDA-MB-231-Edr
MDA-MB-436-Edr
OE-Vec
OE-PLSCR1
OE-Vec
OE-PLSCR1
p-MEK1/2
MEK1/2
p-ERK1/2
MDA-MB-231-Edr
MDA-MB-436-Edr
MDA-MB-231-Edr
MDA-MB-436-Edr
OE-Vec
OE-PLSCR1
OE-Vec
OE-PLSCR1
OE-Vec
OE-PLSCR1
OE-Vec
OE-PLSCR1
p-MEK1/2
MEK1/2
p-ERK1/2

Fig S5f

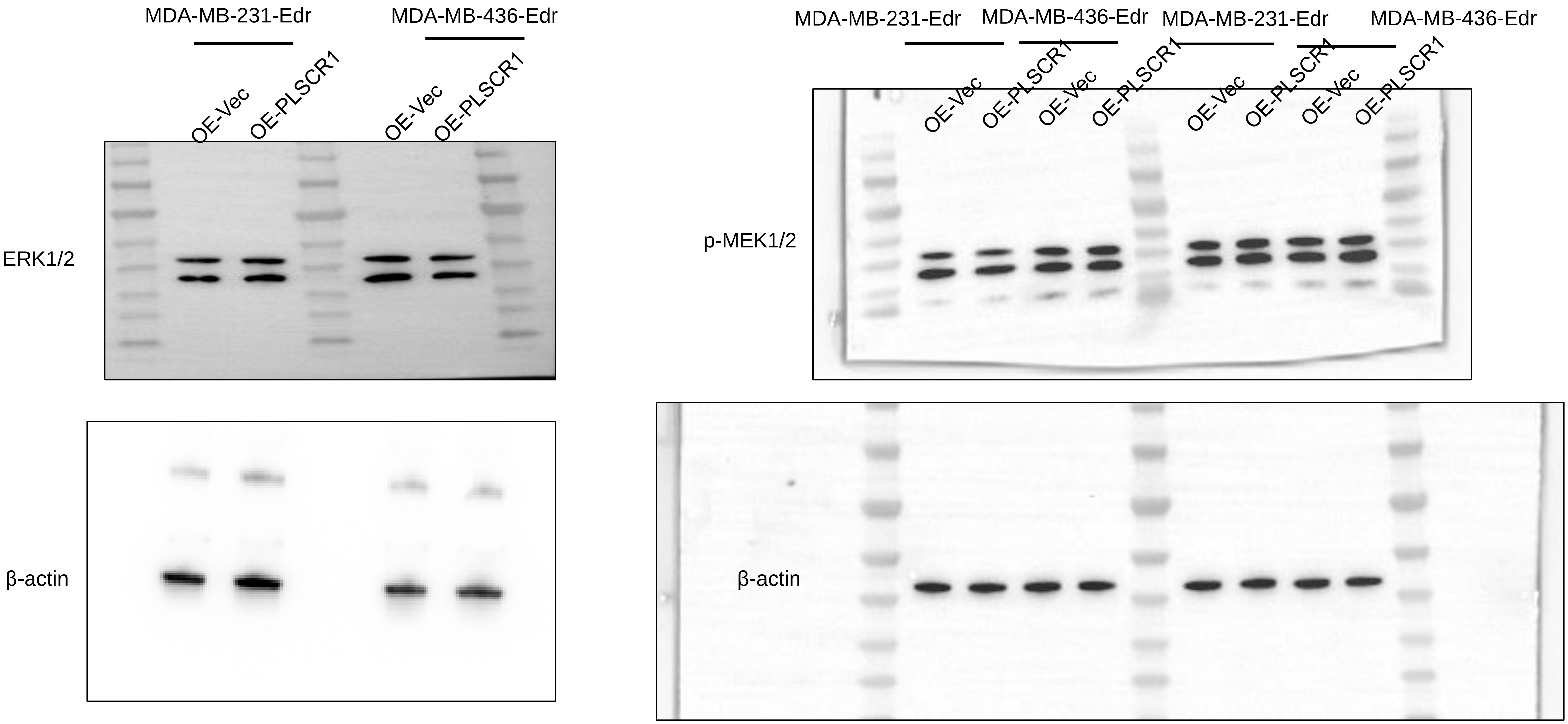
MDA-MB-231-Edr
MDA-MB-436-Edr
OE-Vec
OE-PLSCR1
OE-Vec
OE-PLSCR1
ERK1/2
β-actin
MDA-MB-231-Edr
MDA-MB-436-Edr
MDA-MB-231-Edr
MDA-MB-436-Edr
OE-Vec
OE-PLSCR1
OE-Vec
OE-PLSCR1
OE-Vec
OE-PLSCR1
OE-Vec
OE-PLSCR1
p-MEK1/2
β-actin

Fig S6

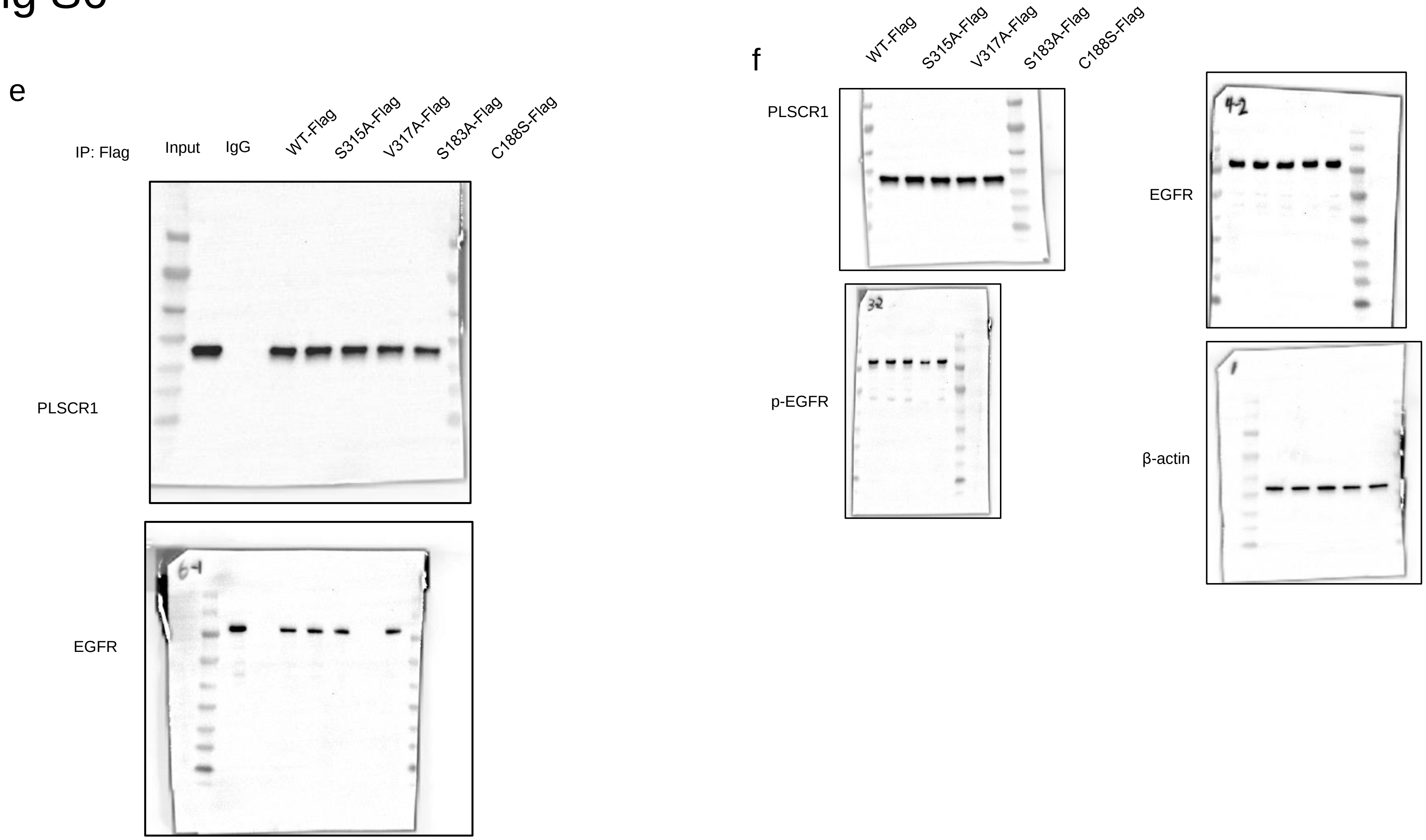
e
IP: Flag
Input
IgG
WT-Flag
S315A-Flag
V317A-Flag
S183A-Flag
C188S-Flag
PLSCR1
EGFR
f
WT-Flag
S315A-Flag
V317A-Flag
S183A-Flag
C188S-Flag
PLSCR1
p-EGFR
EGFR
β-actin

Fig 4e

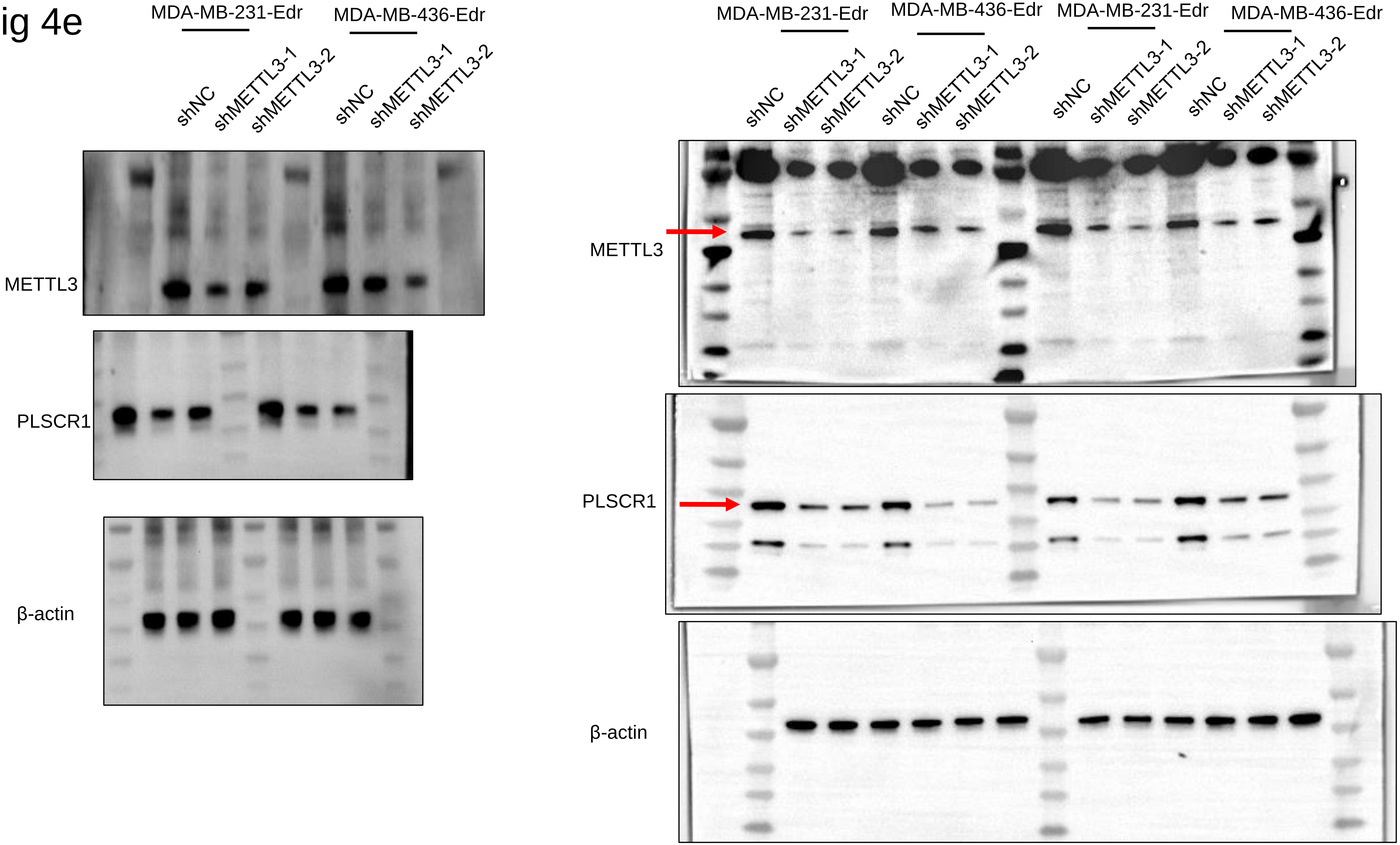

MDA-MB-231-Edr
MDA-MB-436-Edr
shNC
shMETTL3-1
shMETTL3-2
METTL3
PLSCR1
β-actin

Fig 5c

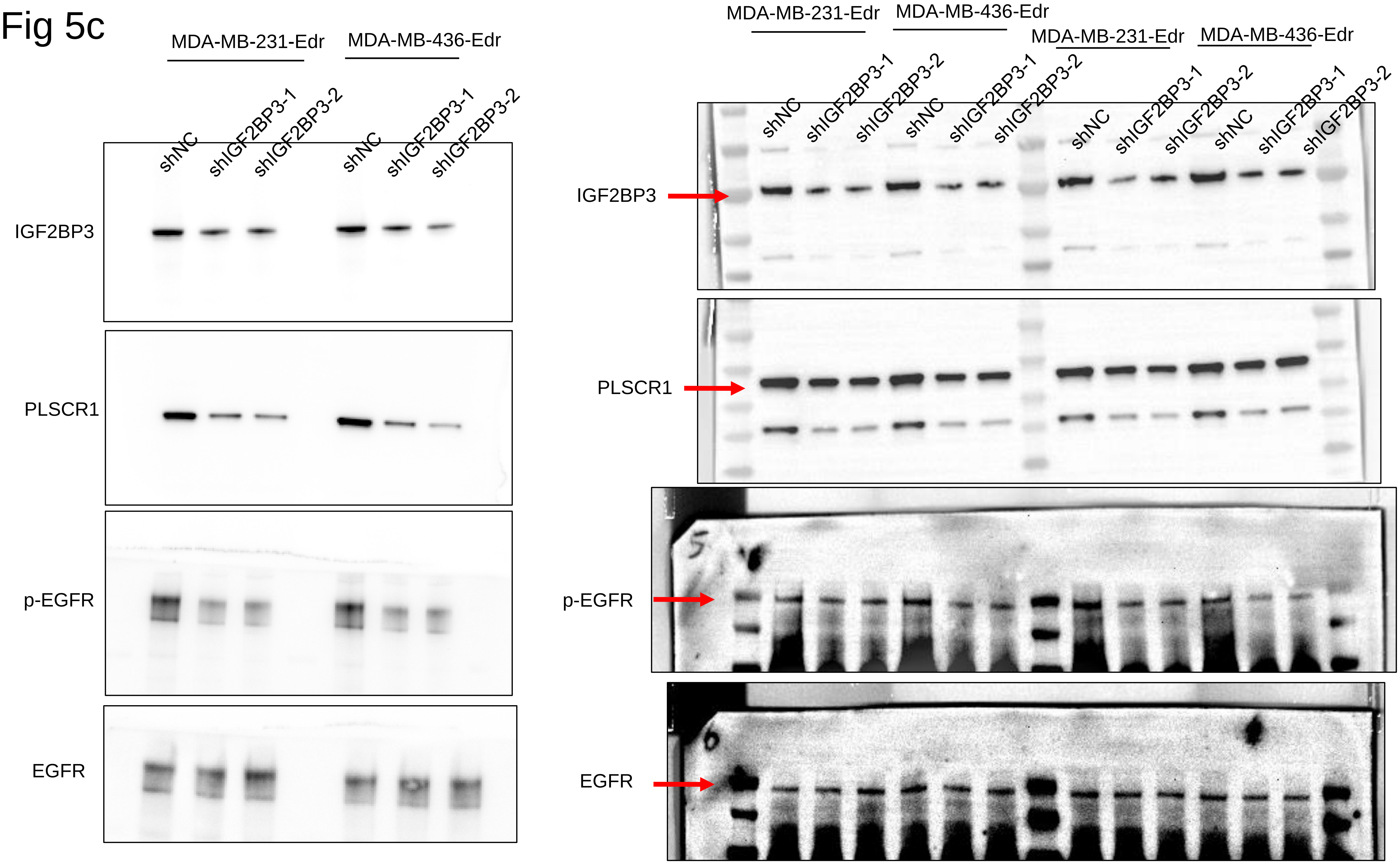
MDA-MB-231-Edr
MDA-MB-436-Edr
shNC
shIGF2BP3-1
shIGF2BP3-2
IGF2BP3
PLSCR1
p-EGFR
EGFR
5
6

Fig 5c

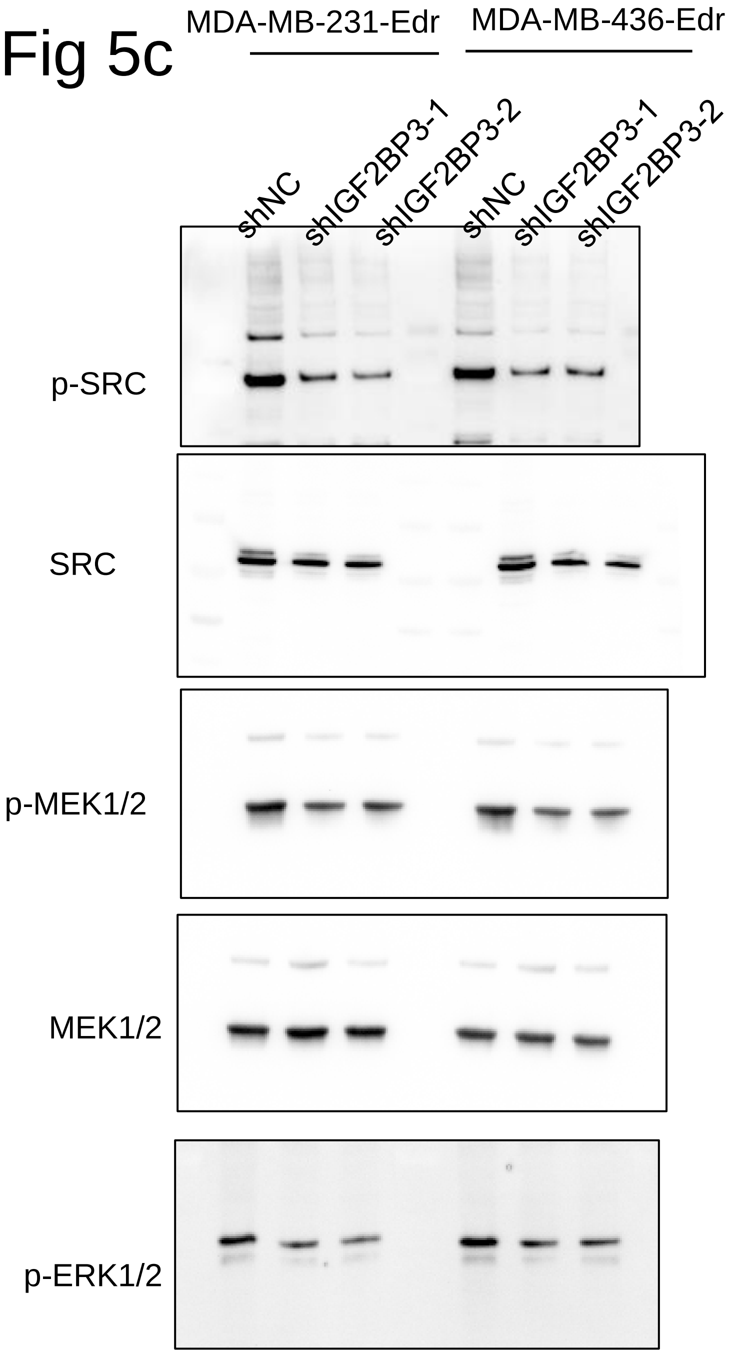

MDA-MB-231-Edr
MDA-MB-436-Edr
shNC
shIGF2BP3-1
shIGF2BP3-2
shNC
shIGF2BP3-1
shIGF2BP3-2
p-SRC
SRC
p-MEK1/2
MEK1/2
p-ERK1/2

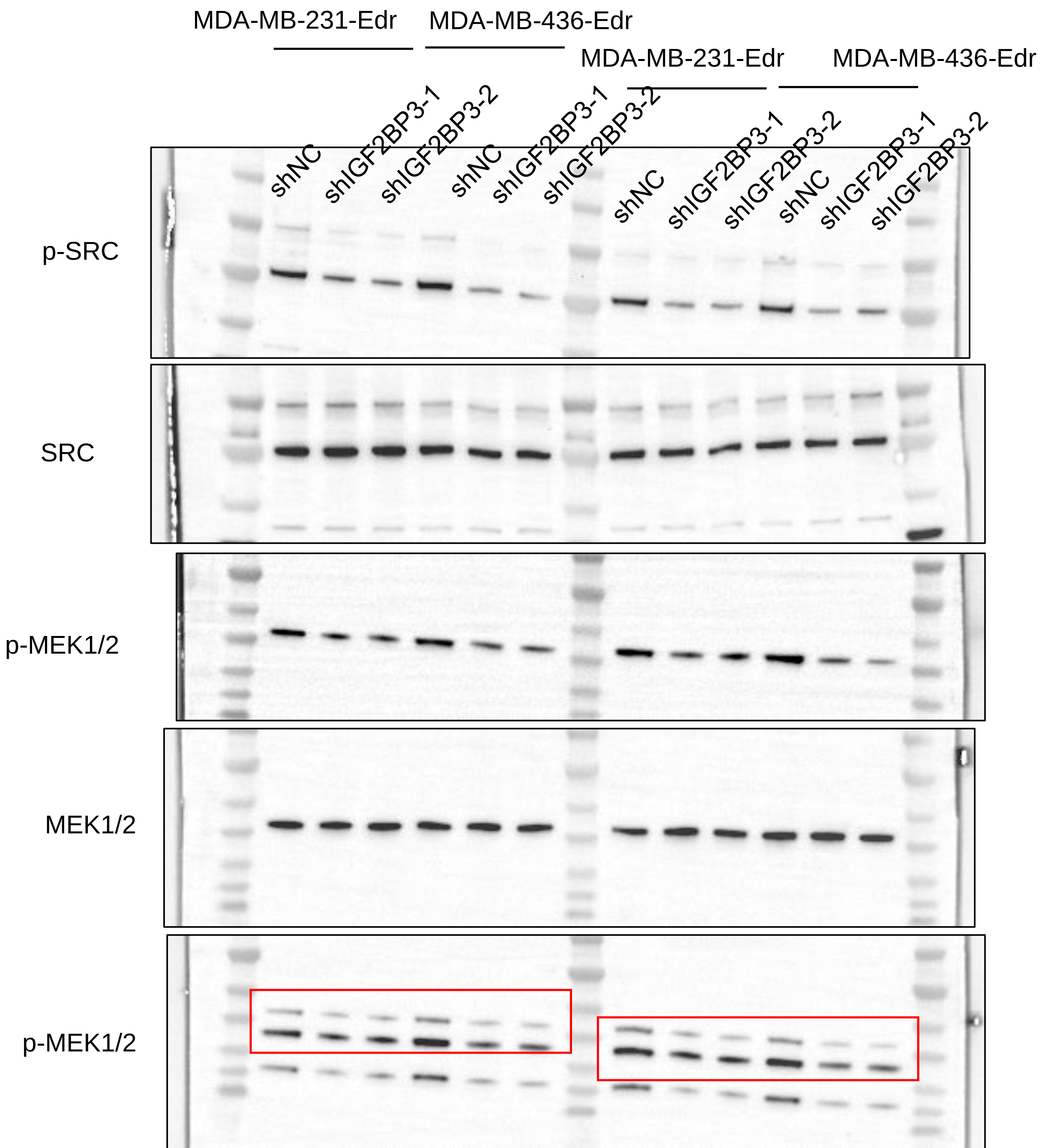

MDA-MB-231-Edr
MDA-MB-436-Edr
MDA-MB-231-Edr
MDA-MB-436-Edr
shNC
shIGF2BP3-1
shIGF2BP3-2
shNC
shIGF2BP3-1
shIGF2BP3-2
shNC
shIGF2BP3-1
shIGF2BP3-2
shNC
shIGF2BP3-1
shIGF2BP3-2
p-SRC
SRC
p-MEK1/2
MEK1/2
p-MEK1/2

Fig 5c

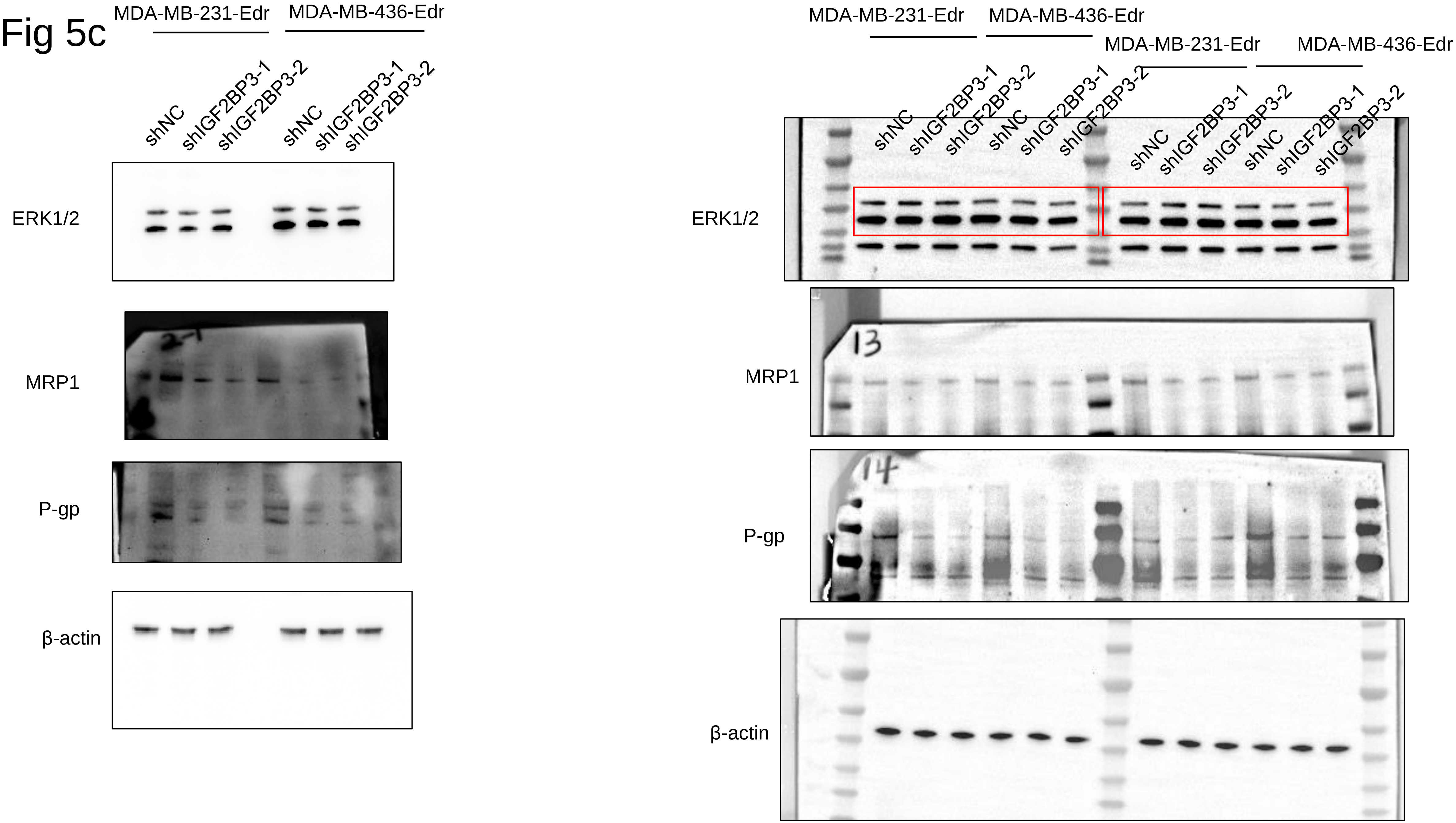
MDA-MB-231-Edr
MDA-MB-436-Edr
shNC
shIGF2BP3-1
shIGF2BP3-2
shNC
shIGF2BP3-1
shIGF2BP3-2
ERK1/2
MRP1
P-gp
β-actin
MDA-MB-231-Edr
MDA-MB-436-Edr
MDA-MB-231-Edr
MDA-MB-436-Edr
shNC
shIGF2BP3-1
shIGF2BP3-2
shNC
shIGF2BP3-1
shIGF2BP3-2
shNC
shIGF2BP3-1
shIGF2BP3-2
shNC
shIGF2BP3-1
shIGF2BP3-2
ERK1/2
13
MRP1
14
P-gp
β-actin

Fig S7e

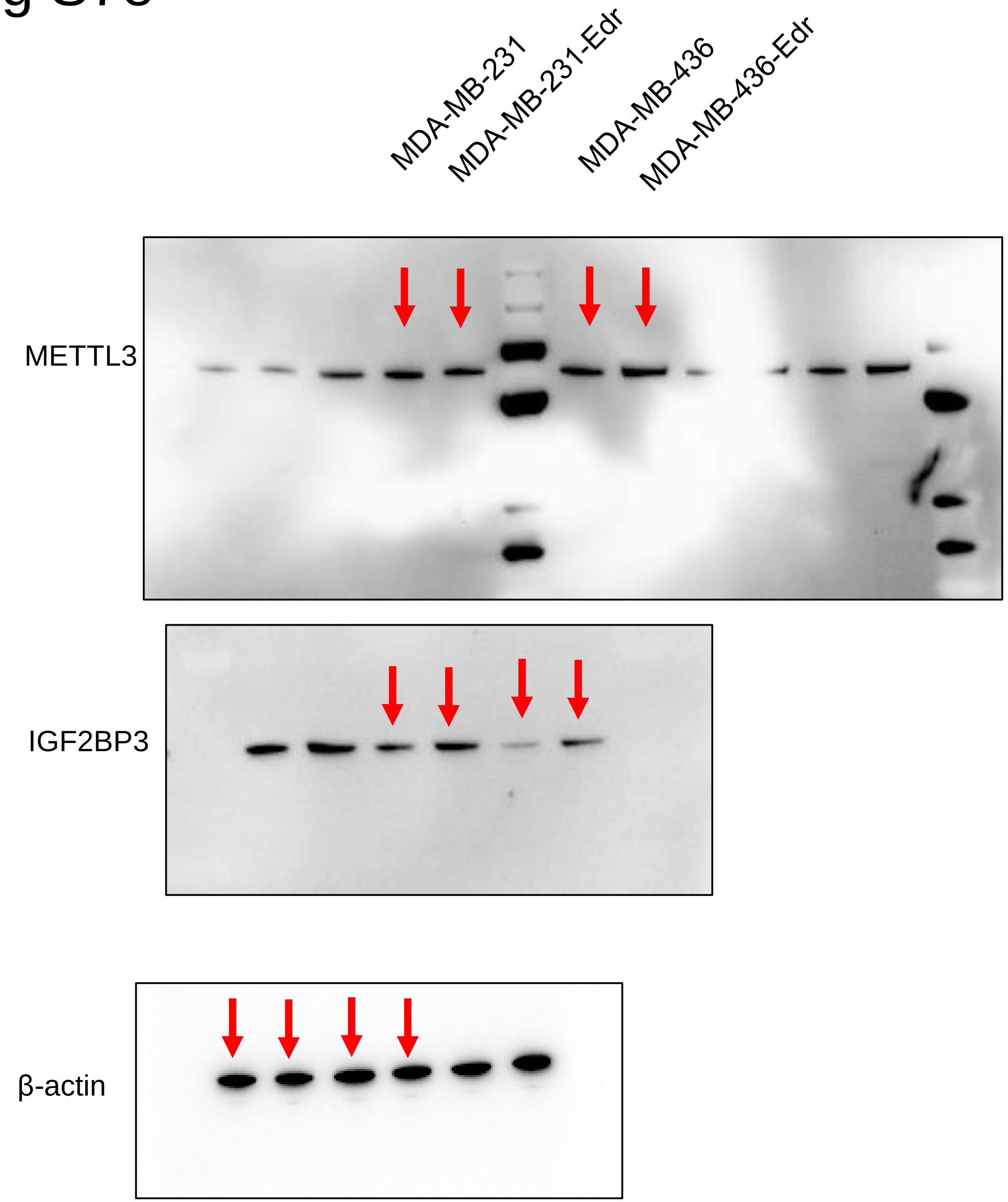
MDA-MB-231
MDA-MB-231-Edr
MDA-MB-436
MDA-MB-436-Edr
METTL3
IGF2BP3
β-actin

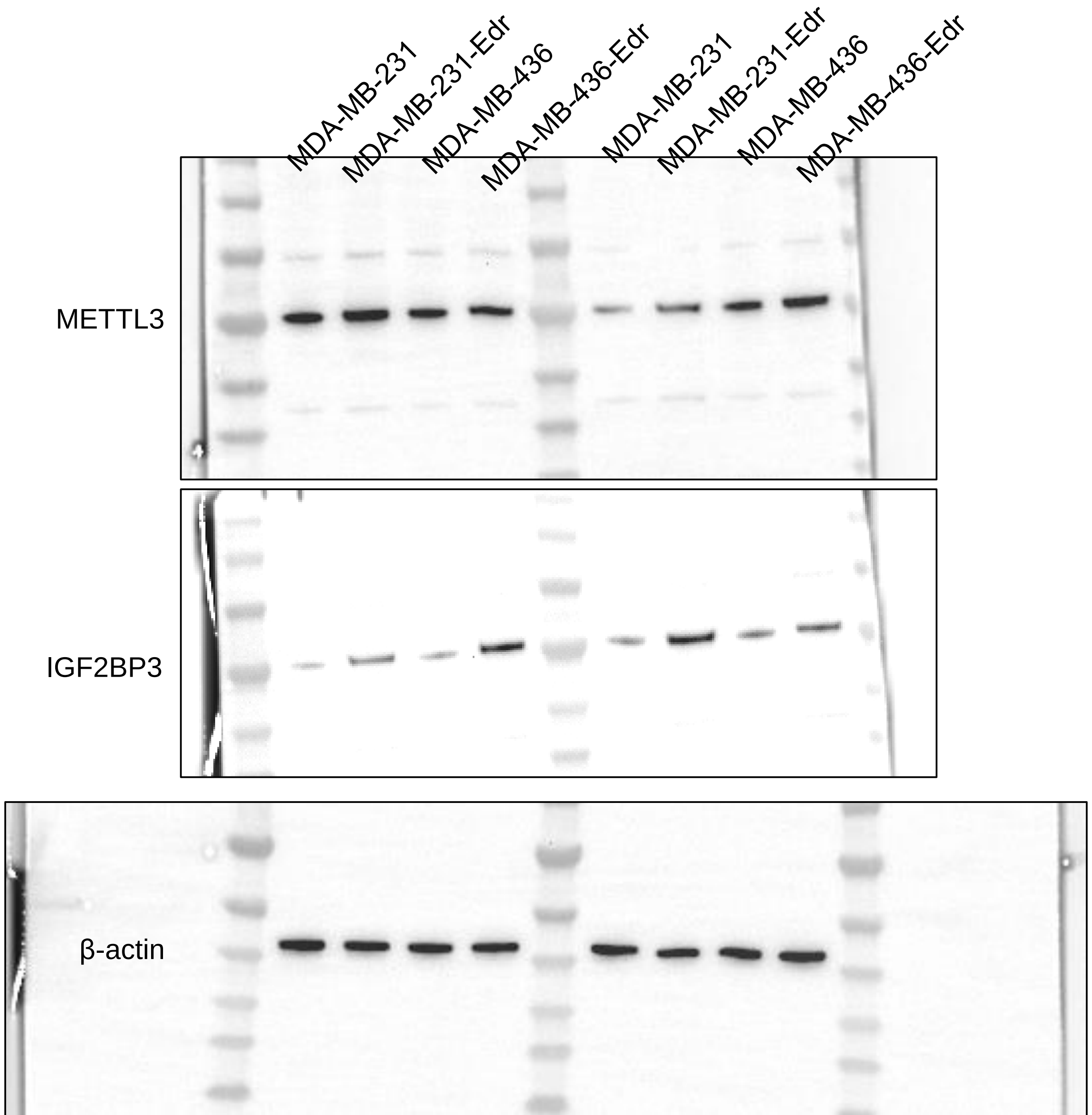
MDA-MB-231
MDA-MB-231-Edr
MDA-MB-436
MDA-MB-436-Edr
MDA-MB-231
MDA-MB-231-Edr
MDA-MB-436
MDA-MB-436-Edr
METTL3
IGF2BP3
β-actin

Fig S8e

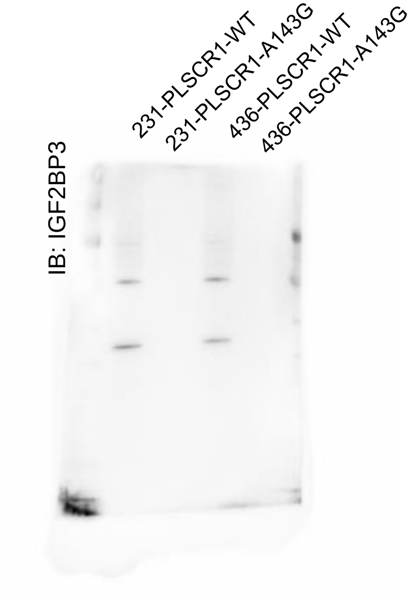
231-PLSCR1-WT
231-PLSCR1-A143G
436-PLSCR1-WT
436-PLSCR1-A143G
IB: IGF2BP3

Fig 6a

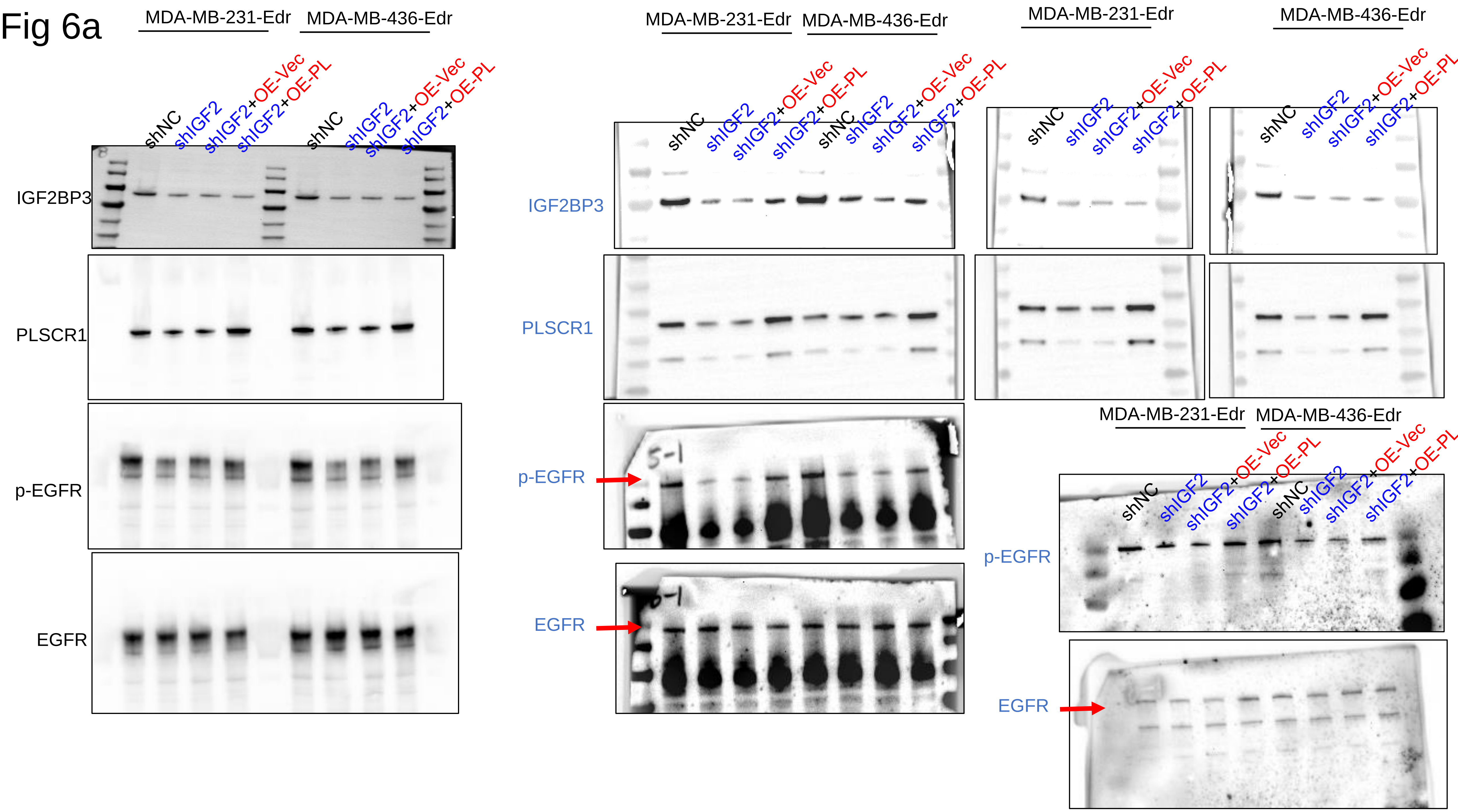
MDA-MB-231-Edr
MDA-MB-436-Edr
shNC
shIGF2
shIGF2+OE-Vec
shIGF2+OE-PL
IGF2BP3
PLSCR1
p-EGFR
EGFR
5-1
6-1

Fig 6a

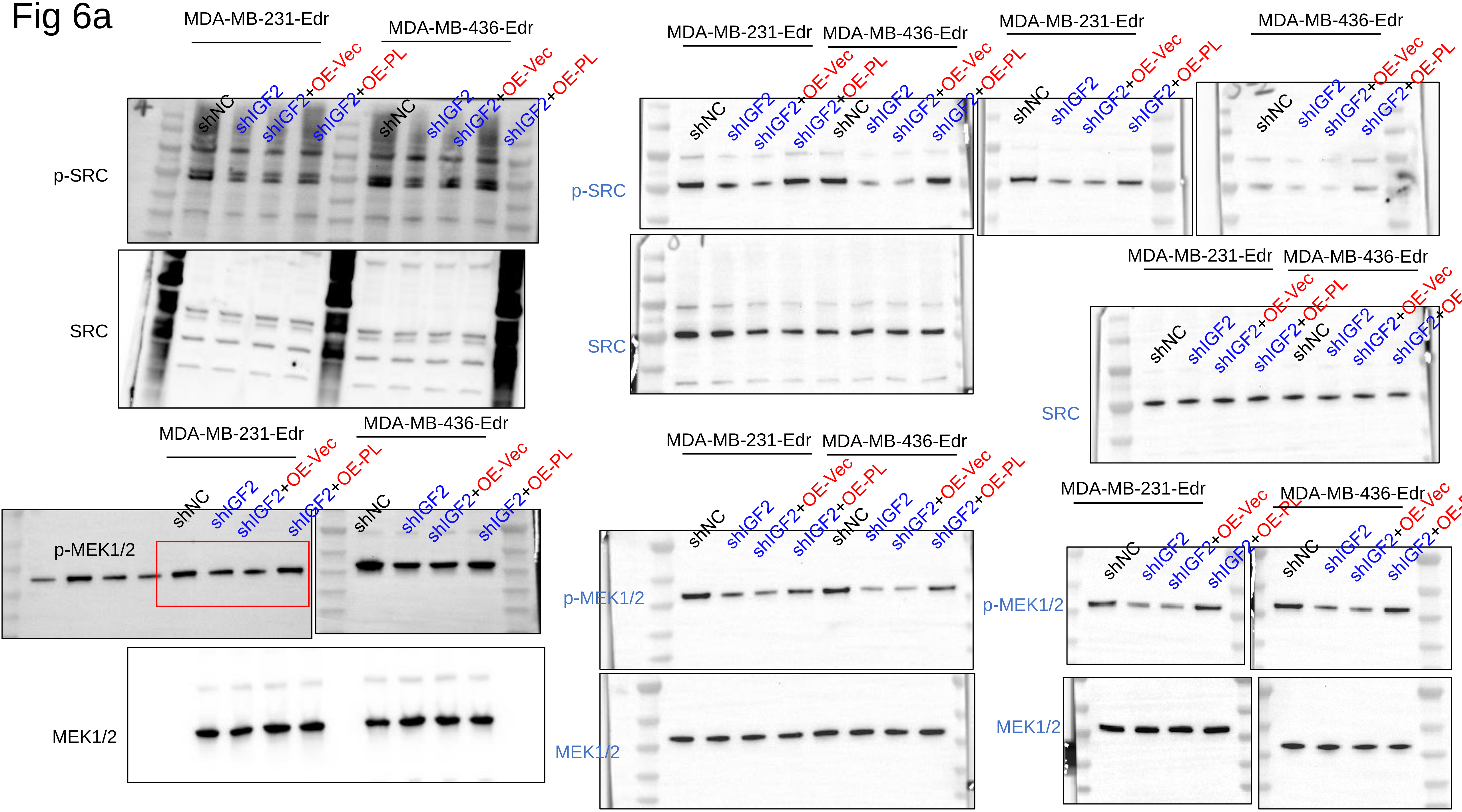
MDA-MB-231-Edr
MDA-MB-436-Edr
shNC
shIGF2
shIGF2+OE-Vec
shIGF2+OE-PL
p-SRC
SRC
p-MEK1/2
MEK1/2

Fig 6a

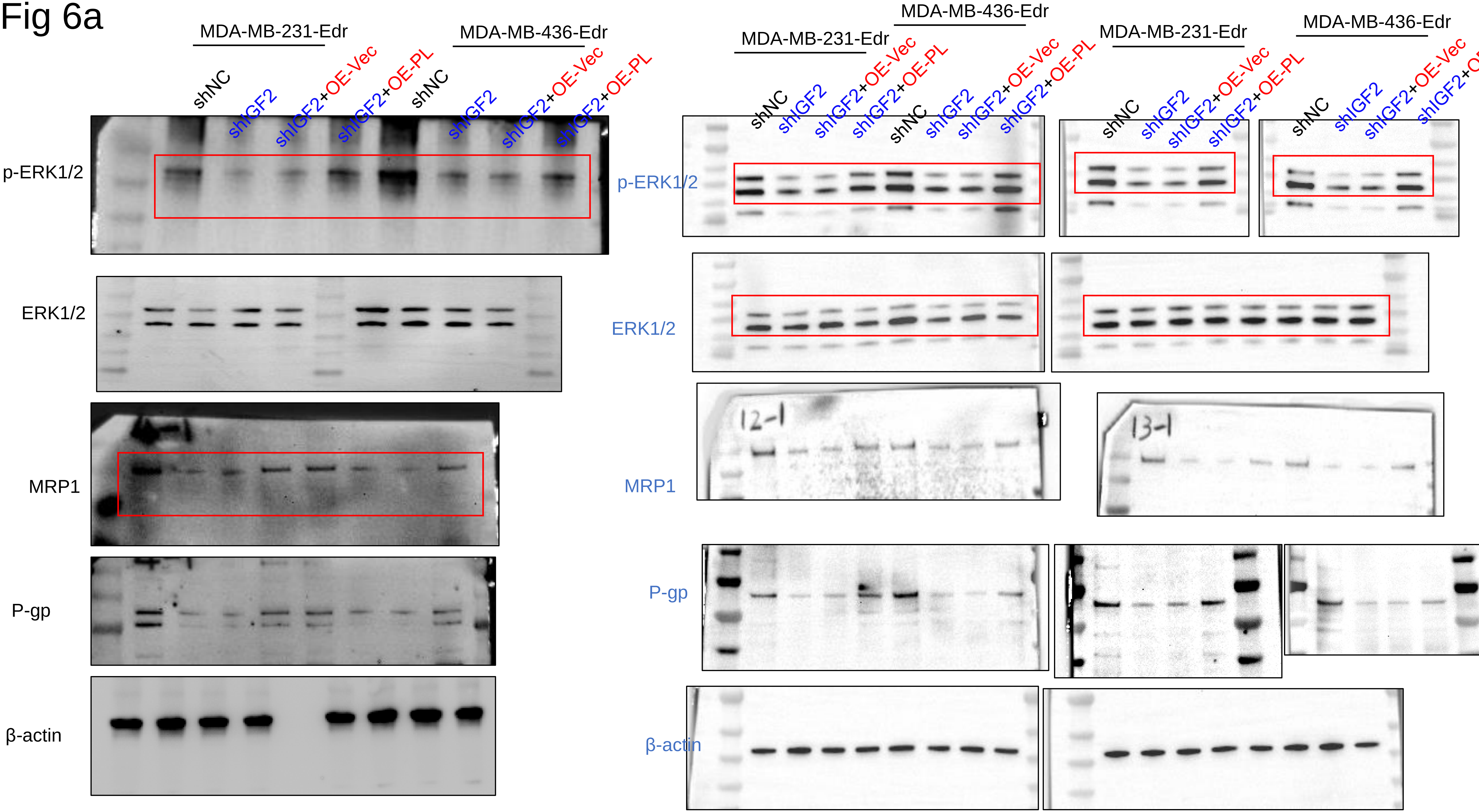
MDA-MB-231-Edr
MDA-MB-436-Edr
shNC
shIGF2
shIGF2+OE-Vec
shIGF2+OE-PL
p-ERK1/2
ERK1/2
MRP1
P-gp
β-actin
12-1
13-1

Fig 8c

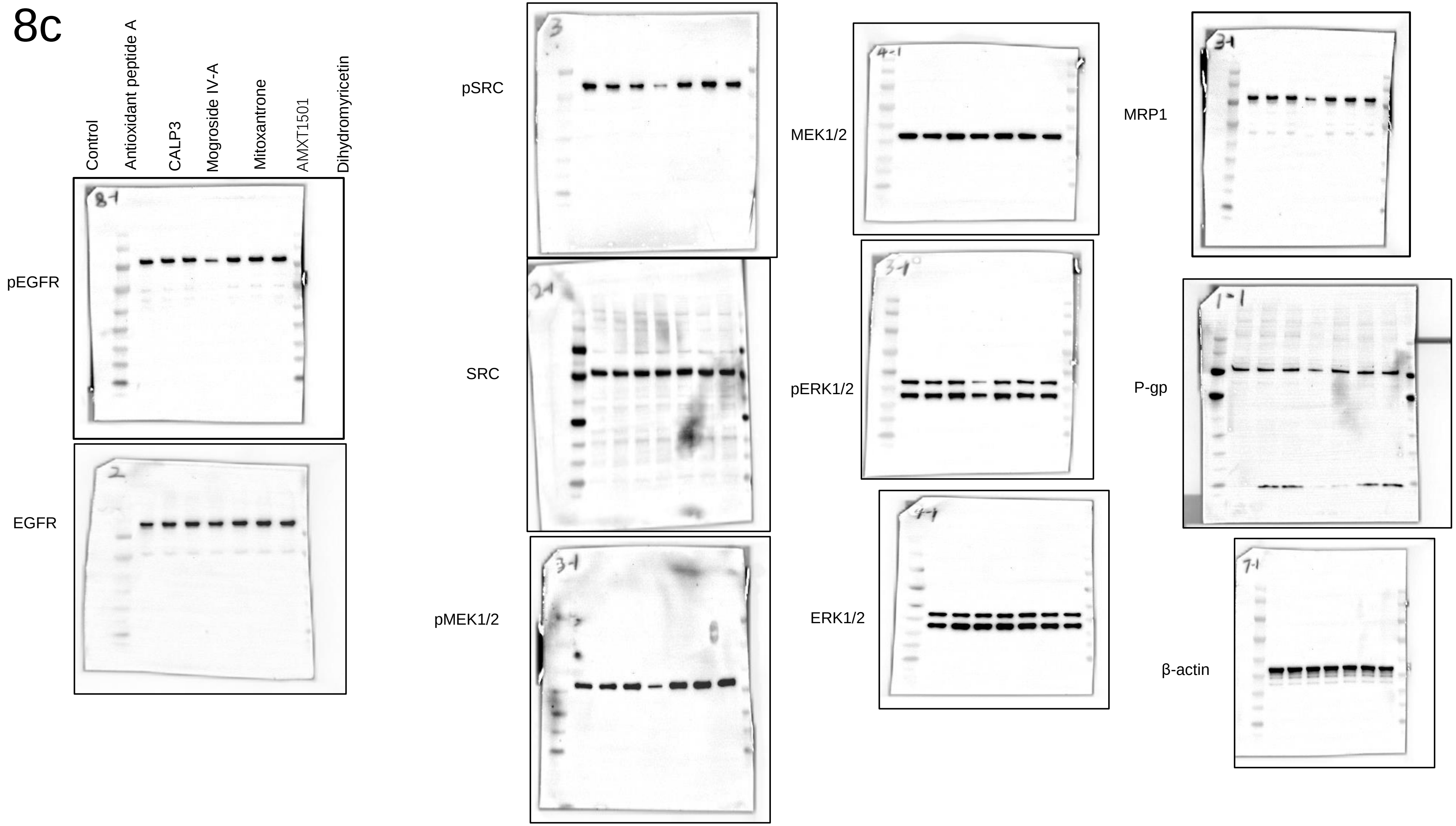
Control
Antioxidant peptide A
CALP3
Mogroside IV-A
Mitoxantrone
AMXT1501
Dihydromyricetin
pEGFR
EGFR
pSRC
SRC
pMEK1/2
MEK1/2
pERK1/2
ERK1/2
MRP1
P-gp
β-actin

Fig 10

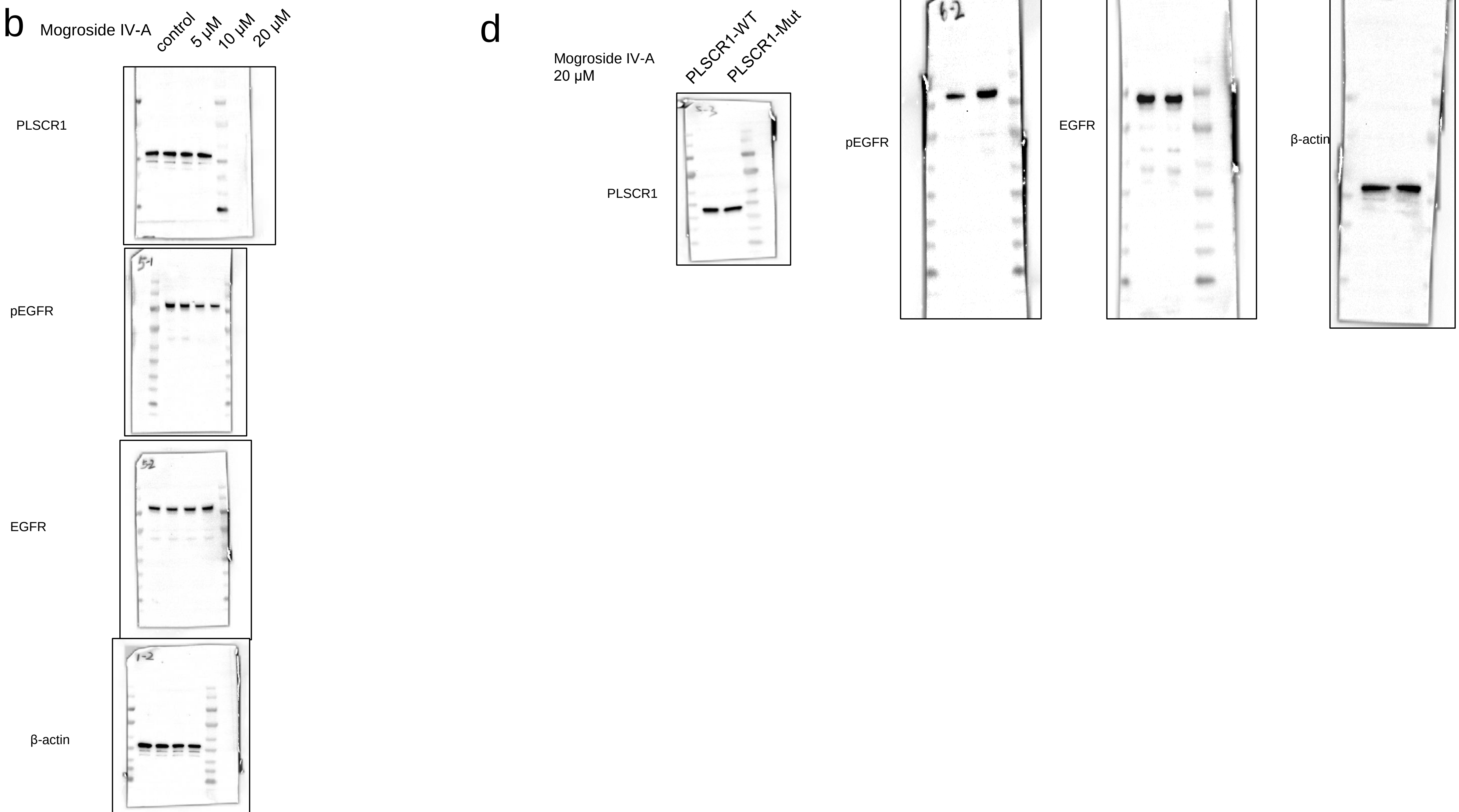
b
Mogroside IV-A
control
5 μM
10 μM
20 μM
PLSCR1
pEGFR
EGFR
β-actin
d
Mogroside IV-A
20 μM
PLSCR1-WT
PLSCR1-Mut
PLSCR1
pEGFR
EGFR
β-actin

Fig 10e

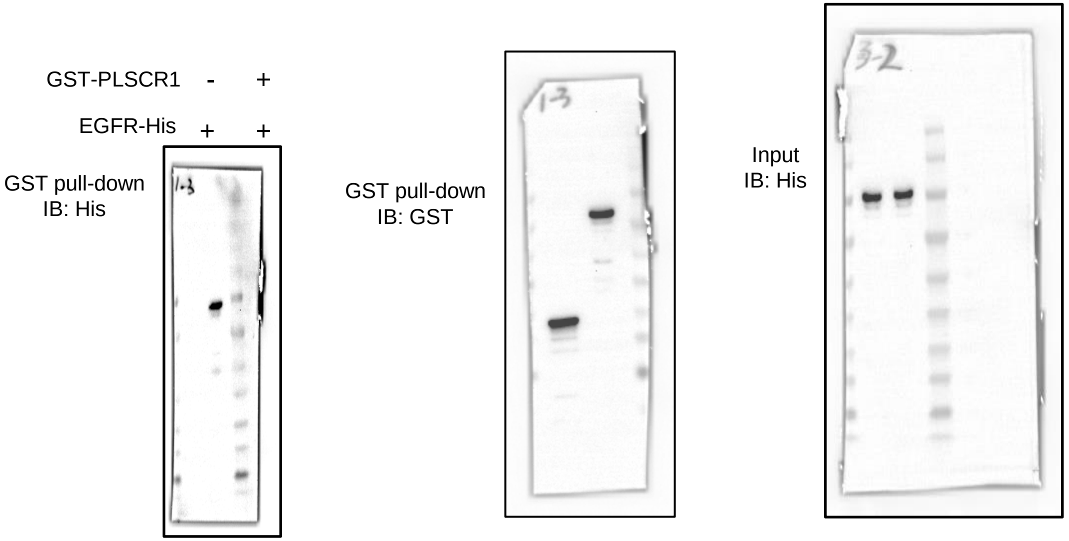
GST-PLSCR1 - +
EGFR-His + +
GST pull-down
IB: His
GST pull-down
IB: GST
Input
IB: His
