# Supplementary material for: PLSCR1 drives chemoresistance in TNBC via METTL3/IGF2BP3-mediated mRNA stabilization and EGFR-MAPK pathway activation
Source: Cell Death Dis. 2026 May 15;17(1):624. doi: 10.1038/s41419-026-08845-4 (PMC13347015; doi:10.1038/s41419-026-08845-4)

apoptosis 231-edr gate

PLot1：
Gate P1 on the FSC-A/SSC-A plot to enclose the main cell population.

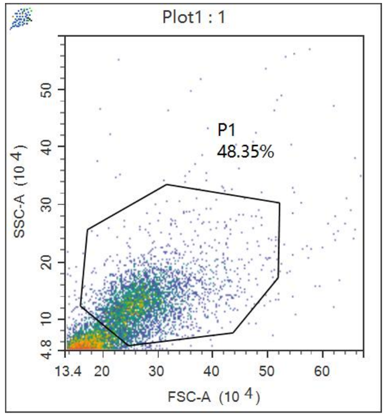

Plot2:
Analyze the apoptosis status of cells within gate P1 using the cross gate in the APC-H/7AAD-H plot.

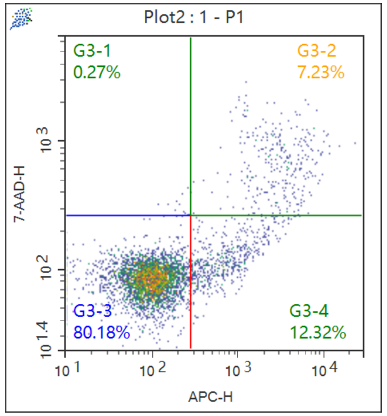

apoptosis 436-edr gate

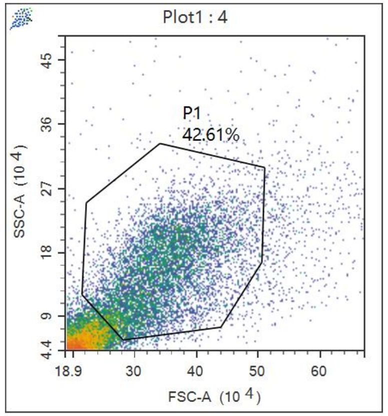

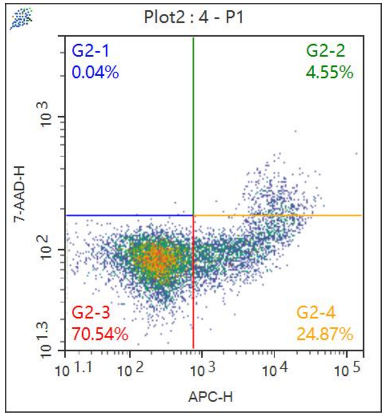

Supplement: Supplementary file 7 — Protocol for Flow Cytometry Gating Strategy [file 41419_2026_8845_MOESM7_ESM.pdf]
